# Partial reduction of interleukin‐33 signaling improves senescence and renal injury in diabetic nephropathy

**DOI:** 10.1002/mco2.742

**Published:** 2024-10-24

**Authors:** Li Chen, Chao Gao, Xingzhu Yin, Li Mo, Xueer Cheng, Huimin Chen, Chunjie Jiang, Bangfu Wu, Ying Zhao, Hongxia Li, Yanyan Li, Jiansha Li, Liangkai Chen, Qianchun Deng, Ping Yao, Yuhan Tang

**Affiliations:** ^1^ Department of Nutrition and Food Hygiene Hubei Key Laboratory of Food Nutrition and Safety Ministry of Education Key Laboratory of Environment and Health and MOE Key Lab of Environment and Health Key Laboratory of Environment and Health (Wuhan) Ministry of Environmental Protection State Key Laboratory of Environment Health (Incubation) School of Public Health Tongji Medical College Huazhong University of Science and Technology Wuhan China; ^2^ Oil Crops Research Institute of the Chinese Academy of Agricultural Sciences Hubei Key Laboratory of Lipid Chemistry and Nutrition and Key Laboratory of Oilseeds Processing Ministry of Agriculture Oil Crops and Lipids Process Technology National & Local Joint Engineering Laboratory Wuhan Hubei China; ^3^ National Institute for Nutrition and Health Chinese Center for Disease Control and Prevention Beijing Beijing China; ^4^ Shenzhen Center for Chronic Disease Control Shenzhen China; ^5^ Institute of Pathology Tongji Hospital Wuhan China; ^6^ Department of Pathology School of Basic Medicine Tongji Medical College Huazhong University of Science and Technology Wuhan China

**Keywords:** cellular senescence, diabetic nephropathy, interleukin‐33, senescence‐related secretory phenotype

## Abstract

Diabetic nephropathy (DN) is a frequent and costly complication of diabetes with limited understandings of mechanisms and therapies. Emerging evidence points to the important roles of interleukin‐33 (IL‐33) in acute kidney injury, yet its contribution to DN is still unclear. We here found a ubiquitous increase of IL‐33 and its receptor (ST2) in murine models and patients with DN. Surprisingly, both IL‐33 and ST2 knockdown aggravated renal lesions in DN, while overexpression of IL‐33 also exacerbated the condition. Further population‐based analyses revealed a positive correlation of IL‐33 expression with renal dysfunction in DN patients. Individuals with high IL‐33 expression‐related polygenic risk score had a higher DN risk. These findings confirmed the harmful effects of IL‐33 on DN. Conversely, endogenous and exogenous partial reduction of IL‐33 signaling conferred renoprotective effects in vivo and in vitro. Mechanistically, IL‐33 induced senescence by regulating cell cycle factors in HK‐2 cells, and accordingly senescence led to renal cell damage through the secretion of senescence‐related secretory phenotype (SASP) including IL‐33 and prostaglandins. Together, elevated IL‐33 accelerates cellular senescence to drive DN possibly by SASP production, while a partial blockage improves renal injury and senescence. Our findings pinpoint a possible and new avenue for DN interventions.

## Graphical abstract

Sustained hyperglycemic stimulation increases the production of IL‐33, which promotes the senescence of tubular epithelial cells through the regulation of cell cycle factors. The senescent cells then release SASP components, such as IL‐33 and prostaglandins, which further exacerbate cellular senescence and renal lesions.

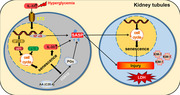

## 1 INTRODUCTION

Diabetic nephropathy (DN), a major complication of diabetes, is now a global concern.^1^ Statistically, DN contributes to 44.5% of end‐stage kidney disease (ESRD) cases in developed countries and increases the risk of cardiovascular diseases and mortality.^2^ Despite its prevalence, the underlying etiology of DN remains elusive and treatment options are limited. Current strategies, such as strict glycemic control, only slow the progression of the disease to a certain extent, leaving many patients to inevitably advance to chronic kidney disease and ESRD. This situation underscores the necessity of identifying a new pathogenesis and therapeutic strategy to prevent DN progress.^3^

Interleukin‐33 (IL‐33) is a cytokine of the IL‐1 family, present in various cell types such as epithelial cells, endothelial cells, and fibroblast cells.^4^ Once released, IL‐33 acts in an autocrine or paracrine manner through its receptor, ST2, to mediate tissue inflammation and repair responses.^5^ Traditionally linked to allergic conditions, IL‐33 has recently been recognized for its role in various chronic diseases such as obesity, osteoarthritis, and diabetes.^6^, ^7^, ^8^ Notably, the constitutive expression of IL‐33 and ST2 in the kidney positions it as a prospective target for treating kidney diseases.^9^ Earlier works revealed that the IL‐33‐type 2 innate lymphoid cell axis protected mice from ischemia‐reperfusion‐related acute kidney injury (AKI).^10^ However, high‐dose recombinant IL‐33 (rIL‐33) exacerbated renal lesions in a cisplatin‐induced AKI model and long‐term use could cause renal fibrosis.^11^, ^12^ In DN, although IL‐33 and ST2 levels were upregulated,^13^ the function of IL‐33 signaling remains unknown.

Senescent cells, characterized by irreversible cell cycle arrest, have garnered considerable attention. Due to their resistance to apoptosis and the persistent secretion of senescence‐associated secretory phenotype (SASP), senescent cells are increasingly recognized as a critical contributor to age‐related diseases. Senescence‐related renal dysfunction is commonly observed in DN patients and experimental models.^14^, ^15^ Importantly, studies have shown that removing p16‐positive cells in aged mice improved glomerulosclerosis,^16^ and knockout of p21 or p27 in T1D mice alleviated proteinuria and glomerular dilation.^17^, ^18^ These findings suggest that senescence plays a significant role in DN pathogenesis. Recent studies reported that IL‐33 caused naive T cellular senescence in mice during severe infection.^19^ Additionally, IL‐33 was also identified as a SASP component derived from senescent hepatic stellate cells.^20^ Given the complex interplay between IL‐33 signaling, cellular senescence, and DN pathogenesis, it is crucial to explore how modulating IL‐33 could influence senescence and disease outcomes.^20^, ^21^

In this study, we integrated experimental, genetic, and clinical evidence to demonstrate the deleterious role of IL‐33 in DN pathogenesis. Importantly, partial reduction of IL‐33 signaling, not complete deficiency, improved renal injury in DN, emphasizing the therapeutic potential for targeting the cytokines signaling pharmacologically. We also showed both excess and deletion of IL‐33 signaling aggravated cellular senescence, which was effectively ameliorated by genetic and pharmacological partial blockade of IL‐33 signaling in DN. In vitro studies further revealed the mediated effects of cellular senescence on the action of IL‐33 in cell injury.

## 2 RESULTS

### 2.1 Increased IL‐33 and ST2 levels in DN subjects

**FIGURE 1:**
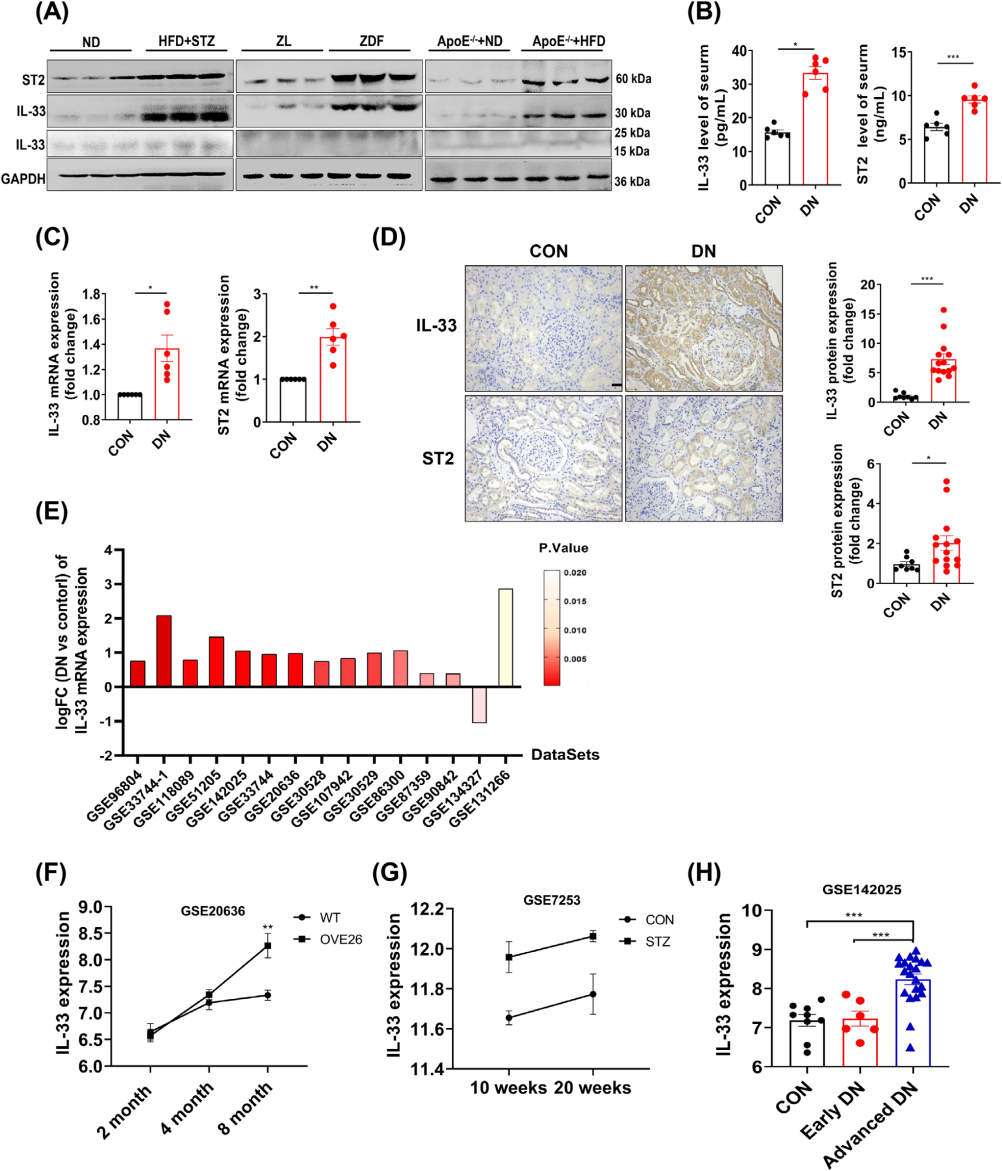
IL‐33 and ST2 levels are increased in human participants and animals with DN. (A) Western immunoblot analysis for IL‐33 and ST2 in kidney of DN mice (HFD+STZ), ZDF rats, or ApoE^−/−^ mice fed with HFD and respective control animals. GAPDH served as a loading control. The third blot shows the shorter IL‐33. (B and C) Serum IL‐33 levels and the expression levels of IL‐33 and ST2 mRNA in DN mice (HFD+STZ) and control mice (ND). (D) The protein expression of IL‐33 and ST2 in the paracancerous kidneys and the kidneys of DN patients was shown by immunohistochemistry (scale bar: 20 µm). (E) Fold change of IL‐33 mRNA levels in DN‐related models compared with control kidney tissue. Data from the GEO database. (F) At 2, 4, and 8 months, renal mRNA levels of IL‐33 in OVE26 diabetic mice and its control group (*n* = 4–7). (G) At 10 and 20 weeks, IL‐33 mRNA levels in the kidney of STZ‐induced diabetic rats and its control group (*n* = 3). (H) Renal IL‐33 mRNA expression in non‐diabetic, early‐stage, and advanced DN individuals. *p < 0.05, **p < 0.01, ***p < 0.001.

To investigate how IL‐33 signaling is involved in DN pathogenesis, we examined their transcript and protein levels in three kinds of DN models.^15^, ^22^, ^23^ Western blots showed significantly higher IL‐33 (full length) and ST2 in the kidney of DN models compared with the counterparts (Figure 1A). In type 2 diabetic (T2D) mice induced by a combination of a high‐fat diet (HFD) and streptozocin (STZ), both circulating and mRNA levels of IL‐33 and ST2 were also elevated (Figure 1B,C). Histologically, IL‐33 and ST2 positive spots were more expressed in the kidneys of DN patients than in paracancerous kidneys and mainly concentrated in the renal tubules (Figure 1D). To further confirm our findings, we analyzed 15 DN‐related datasets from the Gene Expression Omnibus (GEO) database. IL‐33 mRNA was upregulated in 14 datasets (Figure 1E). Notably, we observed the evolving increase of IL‐33 with DN progression in OVE26 mice (GSE20636) and STZ‐treated rats (GSE7253) (Figure 1F,G). Similarly, there was an evident increase of IL‐33 in advanced DN patients (urinary albumin‐to‐creatinine ratio (ACR) > 300 mg/g, glomerular filtration rate (eGFR) < 90 mL/min/1.73 m^2^), but not early DN (ACR between 30 and 300 mg/g, eGFR > 90 mL/min/1.73 m^2^) (Figure 1H).

### 2.2 IL‐33 or ST2 knockout exacerbates renal injury in DN mice

**FIGURE 2:**
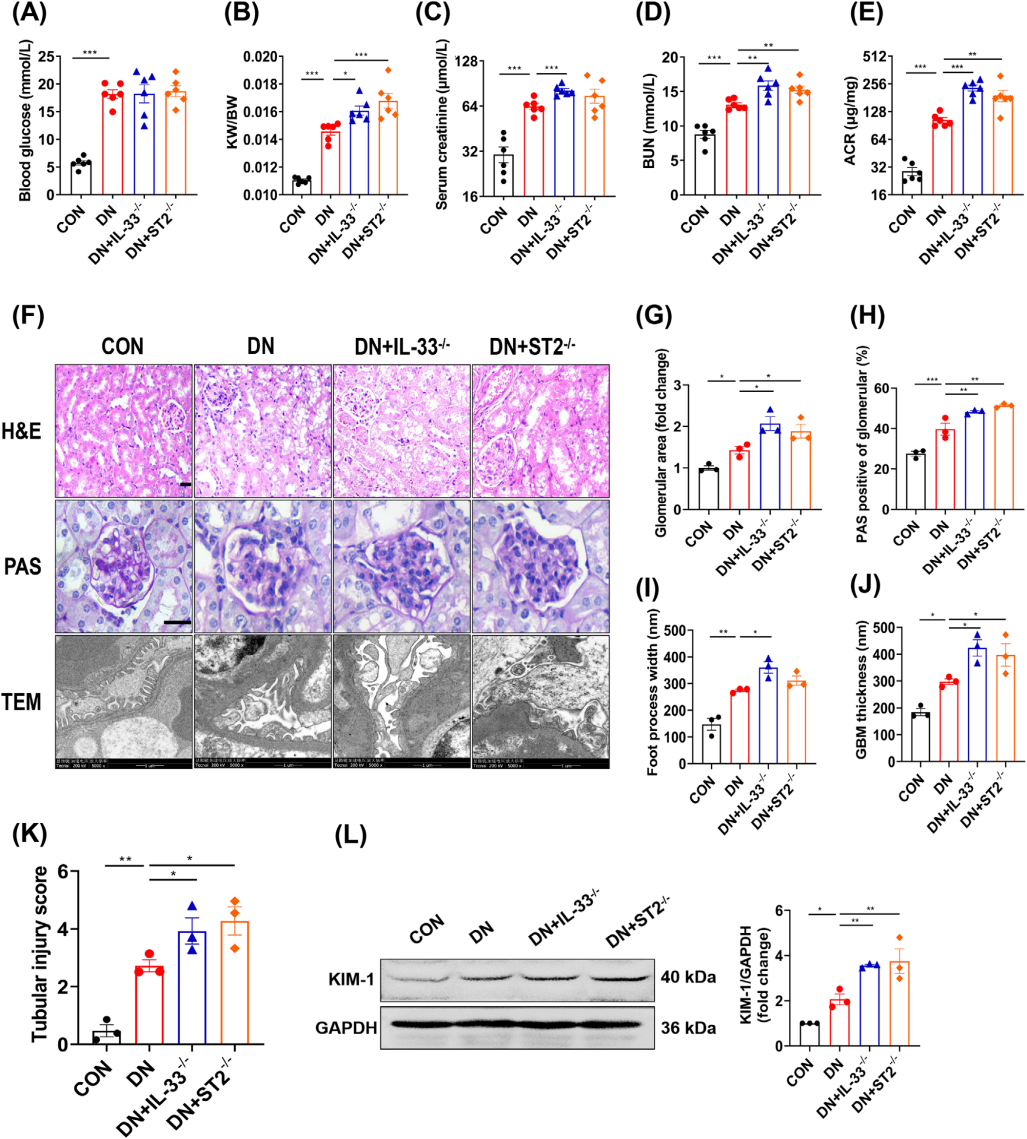
Renal injury is exacerbated in IL‐33^−/−^ or ST2^−/−^ DN mice. WT, IL‐33^−/−^, and ST2^−/−^ mice were continued to be fed for 12 weeks after they were determined to be diabetic. Control mice were continuously fed with ND. (A–E) FBG, renal‐body ratio, Scr, BUN, and ACR were monitored at the end of the experiment. (F) Representative images of kidney tissue stained with H&E and PAS (scale bar: 20 µm). Ultra‐structural changes in glomerular morphology were assessed by transmission electron microscopy (scale bar: 1 µm). (G and H) The glomerular area and PAS‐positive area were assessed in 3 mice with at least 10 glomeruli per mouse. (I and J) GBM thickness and foot process width were calculated in electron microscopic images of at least 5 fields of view per mouse. (K) The renal tubular injury score was estimated by H&E staining. (L) Immunoblot analysis for KIM‐1. *p < 0.05, **p < 0.01, ***p < 0.001.

Given the remarkable and common increase of IL‐33 and ST2 in DN, we constructed IL‐33 and ST2 knockout mice respectively (IL‐33^−/−^ or ST2^−/−^) (Figure S1A–C). In control mice, knockout of IL‐33 and ST2 had no significant impact on renal function (Figure S2A–H). Both IL‐33^−/−^ and ST2^−/−^ DN mice had a higher kidney‐body ratio (KW/BW), but there was no significant change in fasting blood glucose (FBG) compared with wild‐type (WT) DN mice (Figure 2A,B). Contrary to expectations, knockout resulted in worse renal outcomes, including higher levels of serum creatinine (Scr), blood urea nitrogen (BUN), and ACR (Figure 2C–E). Pathological examinations revealed that DN mice with IL‐33^−/−^ or ST2^−/−^ exhibited significantly disrupted renal morphology, characterized by epithelial cell desquamation, vacuolar degeneration, enlargement of glomerular Bowman's space, and expansion of the mesangium (Figure 2F–H). Furthermore, examination of the glomerular ultrastructure revealed thickening of the basement membrane and effacement of podocyte foot processes (Figure 2I,J). Besides glomerular damage, the tubular injury score and kidney injury molecular 1 (KIM‐1) were further increased in IL‐33^−/−^ and ST2^−/−^ DN mice (Figure 2K,L). The damage of ST2^−/−^ DN mice was similar to that of IL‐33^−/−^ DN mice, indicating that IL‐33 acted mainly through its receptor in DN.

### 2.3 RIL‐33 aggravates renal dysfunction in DN mice

**FIGURE 3:**
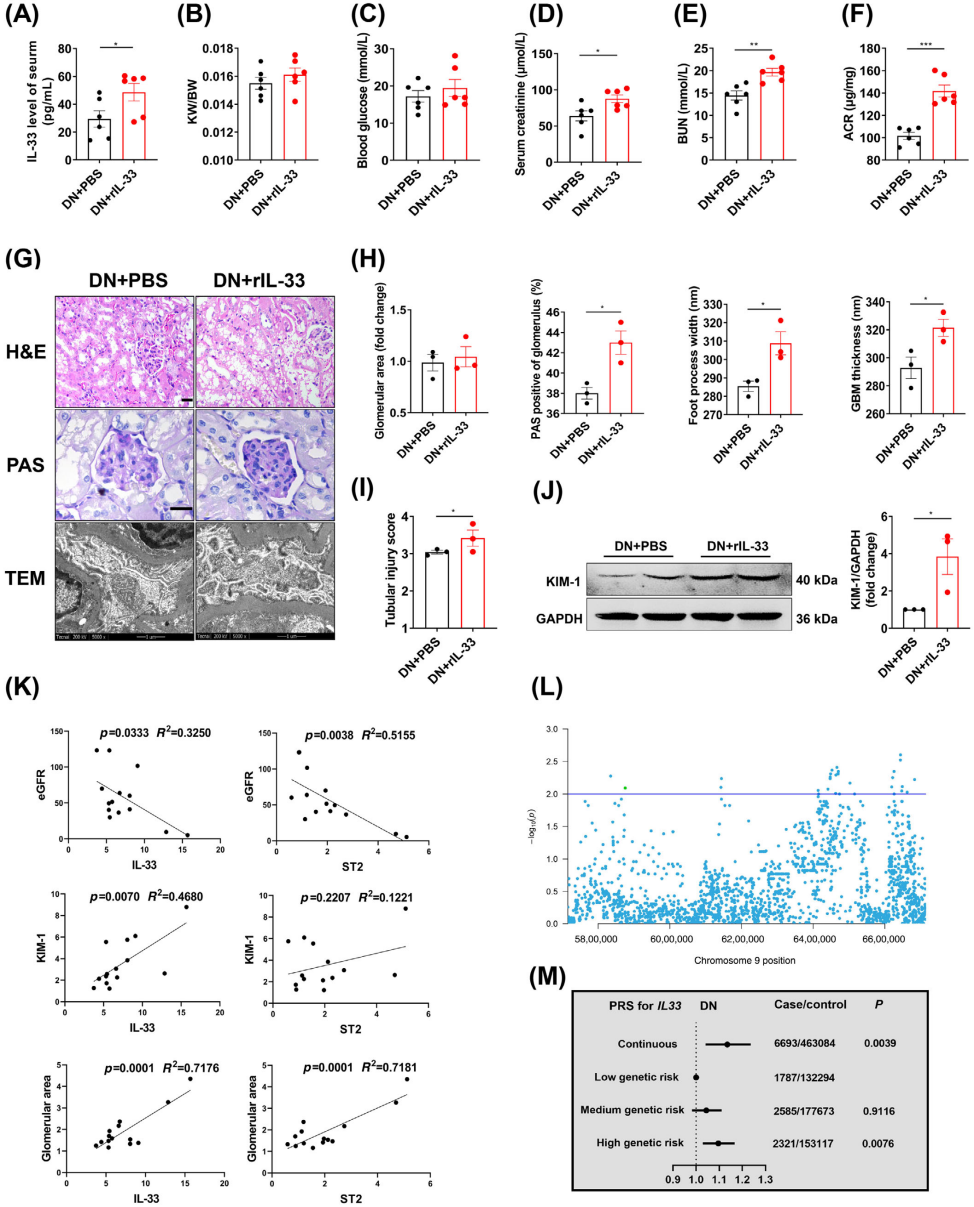
rIL‐33 administration aggravates renal injury in DN mice. Diabetic mice were intraperitoneally injected with PBS or rIL‐33 twice a week for 12 weeks and maintained with HFD feeding. (A) Serum IL‐33 levels were measured by ELISA at the endpoint of the experiment. (B–F) FBG, renal‐body ratio, Scr, BUN, and ACR were assessed. (G) Representative micrographs about DN‐related pathological indicators. (H) The statistics of glomerular area, PAS‐positive area, GBM thickness, and foot process width. (I) Renal tubular injury score based on the area of renal tubular injury in H&E images. (J) Renal lysates were processed for western immunoblot analysis for KIM‐1. (K) Correlation of eGFR, glomerular area, and KIM‐1 expression with IL‐33 level and ST2 level. (L) eQTL analysis between IL‐33 mRNA in whole blood and the SNPs of *IL33* nearby region (± 500 kb) using data provided by GTEx. The *x*‐axis showed the chromosomal positions and the *y*‐axis showed a −log_10_ *p* value; the significance threshold used in our analysis was *p *< 0.01. (M) OR (95% CI) in PRS associated with DN risk among participants with or without DN from UK biobank. *p < 0.05, **p < 0.01, ***p < 0.001.

The present contradictory results encouraged us to revisit the role of IL‐33. We administrated rIL‐33 to DN mice, which caused a 76% increase in serumal IL‐33 at the end of the experiment (Figure 3A). Administering rIL‐33 to DN mice worsened renal function (increased Scr, BUN, and ACR) (Figure 3B–F). Histomorphologically, periodic acid‐Schiff (PAS) staining showed mesangium expansion, and transmission electron microscope (TEM) images revealed the abnormal basement membrane and podocyte foot process in rIL‐33‐treated DN mice (Figure 3G,H). The increased renal tubular injury score and KIM‐1 expression indicated the negative effects of rIL‐33 on renal tubular in DN mice (Figure 3I,J).

To further determine the clinical relevance of the findings, we examined the expression of IL‐33, ST2, and KIM‐1, eGFR, and glomerular area from renal biopsies in DN patients (Table S2). IL‐33 showed a negative correlation with eGFR (*p *= 0.0333), and a positive correlation with the KIM‐1 levels (*p *= 0.0070) and glomerular area (*p *= 0.0001). ST2 also showed a negative correlation with eGFR (*p *= 0.0038) and a positive correlation with the glomerular area (*p *= 0.0001) (Figure 3K).

To search for genetic evidence in the population, we screened IL‐33 expression‐related single nucleotide polymorphisms (SNPs) by conducting the expression quantitative trait loci (eQTL) analyses in whole blood from the Genotype‐Tissue Expression (GTEx) (Figure 3L). Among 28 SNPs with *p *< 0.01, we obtained relatively independent 7 SNPs based on linkage disequilibrium (LD) (*r* ^2^ < 0.8) after excluding 11 SNPs negatively correlated with IL‐33 expression (Figure S3A,B). On account of the attractive role of polygenic risk score (PRS) in measuring the cumulative effect of multiple risk‐associated variants, we calculated IL‐33 expression‐related PRS among 6693 DN and 463,084 non‐DN participants from the UK biobank (Table S1). We found that PRS was positively associated with DN risk after adjusting age, sex, race, the first 10 primary components of ancestry, and genotype measurement batches (OR = 1.135, 95% confidence intervals (CIs): 1.041–1.236) (Figure 3M). Compared with participants with low genetic risk, those with a high genetic risk had a higher DN risk (OR = 1.096, 95% CIs: 1.029–1.167) (Figure 3M). In addition, similar results were obtained after excluding the SNPs associated with DN and then recalculating PRS (Figure S3C,D). These analyses indicated the high level of IL‐33 as a risk factor for DN.

### 2.4 Partial reduction of IL‐33 signaling attenuates renal damage in DN mice

**FIGURE 4:**
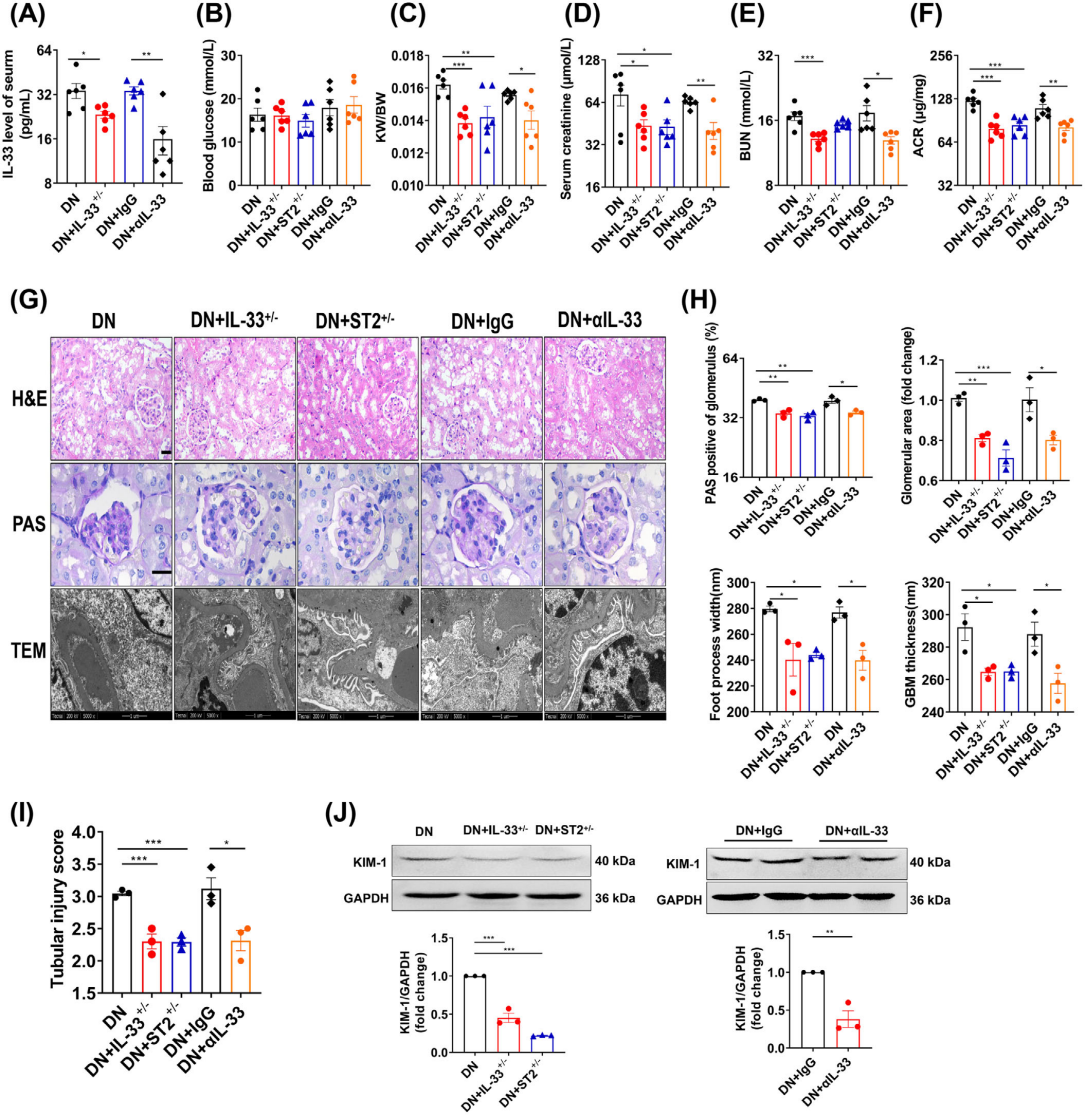
Renal senescence and aging are improved in IL‐33^+/−^ and ST2^+/−^ DN mice and. αIL‐33‐treated WT DN mice. WT mice considered diabetic were intraperitoneally injected with IgG or αIL‐33 twice a week for 12 weeks and maintained with HFD feeding. IL‐33^+/−^ and ST2^+/−^ DN mice were maintained with HFD feeding for 26 weeks. (A) The circulating IL‐33 level was measured by ELISA. (B and C) FBG and renal‐body ratio. (D‐F) Renal function was measured by Scr, BUN, and ACR. (G) Representative images of H&E, PAS staining (scale bar: 20 µm), and transmission electron microscopy (scale bar: 1 µm). (H) The statistics of pathological indicators in the glomeruli included glomerular area, PAS‐positive areas, GBM thickness, and foot process width. (I and J) Renal tubular injury score and KIM‐1 protein level were used to measure renal tubular changes. *p < 0.05, **p < 0.01, ***p < 0.001.

Previous research reported that the action of IL‐33 was sometimes contradictory within a similar pathology.^24^ Thus, we speculated the promotion role of IL‐33 dysregulation in this illness. We used neutralizing IL‐33 antibodies (αIL‐33) and heterozygous IL‐33 and ST2 mice (IL‐33^+/−^ and ST2^+/−^) to exogenously or endogenously reduce IL‐33 signaling in DN mice (Figure 4A). Compared with DN mice, αIL‐33‐treated and heterozygous DN mice exhibited decreased KW/BW, Scr, BUN, and ACR (Figure 4B–F). The improvement of the pathological indicators was also present in IL‐33^+/−^ and ST2^+/−^ DN mice or mice treated with αIL‐33, manifested as reduced glomerular volume and mesangial dilation, narrowed basement membrane, and wide foot process space (Figure 4G,H). The renal tubule‐related injury was effectively relieved (Figure 4I,J). Overall, partial reduction of IL‐33 signaling ameliorated kidney injury in DN, but not complete elimination.

### 2.5 Cellular senescence is involved in the role of IL‐33 in DN

Senescence is increasingly considered an important cause of DN. ^25^ In T2D mice, there is the greatest accumulation of senescent cells in the kidneys compared with the brain, heart, liver, and pancreas (Figure S4A). In DN patients, p16 expression showed a negative correlation with eGFR, and a positive correlation with the glomerular area (Figure S4B). In vitro, the injury of renal tubular epithelial cells was ameliorated by silencing cellular senescence regulators (p16 and p21) (Figure S4E–H), indicating the importance of cellular senescence in renal cell injury. Moreover, IL‐33 and ST2 showed well colocalization with senescent renal tubular epithelial cells in DN mice and patients (Figure S4C,D). We therefore investigated the effects of IL‐33 on renal senescence.

**FIGURE 5:**
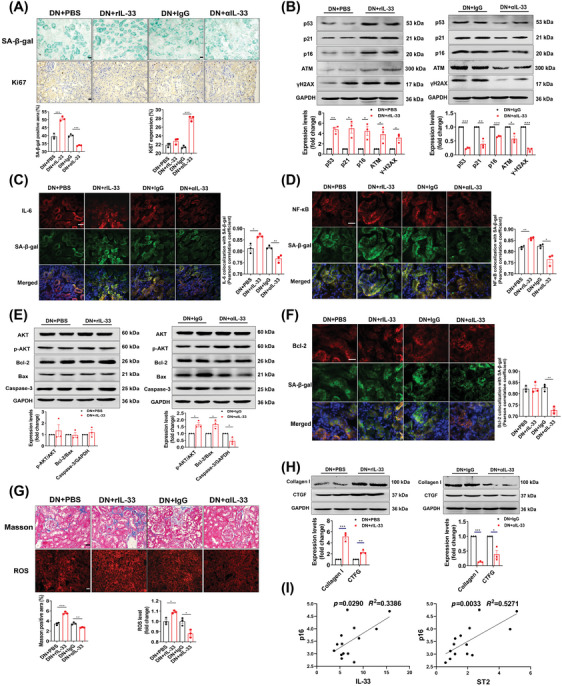
rIL‐33 exacerbates renal senescence and aging in DN mice. (A) Representative images of kidney tissue stained with SA‐β‐gal and Ki67. The statistical data were the ratio of positive area. (B) The protein levels of senescence markers were determined by immunoblots. Densitometric analysis of p53, p21, p16, ATM, and γH2AX. (C and D) Representative fluorescence colocalization images of IL‐6 (red) and NF‐κB (red) with SA‐β‐gal (C_12_FDG, green) in renal tissue. Colocalization statistics were shown as Pearson's correlation coefficient. (E) Protein levels in the cell apoptosis pathway, including AKT, p‐AKT, Bcl‐2, Bax, and Caspase‐3. (F) Colocalization of the prosurvival protein Bcl‐2 (red) with SA‐β‐gal (green). (G) The extent of fibrosis (Masson staining) and the level of oxidative stress in the kidneys (ROS fluorescent probe, 10 µM). (H) Fibrosis‐related protein levels including Collagen I and CTGF. (I) Correlation of p16 expression with IL‐33 and ST2 level in DN patients. Scale bar: 20 µm. *p < 0.05, **p < 0.01, ***p < 0.001.

RIL‐33 administration increased the senescence‐associated β‐galactosidase^+^ (SA‐β‐gal^+^) area, while αIL‐33‐treated DN mice had an occasional positive area (Figure 5A). The treatment of αIL‐33 also upregulated Ki67, and decreased senescence‐related proteins (p53, p21, and p16) and DNA damage response‐associated proteins (ATM and γ‐H2AX) (Figure 5A,B). The SASP, which represents the unique secreted factors from senescent cells, has been documented to disturb the microenvironment and promote disease advancement.^26^ Thus, we colocalized the IL‐6, a component of SASP, with senescent cells and found a significant decrease in merged fluorescent spots in αIL‐33‐treated DN mice (Figure 5C). Likewise, the colocalization of NF‐κB, a regulator of SASP,^27^ with SA‐β‐gal showed occasional positivity, in contrast to the evident colocalized positive spots in IgG‐treated DN mice (Figure 5D). Inversely, rIL‐33 significantly increased the production of SASP. Since resistance to apoptosis is responsible for the accumulation of senescent cells,^28^ we examined the apoptosis‐related pathways in the kidney. The protein levels of p‐AKT/AKT and Bcl‐2/Bax were increased and caspase‐3 was decreased in αIL‐33‐treated DN mice (Figure 5E). Importantly, the prosurvival protein Bcl‐2 was profoundly impaired in senescent cells (Figure 5F). However, the apoptotic pathway was not significantly changed in DN mice after adding rIL‐33, although Bcl‐2 was mildly increased in senescent cells. Moreover, αIL‐33 treatment also alleviated markers of renal aging, including renal fibrosis and oxidative stress (Figure 5G,H). For clinical relevance, we found positive correlations of IL‐33 and ST2 with p16 in DN patients (Figure 5I).

We further explore whether endogenous partial reduction of IL‐33 had the same antisenescence effects. After partial knockdown of IL‐33 and ST2, SA‐β‐gal was significantly reduced in DN mice, even though the Ki67 expression was not different (Figure S5A). Other senescence‐related and aging‐related markers were also normalized in IL‐33^+/−^ and ST2^+/−^ DN mice (Figure S5A–I). Conversely, complete knockout of IL‐33 and ST2 increased senescence markers in DN mice (Figure S6A–H). Strikingly, Bcl‐2 was heavily accumulated in senescent cells in IL‐33^−/−^ and ST2^−/−^ DN mice, suggesting IL‐33 signaling deletion resulted in severe resistance to apoptosis in the senescent kidney (Figure S6E,F).

### 2.6 Cellular senescence mediates the role of partial reduction of IL‐33 in cell injury in vitro

**FIGURE 6:**
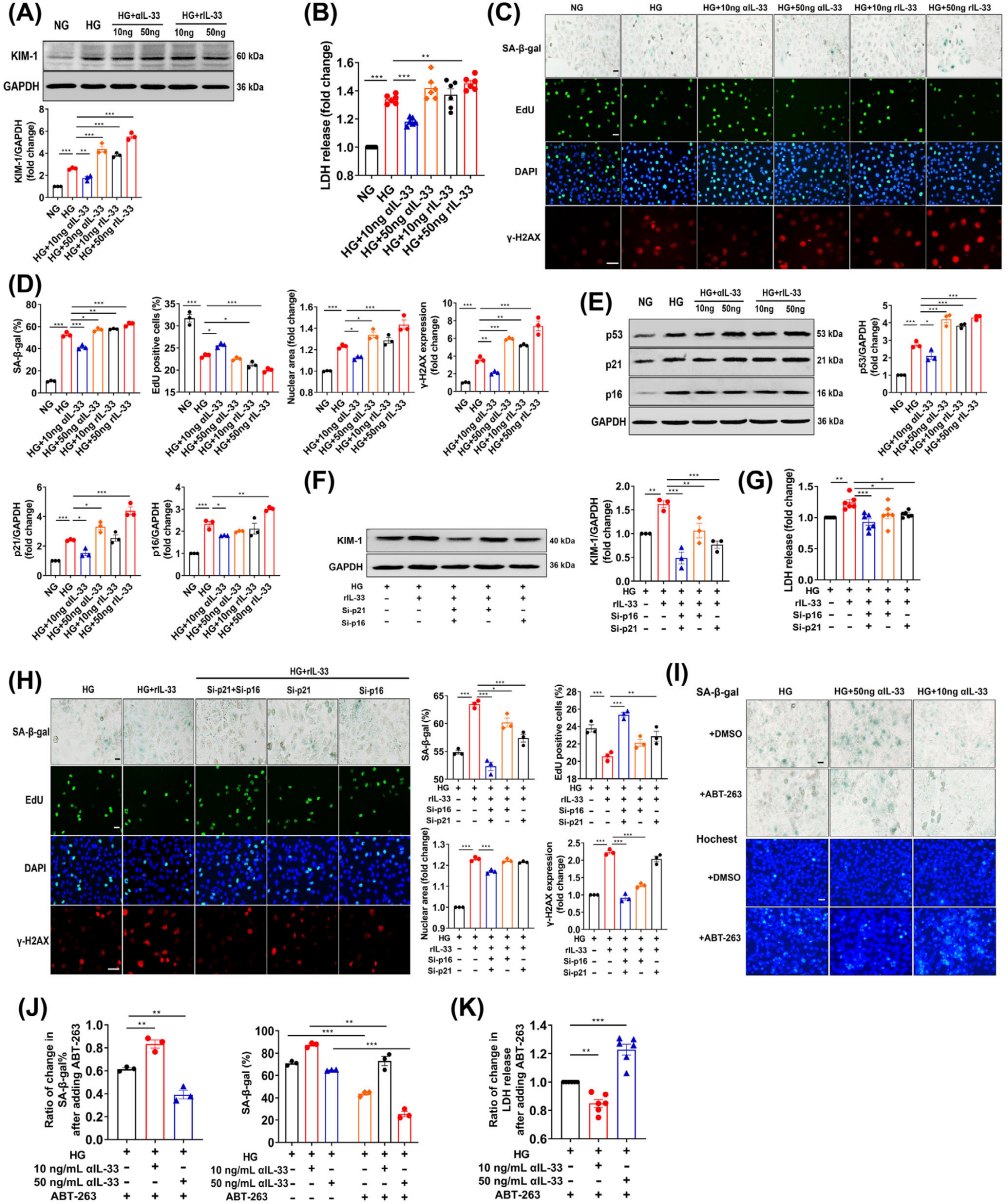
Partial reduction of IL‐33 alleviates cell injury and senescence, and cellular senescence mediates the role of IL‐33 in vitro. (A and B) KIM‐1 expression and LDH release in cells treated with different doses of αIL‐33 (10 or 50 ng/mL) and rIL‐33 (10 or 50 ng/mL) for 10 days. (C and D) cellular senescence‐related changes including the ratio of SA‐β‐gal^+^ and EdU^+^ cells, and fold change in nuclear area and γH2AX expression. (E) The protein levels of p53, p21, and p16 in HK‐2 cells. (F–H) Cell damage‐related (KIM‐1 expression and LDH release) and cellular senescence‐related changes (SA‐β‐gal, EdU, nuclear area, and γH2AX) in rIL33‐treated (50 ng/mL) cells with p16 and p21 silencing respectively or simultaneously. (I–K) SA‐β‐gal, Hochest (marked apoptotic cells), and LDH analyses of HK‐2 cells exposed to HG for 17 days and ABT‐263 (10 µM) for 1 day. Scale bar: 20 µm. *p < 0.05, **p < 0.01, ***p < 0.001.

Our results so far indicated that senescence may be involved in the role of IL‐33 in DN. We further explored the underlying mechanism in vitro. Because both senescence and the increased expression of IL‐33 and ST2 were located in the renal tubule (Figure S4D,E), human tubular epithelial cells (HK‐2) were exposed to high glucose (HG, 35 mmol/L) for 15 days to establish a model that mimicked in vivo. Treatments of αIL‐33 (50 ng/mL) or rIL‐33 (50 ng/mL) did not cause damage in cells exposed to normal glucose (NG, 5.5 mmol/L) (Figure S7A). The KIM‐1 expression and LDH release were increased with the increase of rIL‐33 concentration (Figure 6A,B). Similarly, cell damage was aggravated by 50 ng/mL of αIL‐33 but effectively reversed by 10 ng/mL dosage (Figure 6A,B). Besides, the antisenescence role of 10 ng/mL αIL‐33 was supported by the increased EdU^+^ cells, decreased SA‐β‐gal^+^ cells, γ‐H2AX^+^ cells, and nuclear area (Figure 6C,D), and down‐regulated p53, p21, and p16 (Figure 6E). However, both 50 ng/mL αIL‐33 and 10 or 50 ng/mL rIL‐33 accelerated the senescence of HK‐2 cells challenged by HG. Consistently, by endogenous regulation of IL‐33 expression, we found that both 20% and 80% reduction of IL‐33 improved cell damage and senescence, and these effects were enhanced with the decrease of IL‐33 (Figure S6B–E).

Further, we investigated whether IL‐33 caused renal cell damage through cellular senescence. In the process of cellular senescence, p21 and p16 are considered to be vital regulators.^29^ Therefore, we used RNA interference to silence both p16 and p21 in HK‐2 cells simultaneously, which blocked HG‐induced cellular senescence and injury (Figure S4G,H). More importantly, the increase in KIM‐1 expression and LDH release in HK‐2 cells induced by 50 ng/mL rIL‐33 was repressed by the simultaneous silence (Figure 6F,G), indicating cellular senescence mediated the role of IL‐33 in renal cell damage. Moreover, p16 silencing almost completely reversed cellular senescence and damage induced by rIL‐33 (Figure 6H), suggesting IL‐33 caused cellular senescence mainly through upregulating p16.

In addition, we explored the possible mechanisms by which the deletion of IL‐33 signaling aggravated cellular senescence. In senescent HK‐2 cells cultured for 17 days, we used ABT‐263, a senolytic, to clear the senescent cells. Consistent with the above in vivo experiments, ABT‐263 was more difficult to decrease the senescent cell damage and induce death after continuous treatment with 50 ng/mL αIL‐33, indicating IL‐33 signaling deletion resulted in resistance to apoptosis (Figure 6I–K).

### 2.7 SASP derived from senescent cells contributes to renal cell damage

**FIGURE 7:**
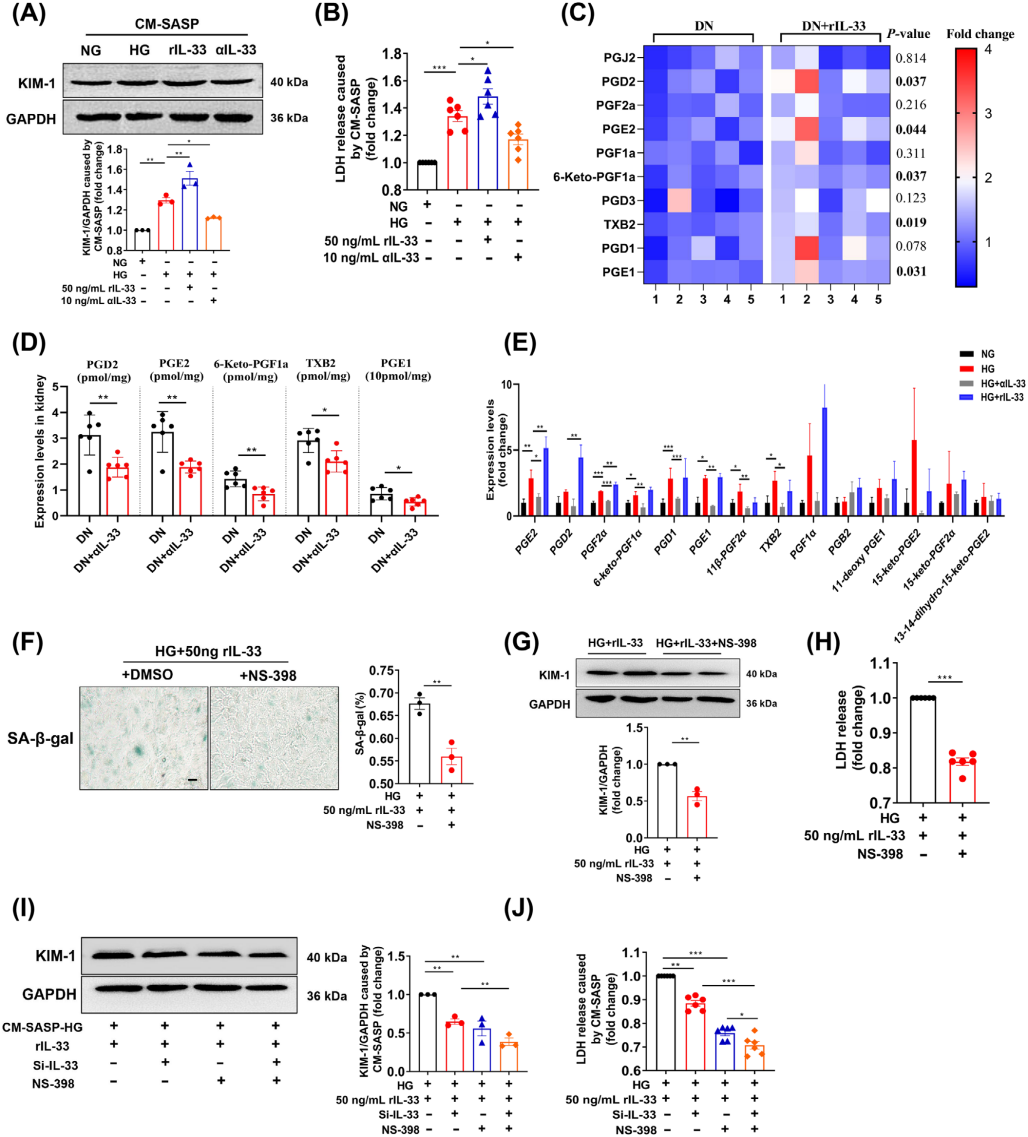
SASP derived from senescent cells contributes to renal cell damage. (A and B) KIM‐1 expression and LDH release in young HK‐2 cells treated with different CM‐SASP for 2 days. (C and D) Prostaglandins levels in DN mice with rIL‐33 or αIL‐33. (E) Prostaglandin levels in SASP of senescent HK‐2 cells (n = 3). (F–H) SA‐β‐gal^+^ cell, KIM‐1 expression, and LDH release in HK‐2 cells treated with COX‐2 inhibitor NS‐398 (10 µM) for 5 days (scale bar: 20 µm). (I and J) KIM‐1 expression and LDH release in young HK‐2 cells treated with different CM‐SASP (Si‐IL‐33 and NS‐398) for 2 days. *p < 0.05, **p < 0.01, ***p < 0.001.

We next investigated how cellular senescence caused renal injury. Conditioned medium (CM) was collected from IL‐33‐induced senescent cells to incubate young HK‐2 cells. Figure 7A,B presented the increase of LDH release and KIM‐1 expression in young HK‐2 cells exposed to the CM‐(HG+rIL‐33) compared with CM‐HG exposed cells, indicating SASP caused young cell injury. Recent research reported that senescence entailed significant changes in lipid metabolism, especially in oxylipin. In the current study, lipid profiles showed that some oxylipin in the kidney of DN mice increased significantly after administration with rIL‐33 (Table S3), including prostaglandin D2, prostaglandin E2, prostaglandin E1, thromboxane B2, and 6‐Keto‐prostaglandin F1a (Figure 7C). Conversely, αIL‐33 treatment decreased the levels of these oxylipins (Figure 7D). Further, some prostaglandins in the supernatant of senescent HK‐2 cells were also elevated, indicating prostaglandins were SASP secreted by senescent renal tubular epithelial cells (Figure 7E). Also, these prostaglandins were regulated by prostaglandin synthases (cyclooxygenase‐2, COX‐2). The COX‐2 inhibitor NS‐398 relieved senescence and damage of HK‐2 cells caused by HG and rIL‐33 (Figure 7F–H). Compared with the exposure to CM‐(HG+rIL‐33), the damage of young HK‐2 cells was reduced when exposed to CM‐(HG+rIL‐33+NS‐398) and CM‐(HG+rIL‐33+Si‐IL‐33) (Figure 7I,J). Moreover, when IL‐33 and COX‐2 were inhibited simultaneously, the damage of young HK‐2 cells was further reduced (Figure 7I,J).

## 3 DISCUSSION

This study integrates clinical, genetic, and experimental data to highlight the complex role of IL‐33 in DN. Despite IL‐33 signaling being ubiquitously elevated in DN, its deletion unexpectedly exacerbated renal lesions rather than reversing the abnormalities. We further demonstrated its deleterious role in DN pathogenesis. In contrast, both endogenous and exogenous partial blocking of IL‐33 effectively ameliorated DN. Mechanistically, cellular senescence was involved in the pathways underlying the effect of IL‐33 on DN.

The present study showed increased IL‐33 and ST2 levels in renal and circulating proteins across multiple rodent models and human DN subjects, including T2D‐related DN and atherosclerosis‐related DN. Consistent with our findings, elevated IL‐33 and ST2 levels were presented in the kidney of DN rats.^30^ Multiple studies also reported increased circulating levels of IL‐33 and ST2 in DN patients.^13^ Our study further provided histological evidence that IL‐33 and ST2 were highly expressed in human kidneys with DN. This common feature was also found in 14 DN‐related datasets from the GEO database. Of note, IL‐33 mRNA was significantly upregulated in advanced DN, but not increased in early DN. Similarly, the IL‐33 level was evolving increased with the progression of DN mice. The above observations indicated that IL‐33 might be a contributor to DN progress.

Contrary to our initial hypothesis, both IL‐33^−/−^ and ST2^−/−^ DN mice showed worsened renal outcomes, suggesting a potential protective role of IL‐33 against DN. Likewise, the increased proteinuria levels in ST2^−/−^ T1D mice were reported previously.^31^ However, this notion was challenged by the detrimental effects observed following rIL‐33 administration. Our population study further provided genetic evidence that IL‐33 expression‐related PRS was positively associated with DN risk. More importantly, there is a positive correlation of IL‐33 and ST2 protein levels with kidney dysfunctions in DN patients. Conversely, a study demonstrated that the administration of IL‐33 improved renal damage in T1D mice.^32^ It is important to note that this study utilized STZ‐induced T1D mice and the IL‐33 intervention was conducted for 6 days. Overall, our comprehensive clinical and experimental research provides strong evidence for the harmful effects of IL‐33 on T2D‐related DN. Under these circumstances, it is confusing that IL‐33 signaling deficiency cannot confer the intended effects. Indeed, the fundamental biological effect of IL‐33 is involved in tissue development, homeostasis, and repair.^33^ Especially for obesity, IL‐33^−/−^ mice dysfunctioned in the maintenance of adipose tissue homeostasis and accordingly increased weight and visceral adipose tissue mass after HFD feeding.^34^ Thus, total blockade of IL‐33 signaling could be potentially hazardous, despite excess IL‐33′s undesirable effects on DN.

We therefore applied neutralizing antibodies to lower circulating levels of IL‐33 in DN mice. Functionally, αIL‐33 treatment improved renal impairment in DN mice, similar to the effect of partial IL‐33 and ST2 knockout on DN mice. In vitro, exogenous or endogenous partial blockade of IL‐33 signaling consistently elucidated a reversed effect on cell injury. A study also indicated that a 50% suppression of IL‐33 signaling by calycosin conferred a renoprotective effect on DN.^30^ These findings not only settled the above paradoxical observation of IL‐33′s role, but also proposed a new avenue for DN intervention. Excitingly, αIL‐33 has been used in many clinical trials. In a phase 2a trial, itepekimab, a monoclonal antibody targeting IL‐33, reduced exacerbation rate and improved lung function in former smokers with chronic obstructive pulmonary disease.^35^ Wechsler et al.^36^ also reported itepekimab improved asthma control and lung function in patients with moderate‐to‐severe asthma. Recent clinical trials showed that blocking the IL‐33 pathway may mitigate glomerular endothelial inflammation in DN.^37^, ^38^ However, its dose needs to be considered to avoid side effects and adverse responses. Our results highlighted the beneficial effects of partial reduction of IL‐33 signaling on DN, where a 20−80% reduction can improve damage.

Mechanistically, we displayed that IL‐33 caused cellular senescence and renal aging, while a partial reduction of IL‐33 signaling suppressed senescence. Compelling evidence in vitro further identified IL‐33 as an inducer of HK‐2 cellular senescence. This important and novel mechanism for IL‐33 has also been proposed by a recent study, in which IL‐33 was reported to induce naive T cell aging by inducing thymic involution in mice during severe infection.^19^ Furthermore, we found that IL‐33 induced renal cell damage through accelerating cellular senescence mainly mediated by upregulating p16. Consistently, the regulation of IL‐33 on cell cycle factors also has been documented in pancreatic cancer cells.^39^ Interestingly, IL‐33 was reported to suppress cell proliferation in quiescent NIH‐3T3 cells, while stimulating replication in proliferating cells.^40^ Thus, it is plausible that excess IL‐33 may act on senescent cells to further stabilize their quiescent state by regulating cell cycle factors.

**FIGURE 8:**
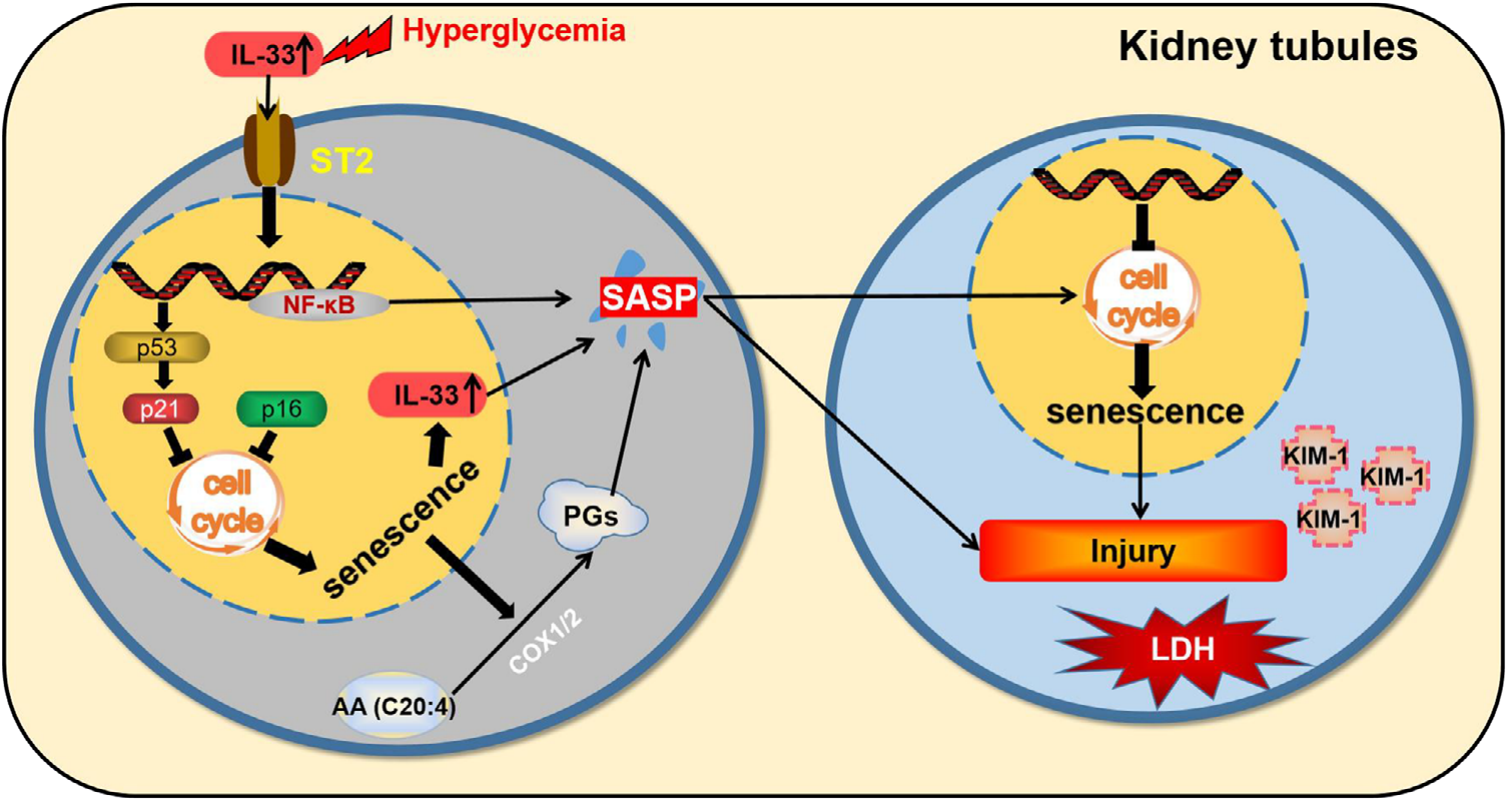
Mechanism flow chart. Sustained hyperglycemic stimulation leads to an increased production of IL‐33, which in turn accelerates senescence in tubular epithelial cells. The senescent cells then release SASP components, including IL‐33 and prostaglandins, which further amplify cellular senescence and exacerbate renal lesions.

Additionally, IL‐33 levels were increased in senescent cells of DN patients, mice, and HK‐2 cells. This situation closely resembled that IL‐33 was recently identified as a SASP component derived from senescent hepatic stellate cells.^20^ In our study, CM‐SASP‐derived senescent cells with IL‐33 silence reduced the damage of young cells, supporting IL‐33 as the SASP of senescent HK‐2 cells. What is more, rIL33 induced the increase of other SASP components in senescent cells, such as prostaglandins. We further observed that prostaglandins as the SASP were responsible for the effect of senescent cells on renal cell damage. Of special importance, cellular senescence, cell damage, and excess IL‐33 and ST2 expression were all chiefly concentrated in the renal tubules. Thus, we speculated that there was a loop where IL‐33 promoted the senescence of tubular epithelial cells, followed by SASP components including IL‐33 and prostaglandins from senescent cells further exacerbated senescence and renal lesions presumably by autocrine and paracrine in DN (Figure 8).

The complete loss of IL‐33 signaling also led to a massive accumulation of senescent cells. Of note, after IL‐33 and ST2 knockdown, the antiapoptotic pathway was markedly upregulated in the kidney, and prosurvival proteins were largely expressed in senescent cells. Complete deletion of IL‐33 signaling may render senescent cells incapable of apoptosis, since IL‐33 was linked to apoptosis in various models, such as osteosarcoma cells^41^ and diabetic ovarian injury.^42^ Notably, IL‐33^−/−^ mice challenged with *the O. tsutsugamushi* Karp strain displayed a significantly higher level of Bcl‐2 in the kidney.^43^Our in vitro results showed that the ability of senolytic (ABT‐263) to induce the death of senescent cells was decreased when the IL‐33 signaling was completely blocked. These findings indicated that complete IL‐33 deficiency might enhance the antiapoptotic ability of senescent cells and consequently lead to the accumulation of senescent cells in DN. This assumption was also supported by our observations that the prosurvival protein of senescent cells was decreased when we partially reduced IL‐33 signaling in DN.

This study has some strengths. To explore the role of IL‐33 in DN, we used knockout mice and injection of recombinant protein and neutralizing antibodies to regulate IL‐33 levels in vivo and further verified our results with population‐based data. There are also some limitations. First, although we found an extensive therapeutic range for the IL‐33 blockage, a better definition of “partial” reduction needs to be confirmed in future research. Second, the mechanism of IL‐33 promoting cellular senescence needs to be further explored. Finally, we found that complete loss of circulating IL‐33 could potentially pose a detrimental effect on DN. The conditional knockout of IL‐33 specifically in renal tubular epithelial cells may further elucidate the role of IL‐33 in DN.

In summary, we identify a previously unknown, but fundamental function of IL‐33 as a potent factor controlling renal senescence and injury in DN. The pharmacologically partial reduction of IL‐33 levels under DN conditions, through the use of neutralizing antibodies, conferred beneficial effects on renal dysfunction potentially by the antisenescence pathway. Our findings propose a promising new avenue for ameliorating DN.

## 4 MATERIALS AND METHODS

### 4.1 Animal experimental design

Eight‐week‐old male wild‐type C57BL/6 mice were procured from Charles River. IL‐33^−/−^ and ST2^−/−^ C57BL/6 mice were kindly provided by the Department of Immunology at Tongji Medical College. Mice were divided into two groups and fed either a 60% HFD or a normal diet (ND) for 12 weeks. Mice on HFD were subsequently injected intraperitoneally with 50 mg/kg of STZ (Sigma–Aldrich; S0130) dissolved in citrate buffer (pH 4.5) daily for 5 consecutive days, while control mice received a vehicle solution.^15^ One week postinjection, mice with a glucose concentration of over 11.1 mmol/L were regarded as diabetic. After the diabetic model was produced, all mice were maintained for an additional 12 weeks of continued HFD or ND feeding. Meanwhile, WT diabetic mice were administered with αIL‐33 (37 µg/kg) (R&D Systems; AF3626) or rIL‐33 (12.5 µg/kg) (MedChemExpress; HY‐P7218), and control mice were injected with control IgG or PBS, twice a week.

Additional diabetes models involving Zucker lean (ZL) rats, Zucker diabetic fatty (ZDF) rats, and ApoE^−/−^ mice followed established protocols.^22^, ^23^ We obtained male ZL rats and ZDF rats from Vital River Laboratories at the age of 6 weeks. Following an 8‐week feeding period, the animals were sacrificed for further analysis. Additionally, 6‐week‐old male ApoE^−/−^ mice and their littermate control ApoE^+/+^ mice were fed an HFD (21% fat and 1.25% cholesterol) or ND, respectively, for 16 weeks.^44^ The Tongji Medical College Council on Animals Care Committee approved all animal procedures carried out in this study.

### 4.2 Human kidney tissues and analysis

Renal biopsies from 14 DN and 8 non‐diabetic patients were obtained from the Tongji Hospital, Huazhong University of Science. Informed consent was obtained from each patient, and the patient's clinical biochemical data were extracted from medical records. The non‐diabetic samples consisted of peritumoral, normal kidney tissues. These tissues were fixed in formalin, embedded in paraffin, and then sectioned at a thickness of 2 µm for immunohistochemistry (IHC) staining (the dilution ratio: 1:100) of IL‐33 (Proteintech; 12372‐1‐AP), ST2 (Proteintech; 11920‐1‐AP), p16 (Proteintech; 10883‐1‐AP), and KIM‐1 (ABclonal; A2831), or immunofluorescence colocation between IL‐33 or ST2 with C_12_FDG (Invitrogen; I2904, 33 µM). Quantitative analysis of protein expression was performed using Image‐Pro Plus software, and the percentage positive staining of 5–10 images (×200 magnification) in each specimen was calculated.

Linear regression models were applied to examine the associations among IL‐33, ST2, p16, eGFR, KIM‐1, and glomerular area. The determination coefficient (*R* ^2^) was used to assess the power to which the independent variable explains variations in the dependent variable.

### 4.3 Cell culture

HK‐2 cells were purchased from Procell and maintained in DMEM/F12 (Gibco) supplemented with 10% fetal bovine serum, 100 U mL^−1^ penicillin/streptomycin (Gibco) at 37°C in a humidified atmosphere containing 95% air and 5% CO_2_. The cells were seeded in 6‐well plates with NG (5.5 mM glucose) or HG (35 mM glucose) medium for 5 days and then treated with αIL‐33 (10 or 50 ng/mL) or rIL‐33 (10 or 50 ng/mL) for 10 days.

### 4.4 Plasma biochemical and histological analysis

FBG was measured from mouse tail veins using a blood glucose meter (Roche Diagnostics). Scr, BUN, and ACR were measured with commercially available assay kits (Jiancheng Bioengineering Institute). Renal samples were subjected to standard staining including hematoxylin–eosin (H&E), PAS, and Masson's trichrome stain. The glomerular area and the extent of the mesangial matrix were assessed by the PAS satin. Tubular damage including epithelial cell desquamation, vacuolar degeneration, swelling, and necrosis was scored: 0: tubular damage area < 10%; 0−1: 10−25% of the tubular area was affected; 1−2: 25−50% of the tubular area was affected; 2−3: 50−75% of the tubular area was affected. 3−4: 75−90% of the tubular area was affected. 4−5: >90% of the tubular area was affected.

For transmission electron microscopy, small fragments of the renal cortex were fixed in 2.5% glutaraldehyde and embedded in Araldite. Ultrathin sections (80‐100 nm) were cut from the tissue using an ultramicrotome (Leica EM UC7). The thin slices were placed on a copper mesh grid, stained, and examined under a Tecnai G220 TWIN TEM.

### 4.5 SA‐β‐gal staining

Frozen kidney sections and cells were utilized to assess SA‐β‐gal activity using a commercially available kit (Beyotime Biotechnology), following the instructions provided by the manufacturer. The samples were incubated with a detection solution at 37°C overnight, and the presence of blue staining was indicative of SA‐β‐gal positive cells. Quantification was performed via light microscopy and Image J.

### 4.6 Immunofluorescence and IHC

The renal tissue or cell was fixed with 4% paraformaldehyde for 15 min. After washing three times with PBS, cells were permeabilized with 0.1% Triton X‐100 for 15 min and then washed again with PBS 3 times. After being blocked with 5% normal goat serum, the samples were incubated overnight with primary antibodies at 4°C (the dilution ratio: 1:100–200), including IL‐33, ST2, Bcl‐2 (Proteintech; 66799‐1‐lg), NF‐κB (Cell Signaling Technology; 8242s), IL‐6 (Abcam; ab259341), and γH2AX (ABclonal; AP0099). The following day, the tissue was labeled with secondary antibodies (Thermo Scientific; 1:800) for 1 h and then washed three times with PBS. DAPI was applied for 10 min and subsequently photographed under a fluorescent microscope (Olympus).

Paraffin‐embedded sections of kidney tissue from mice or patients with diabetes were used for IHC. After rehydration and blocking, tissue sections were exposed to the following primary antibodies overnight, followed by incubation with an HRP‐conjugated secondary antibody. After signaling amplification using the TSA Biotin System (PerkinElmer), the slides were developed with a VECTASTAIN Elite ABC Kit and ImmPACT DAB Peroxidase Substrate (Vector Laboratories).

### 4.7 Immunoblots

The cultured cells and renal cortex samples were homogenized using an ice‐cold lysis buffer and subsequently quantified using a BCA protein assay kit (Beyotime Biotechnology). The protein was then separated and transferred onto PVDF membranes (Millipore). After the membranes were blocked in 5% skim milk for 1 h at room temperature, they were incubated with primary antibodies overnight at 4°C (1:1000), including p53 (Cell Signaling Technology; 2524), p21 (Proteintech; 27296‐1‐AP), p16 (Proteintech; 10883‐1‐AP), ATM (ABclonal; A19650), γH2AX (ABclonal; AP0099), p‐AKT (Cell Signaling Technology; 4060S), AKT (Cell Signaling Technology; 4691S), Bcl‐2 (Proteintech; 66799‐1‐lg), Bax (Proteintech; A11931), and caspase‐3 (Cell Signaling Technology; 9662s), followed by incubation with the corresponding secondary antibodies (Cell Signaling Technology; 1:10000) for 1 h at room temperature. The density of each target band was quantified by Image Pro‐Plus 6.0 software and normalized to GAPDH (Proteintech; 60004‐1‐Ig) as optical density. All sample sizes of animals or cells were greater than or equal to 3.

### 4.8 Enzyme‐linked immunosorbent assay

Enzyme‐linked immunosorbent assay (ELISA) kit (Elabscience) was used to determine the level of IL‐33 and ST2 protein in the sera. The experimental procedures were performed according to the manufacturer's instructions.

### 4.9 Gene silencing

Silencing of IL‐33, p16, and p21 in HK‐2 cells was achieved by using a reverse siRNA transfection procedure performed in six‐well plates. Once grown to 70% confluence, cells were transfected with siRNA or scrambled siRNA (RiboBio) using Lipofectamine®RNAiMAX (Invitrogen) according to the manufacturers.

### 4.10 Generation of CM

CM was generated by culturing HK‐2 cells for 14 days and then in serum‐free and drug‐free media for 24 h before harvest, followed by clarification through centrifugation. The resulting CM was mixed with fresh medium in a proportion of 1 to 1 to generate CM containing 10% FBS. Young HK‐2 cells were then treated with different groups of CM for 48 h.

### 4.11 Liquid chromatography–tandem mass spectrometry

The extraction of oxylipins from kidney tissue was performed as described previously with some modifications.^45^ Briefly, frozen tissue was mixed with an antioxidant solution and 5 µL of deuterated internal standards before tissue homogenization and protein precipitation. The ultrasound‐assisted extraction was performed under 4°C for 30 min. Following centrifugation, the supernatant was loaded onto Phenomenex Strata‐X polymeric sorbent columns. After loading the samples, the columns were washed with 90:10 water/methanol to remove impurities, and the oxylipins were then eluted with 1 mL of 100% methanol. The eluant was dried under a nitrogen stream and reconstituted in 100 µL 50:50 water/methanol before liquid chromatography–tandem mass spectrometry (LC–MS) analysis.

LC–MS analyses of oxylipins were performed on a Shimadzu LC‐20AD series HPLC system coupled to an AbSciex 4500 QTRAP mass spectrometer fitted with a TurboV ion source. Oxylipins were separated on a reversed‐phase Zorbax Eclipse Plus C18 column. The solvent system consisted of ACN/water/acetic acid (60:40:0.02, v/v/v) (mobile phase A) and ACN/IPA (50:50, v/v) (mobile phase B) with a flow rate of 0.4 mL/min. The following gradient program was used: 0−4.0 min, 0.1–55% B; 4.0–15.0 min, 55−99% B; 15.0‐20.0 min, 99% B; 20.1–25.0 min, 55–0.1%B. Samples were kept at 4°C and the injection volume was 10 µL. Mass spectrometric analyses were conducted using the following parameters: curtain gas, nebulizer gas, and turbo‐gas (GS2) were set at 30, 40, and 40 psi, respectively. Oxylipins were analyzed using scheduled multiple reaction monitoring in the scanning range of *m*/*z* 50−640 and the scan rate was 1000 µ/s. Data acquisition and analysis were performed using Analyst software, version 1.6.3. Oxylipins were quantified by the stable isotope dilution method. For each class of oxylipins to be quantified, one internal standard was chosen and calibration curves were established.

### 4.12 Acquisition of microarray data

The datasets of transcriptome profiling by microarray were searched on GEO profiles (https://www.ncbi.nlm.nih.gov/geoprofiles/) with the keywords “diabetic kidney disease” or “diabetic nephropathy.” All microarray datasets with IL‐33 were downloaded for analyses, including GSE96804, GSE33744, GSE118089, GSE51205, GSE142025, GSE20636, GSE30528, GSE107942, GSE30529, GSE86300, GSE87359, GSE90842, GSE134327, and GSE131266.

### 4.13 Study population

Our study was based on the data from the UK Biobank, a large‐scale prospective cohort study.^46^ The cohort`’s procedures and baseline characteristics are available on the UK Biobank website (www.ukbiobank.ac.uk). The definition was described according to the 9th and 10th revisions of the International Classification of Diseases (ICD‐9 and ICD‐10) and self‐reported data fielded with the choice‐, disease‐, or procedure‐specific codes. Excluding individuals without relevant SNPs data, we eventually included 6693 DN and 463,084 non‐DN participants.

### 4.14 PRS calculation

SNPs within the* IL33* (Chromosome 9, NC_000009.12 (6215149..6257983)) and nearby region (±500 kb) were downloaded from UK Biobank. Detailed information about genotyping, imputation, and quality control has been described elsewhere.^47^, ^48^ After excluding minor allele frequency < 0.05, 2684 SNPs were obtained by using PLINK2. Then, eQTL analyzed the association between IL‐33 mRNA expression in whole‐blood and SNPs using the GTEx platform (https://www.gtexportal.org/home/). Among the SNPs with *p *< 0.01, SNPs negatively correlated with the expression of IL‐33 were excluded, and seven relatively independent SNPs (LD < 0.8) were eventually included in PRS. The PRS was depicted in previous studies^46^, ^49^ and was categorized as low (lowest tertile), intermediate (second tertile), and high (highest tertile) genetic risk (tertiles were based on the distribution of the PRS). The equation was:

$$\mathrm{PRS}=\left(\beta 1*\mathrm{SNP}1+\beta 2*\mathrm{SNP}2+\cdots +\beta n*\mathrm{SNP}n\right)$$

where *β* was the regression coefficient per allele with IL‐33‐expression‐related SNPs. SNPn was recorded as 0, 1, or 2 in terms of the number of risk alleles, and *n* was 7, namely the total SNPs number in this study.

### 4.15 Data analysis

The population data were analyzed using SAS (version 9.4) or R (version 4.2.2). The experiment data were analyzed using Graph Pad Prism 8.3 and showed as the mean ± SEM. Differences among the groups were determined by the Student's t‐test or one‐way analysis of variance. Differences were identified as statistically significant at 3 levels: *p *< 0.05, *p* < 0.01, and *p *< 0.001.

## AUTHOR CONTRIBUTIONS

Li Chen, Xingzhu Yin, Li Mo, Chunjie Jiang, Ping Yao, Chao Gao, and Yuhan Tang designed the research study. Qianchun Deng, Liangkai Chen, Jiansha Li, Hongxia Li, Yanyan Li, and Yuhan Tang contributed to the collection, analysis, and interpretation of data. Xueer Cheng, Huimin Chen, Bangfu Wu, and Ying Zhao conducted laboratory experiments. All authors have read and approved the final manuscript.

## CONFLICT OF INTEREST STATEMENT

All authors declared no conflict of interest.

## ETHICS STATEMENT

All experiments involving animals were conducted according to the ethical policies and procedures approved by the ethics committee of the Tongji Medical College Council on Animals Care Committee (Approval no. S820). All experiments involving humans were conducted according to the ethical policies and procedures approved by the Medical Ethics Committee, Tongji Medical College, Huazhong University of Science and Technology (Approval no. S149). Informed consent was obtained from all participants in the study.

## Supporting information



Supporting Information

## Data Availability

The data, analytical methods, and study materials will be made available to other researchers for purposes of replicating the procedure and are available by contacting the corresponding authors. Oxylipin data were uploaded to EBI‐MetaboLights (MTBLS10751).^50^
