# Supplementary material for: Partial reduction of interleukin‐33 signaling improves senescence and renal injury in diabetic nephropathy
Source: MedComm (2020). 2024 Oct 24;5(11):e742. doi: 10.1002/mco2.742 (PMC11502718; doi:10.1002/mco2.742)
Supplement: Supplementary file 1 — Supporting Information [file MCO2-5-e742-s001.docx]

**Supplementary information**

**Partial reduction of interleukin-33 signaling improves senescence and renal injury in diabetic nephropathy**

**Running title:** Interleukin-33 **and diabetic nephropathy**

Li Chen^1,2,^^#^, Chao Gao^3,#^, Xingzhu Yin^1,#^, Li Mo^1^, Xueer Cheng^1^, Huimin Chen^1^, Chunjie Jiang^1^, Bangfu Wu^1^, Ying Zhao^1^, Hongxia Li^1^, Yanyan Li^4^, Jiansha Li^5^, Liangkai Chen^1^, Qianchun Deng^2^, Ping Yao^1^, and Yuhan Tang^1,*^

^1^Department of Nutrition and Food Hygiene, Hubei Key Laboratory of Food Nutrition and Safety, Ministry of Education Key Laboratory of Environment and Health and MOE Key Lab of Environment and Health, Key Laboratory of Environment and Health (Wuhan), Ministry of Environmental Protection, State Key Laboratory of Environment Health (Incubation), School of Public Health, Tongji Medical College, Huazhong University of Science and Technology, Wuhan 430030, China

^2^Oil Crops Research Institute of the Chinese Academy of Agricultural Sciences, Hubei Key Laboratory of Lipid Chemistry and Nutrition, and Key Laboratory of Oilseeds Processing, Ministry of Agriculture, Oil Crops and Lipids Process Technology National & Local Joint Engineering Laboratory, Wuhan 430062, Hubei, China

^3^National Institute for Nutrition and Health, Chinese Center for Disease Control and Prevention Beijing, China

^4^Shenzhen Center for Chronic Disease Control, 2021 Buxin Road, Shenzhen, China

^5^Institute of Pathology, Tongji Hospital, Wuhan, China, Department of Pathology, School of Basic Medicine, Tongji Medical College, Huazhong University of Science and Technology, Wuhan, China

^#^Li Chen, Chao Gao, Xingzhu Yin contributed equally to this work.

**^*^Correspondence author**

Yuhan Tang; Email: [2015220157@hust.edu.cn;](mailto:2015220157@hust.edu.cn;) Tel: +86-27-8365-0522; Fax: +86-27-8365-0522

**This file includes:** Figure S1-7, Table S1-3.

**Supplementary Figure S1**

**A**


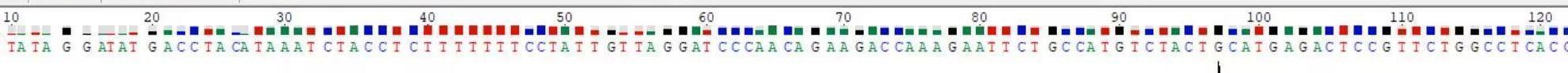


**IL-33^+/+^**


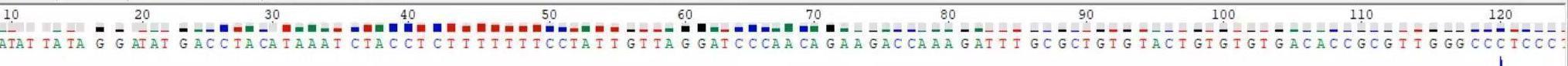


**IL-33^+/-^**


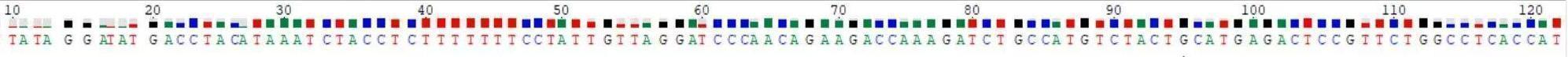


**IL-33^-/-^**

**B**


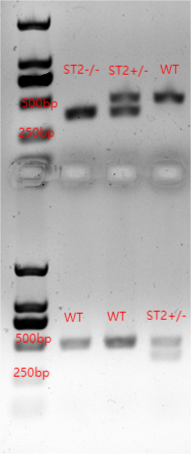


**C**


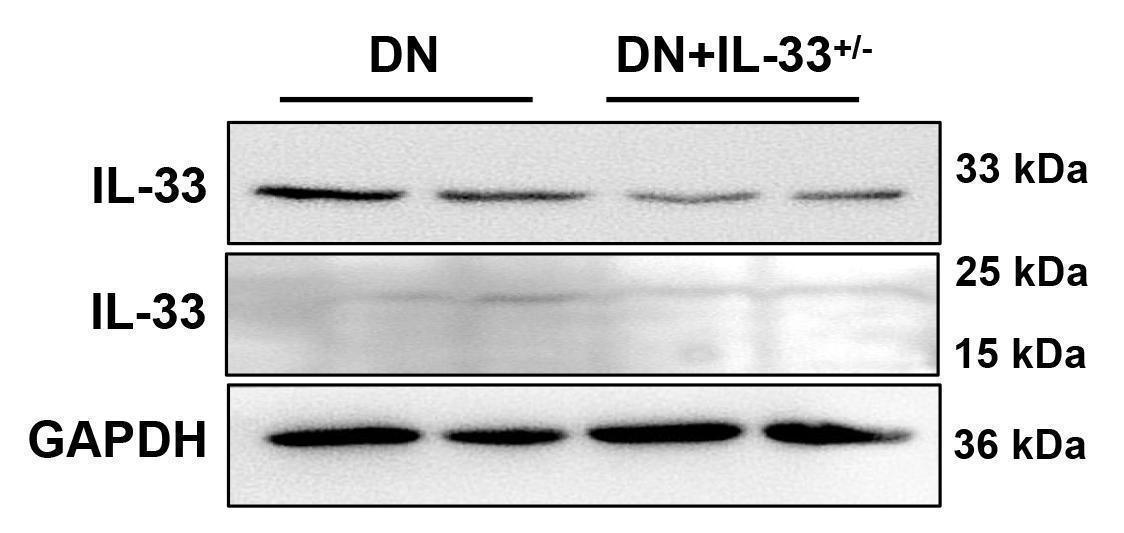


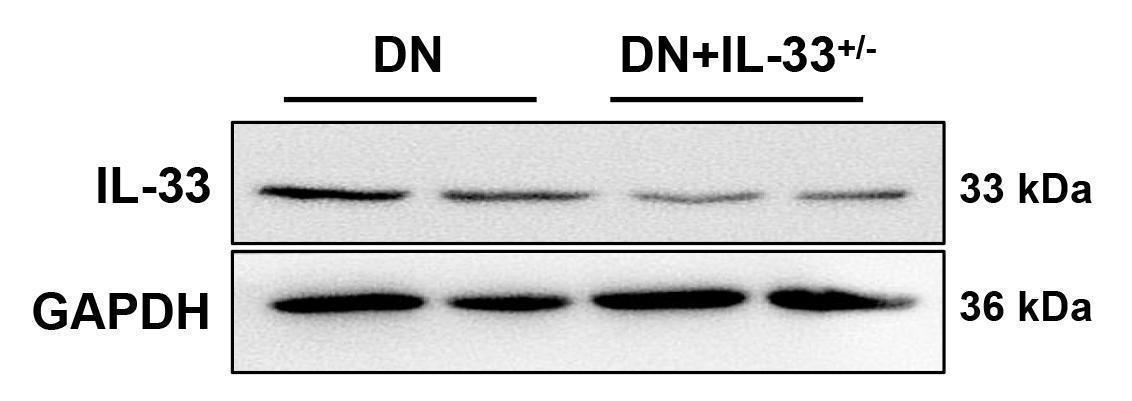


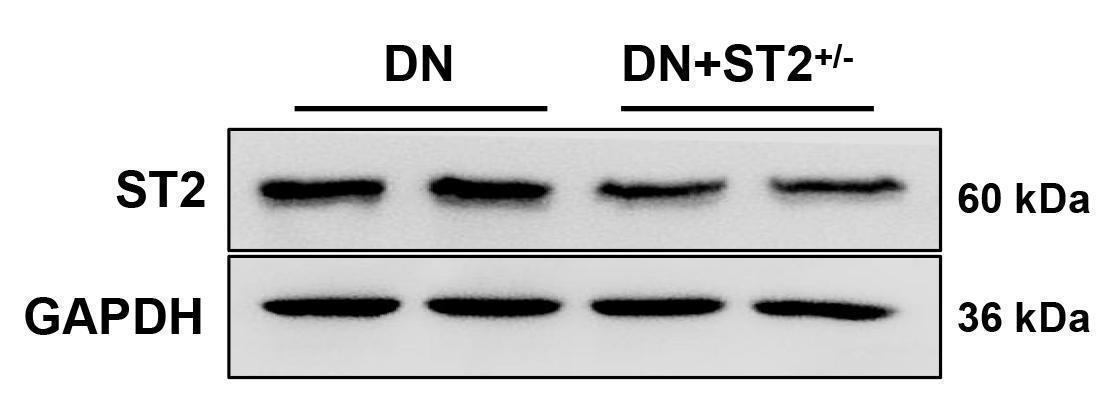


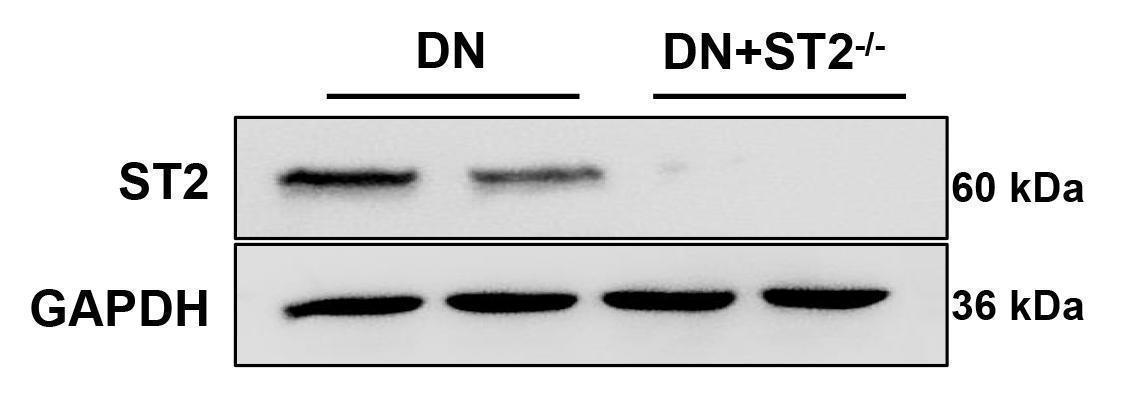


Figure S1. Validation of IL-33 and ST2 knockout mice. (A and B) Mouse tails were used for genotyping analysis. (C) The expression levels of IL-33 and ST2 proteins in renal tissue at the end of experiment.

**Supplementary Figure S2**


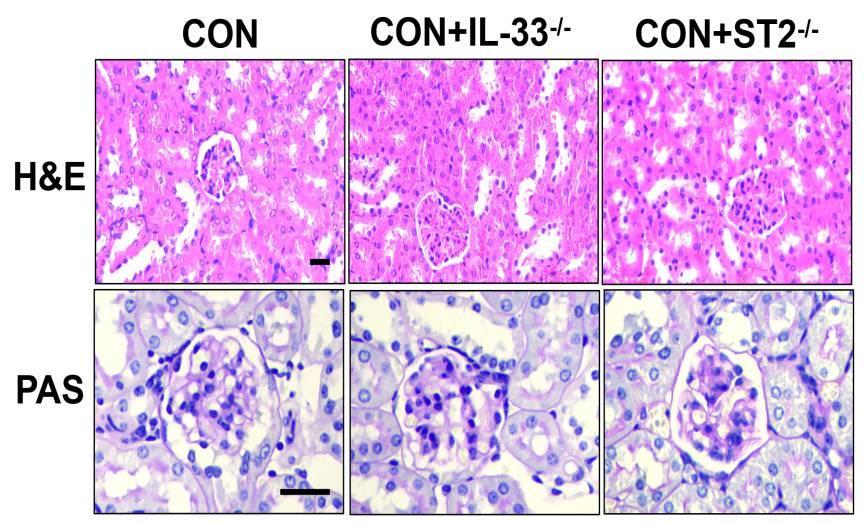

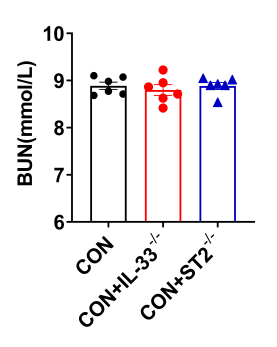

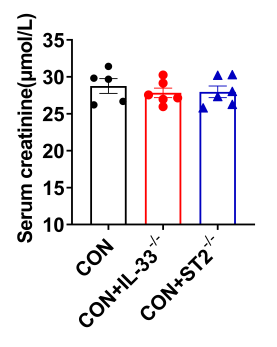

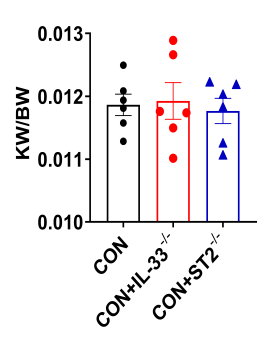

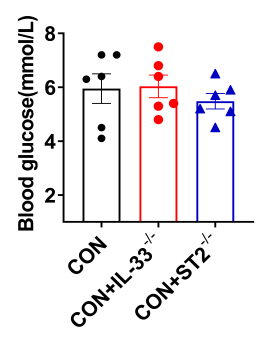

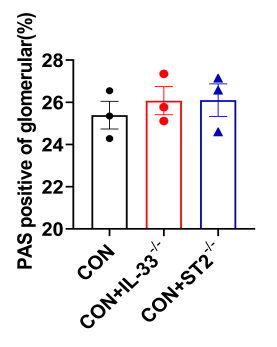

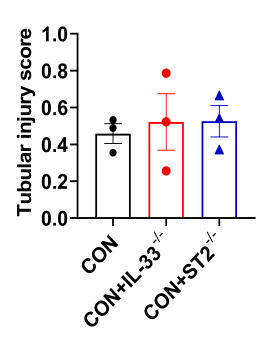

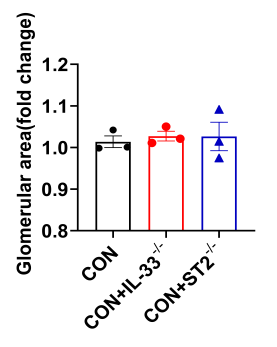


**D**

**C**

**B**

**H**

**A**

**G**

**F**

**E**

Figure S2. Renal injury-related markers in CON, CON+IL-33^-/-^, and CON+ST2^-/-^ mice. (A-D) Fasting blood glucose (FBG), renal-body ratio (KW/BW), serum creatinine (Scr), blood urea nitrogen (BUN) were monitored at the end of the experiment. (E) Representative images of kidney tissue stained with H&E and periodic acid-Schiff (PAS) (scale bar: 20 μm). (F and G) The glomerular area and PAS positive area were assessed in 3 mice with at least 10 glomeruli per mouse. (H) Renal tubular injury score was estimated by H&E staining.

**Supplementary Figure S3**

**A**


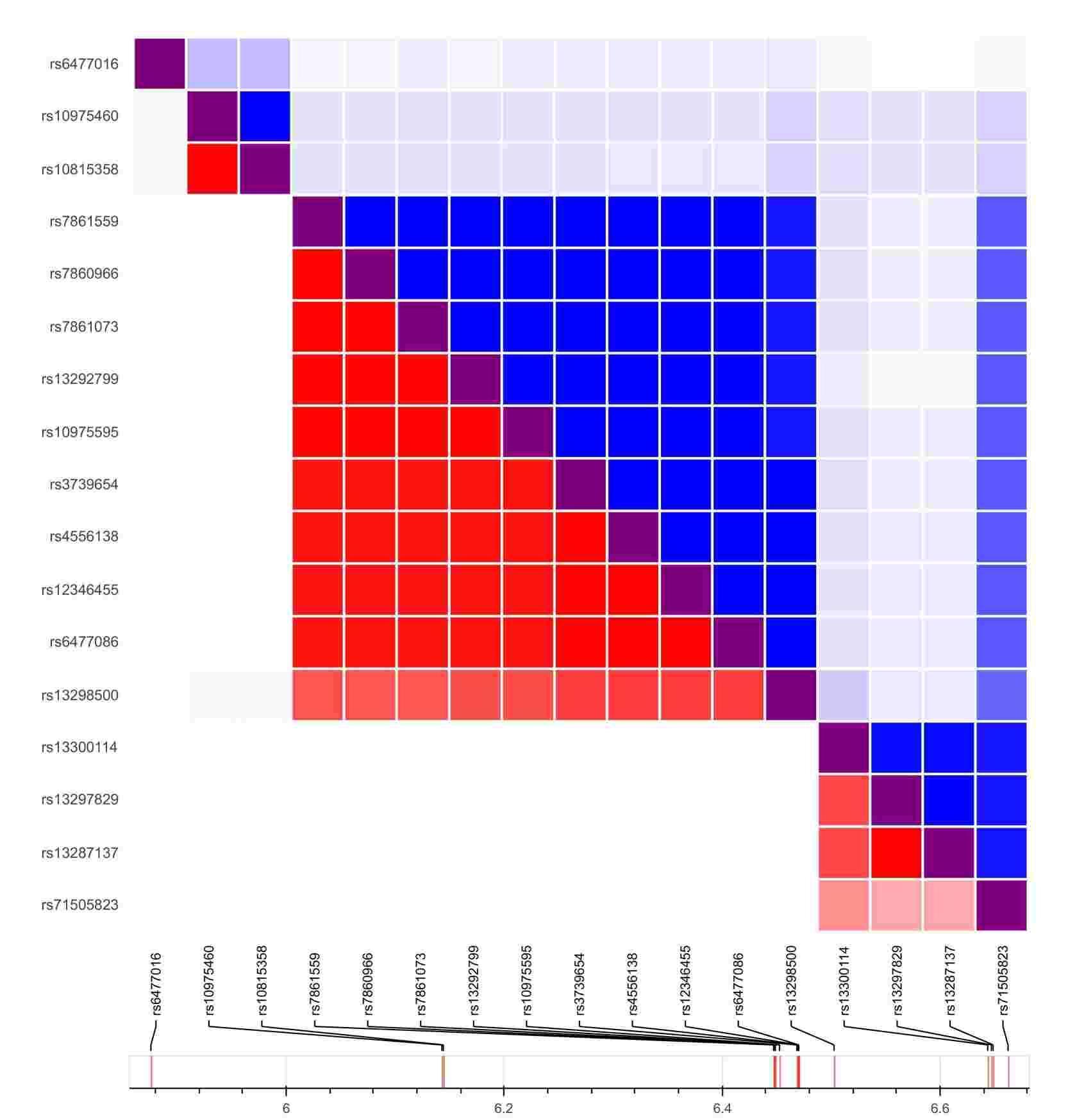


**B**


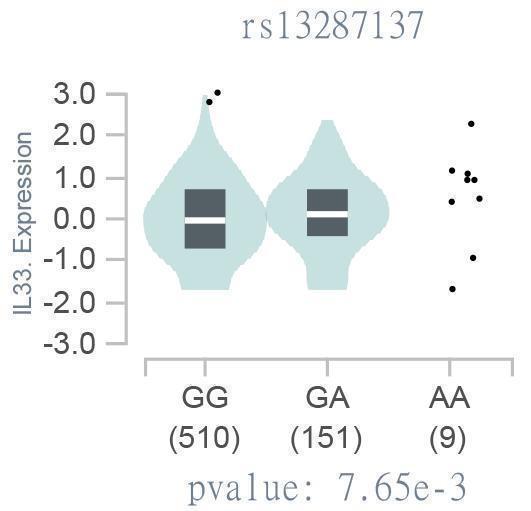


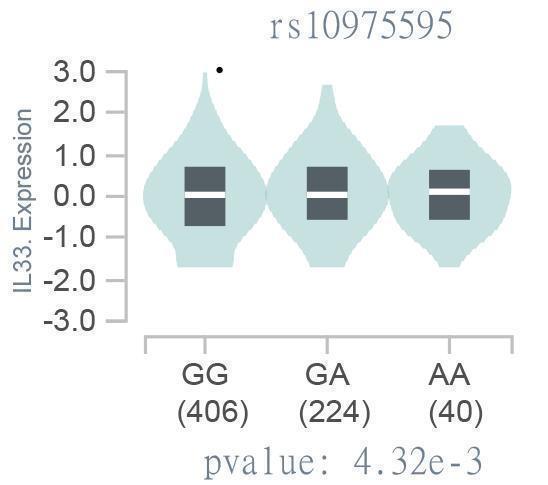

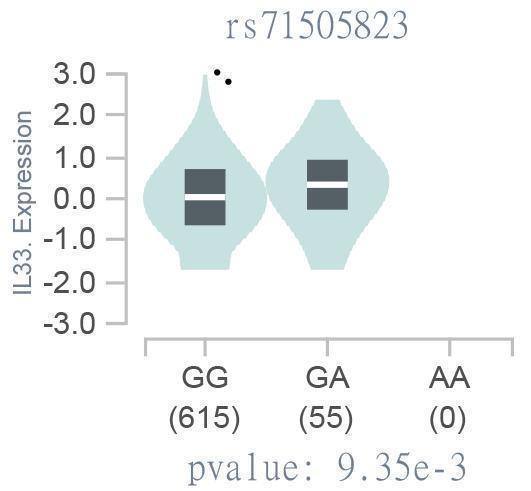


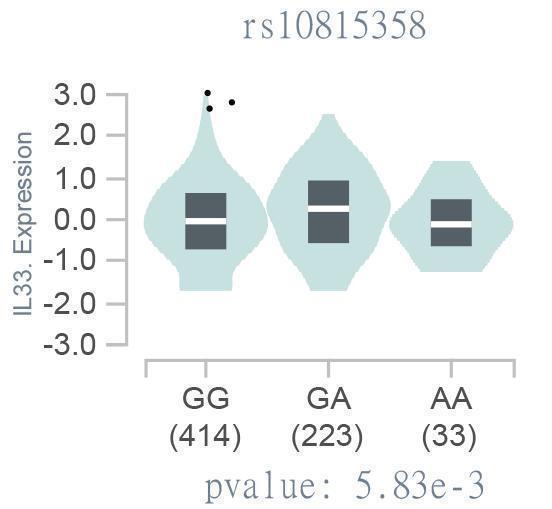

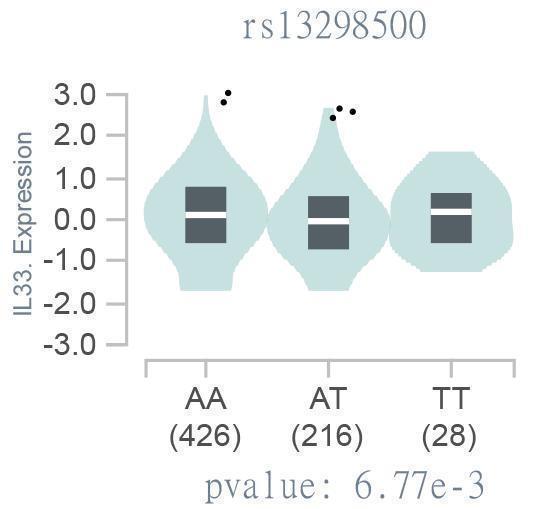

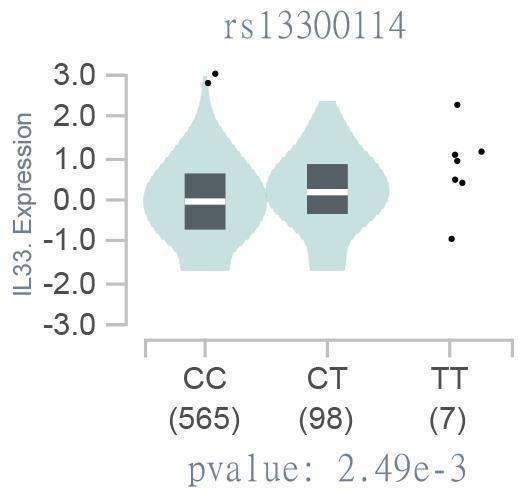

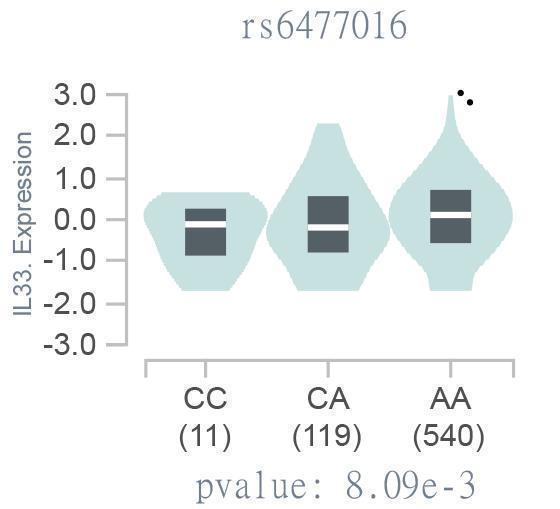


**C**

**D**


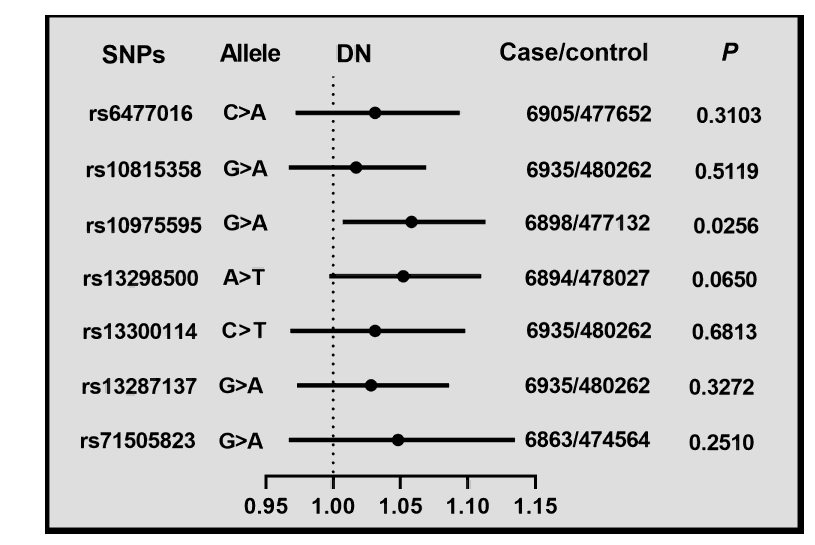


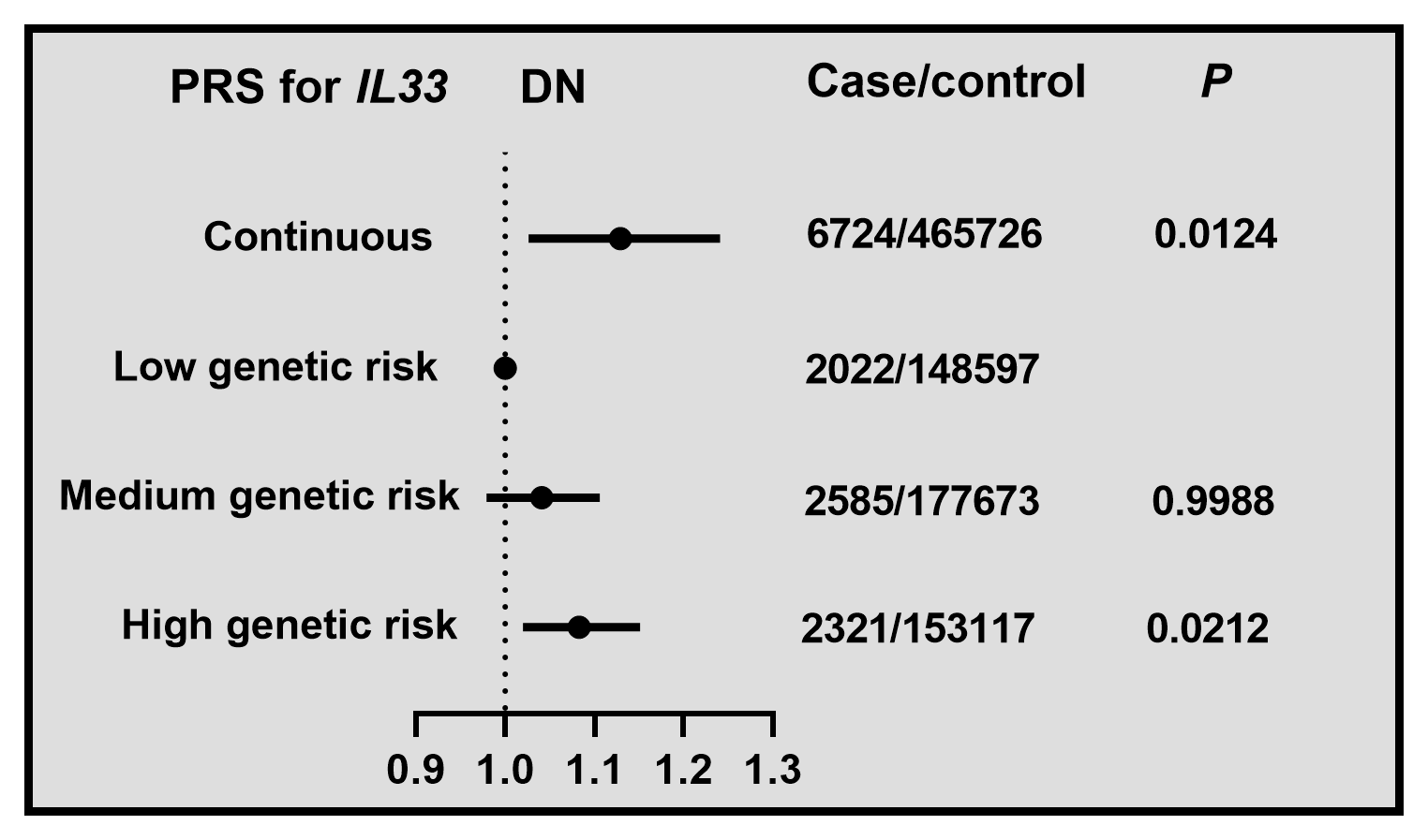


Figure S3. Population-based analysis of IL-33 and DN risk. (A) Relationship matrix of linkage disequilibrium among 17 single nucleotide polymorphisms (SNPs) positively associated with IL-33 expression. (B) Relationship between 7 SNPs and IL-33 expression. (C) Association between each SNPs included in polygenic risk score and the risk of DN. (D) Association of polygenic risk score (PRS) with SNPs which may be associated with DN risk.

**Supplementary Figure S4**

**A**

**B**


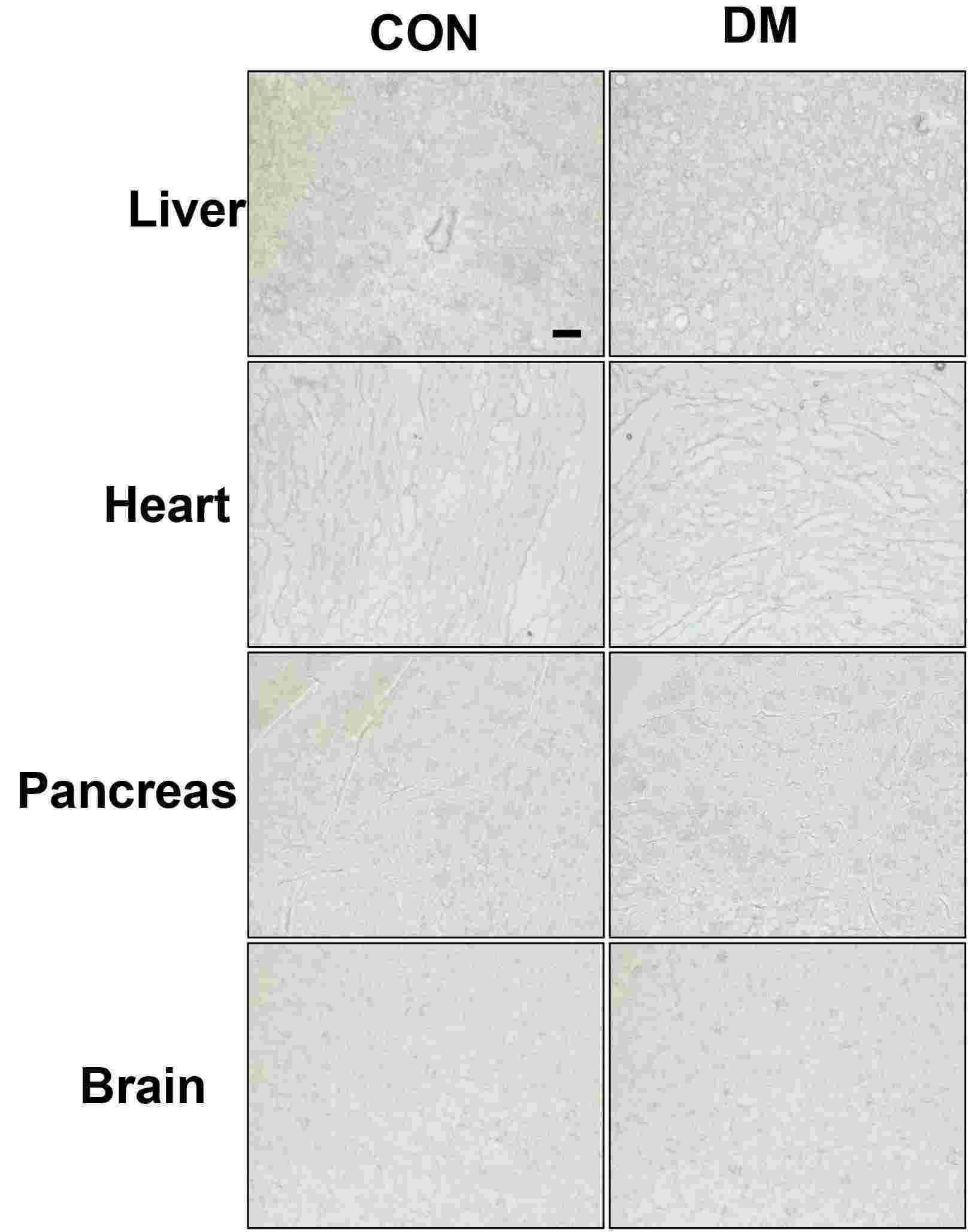


**C**


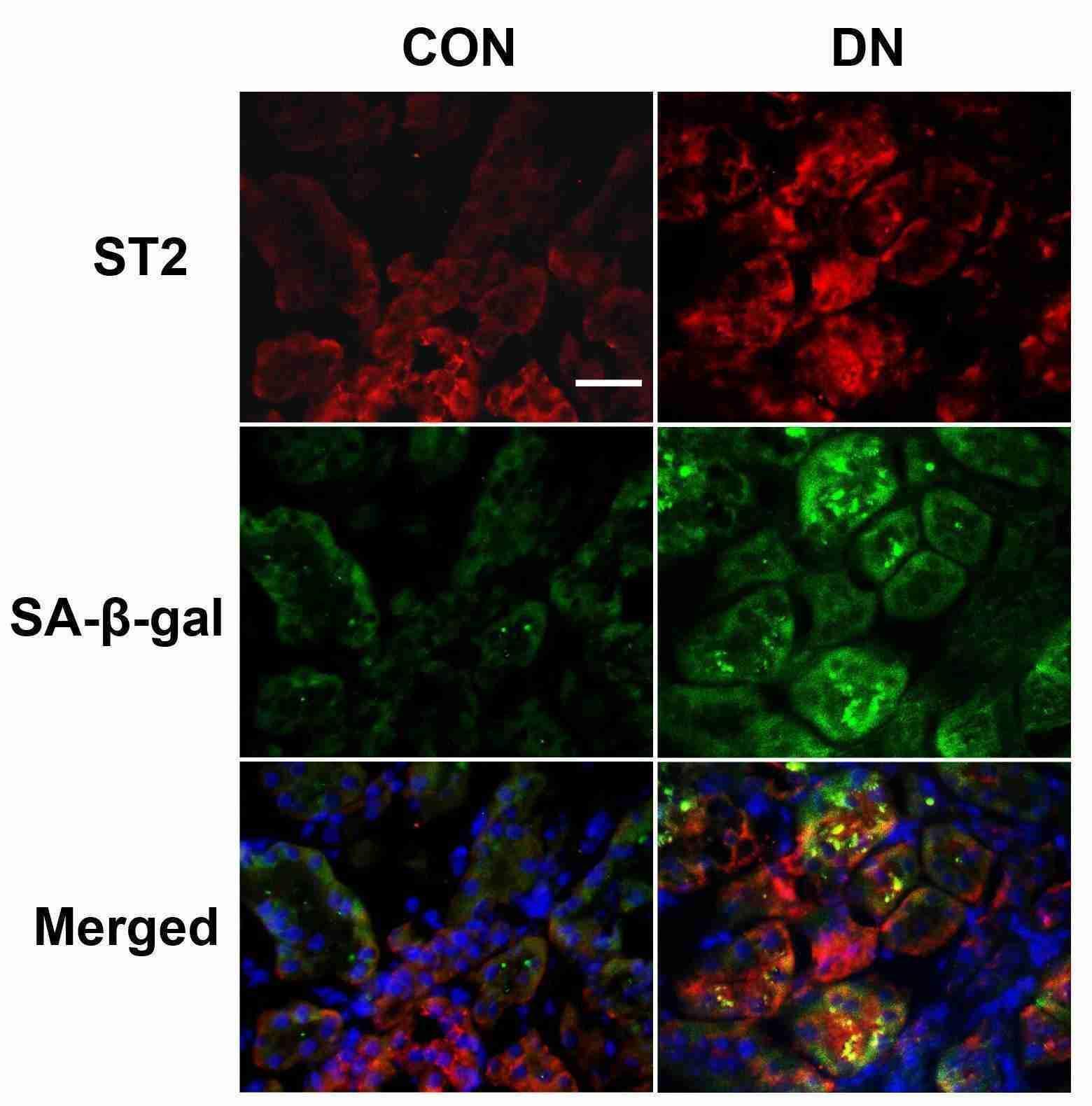


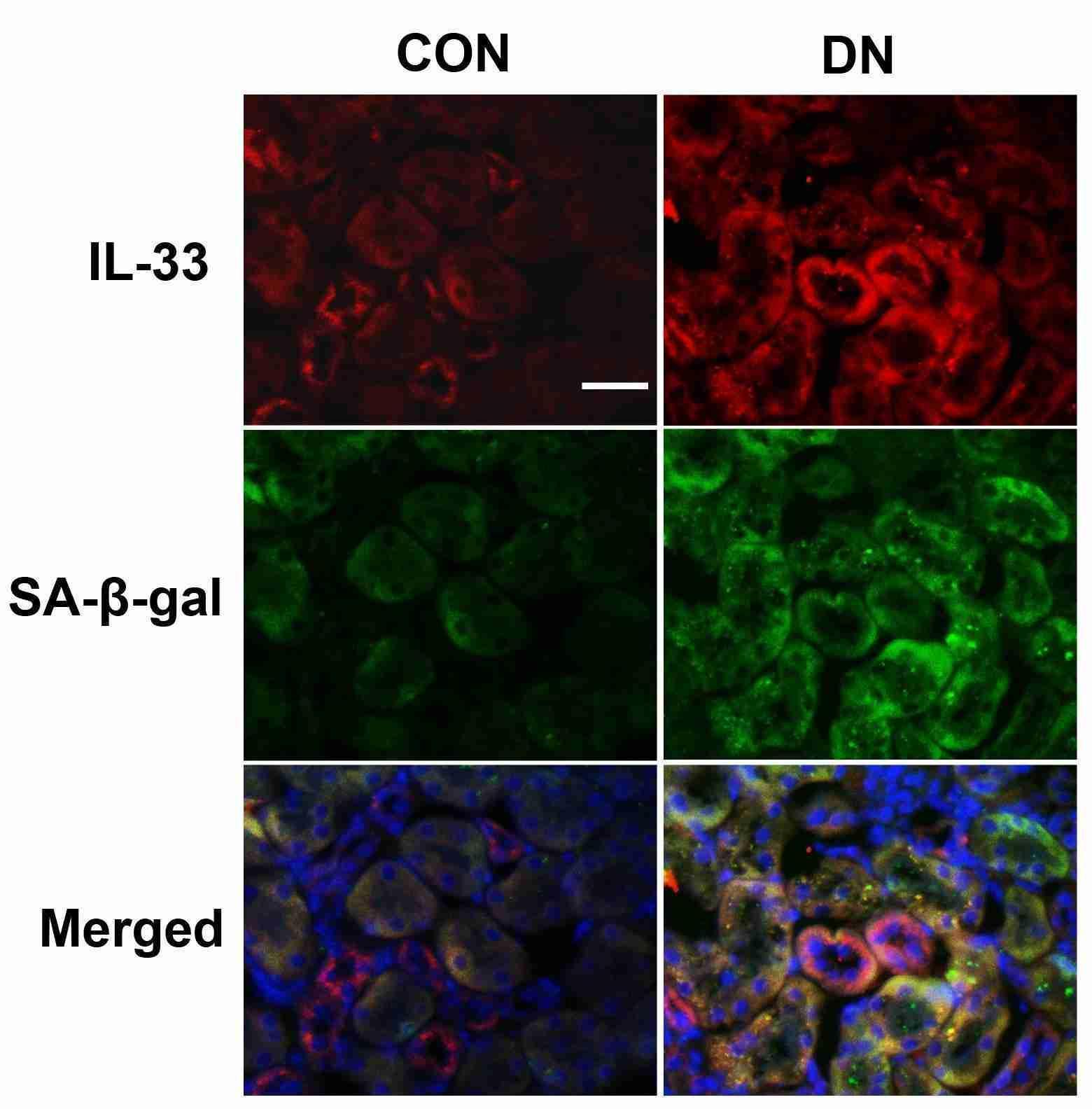


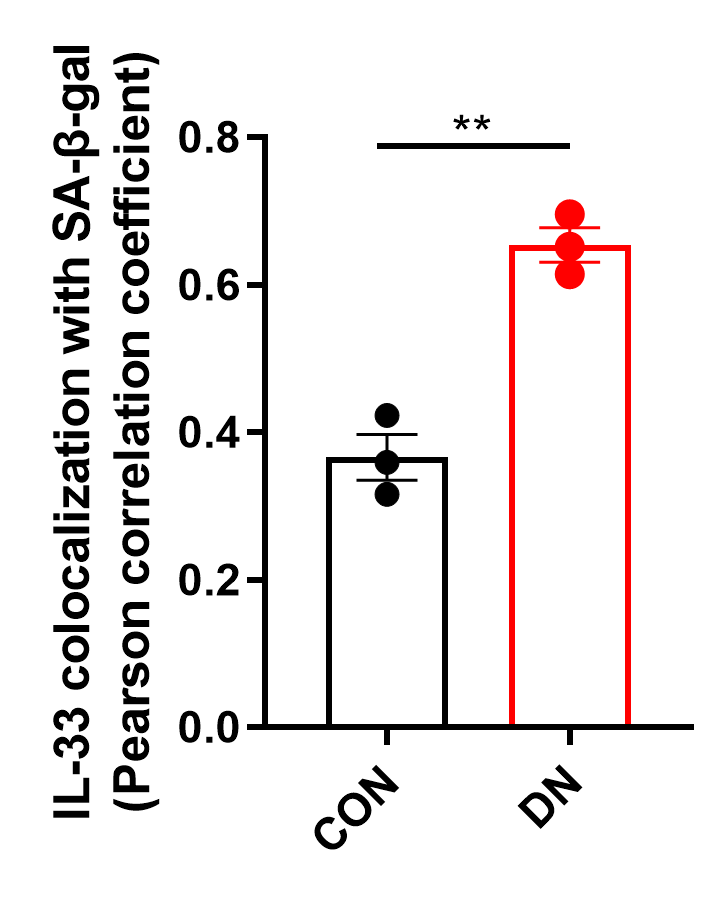


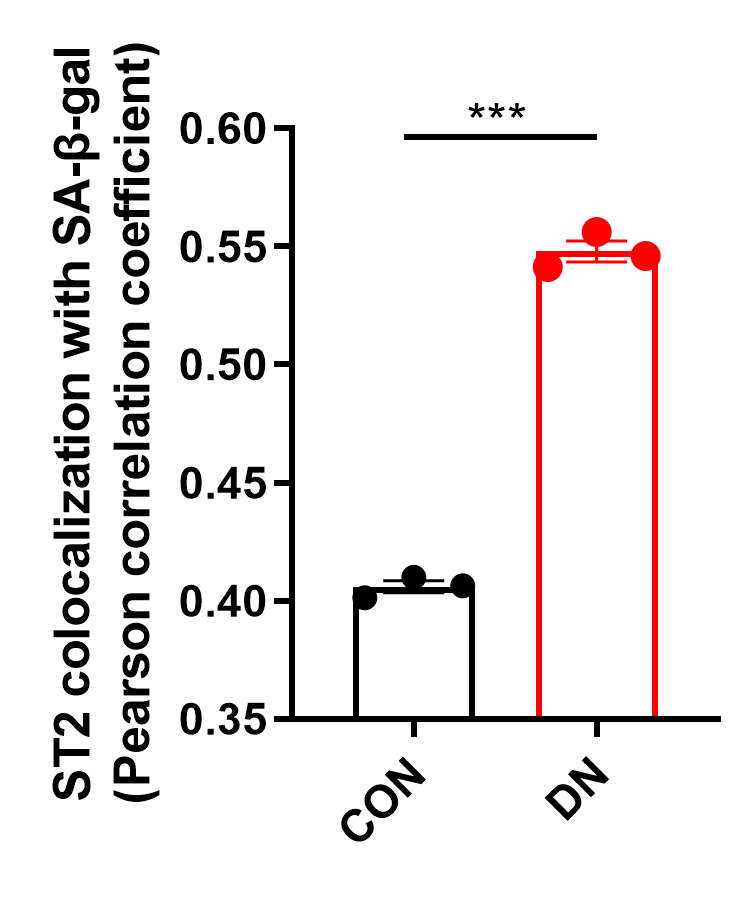


**E**

**D**

**
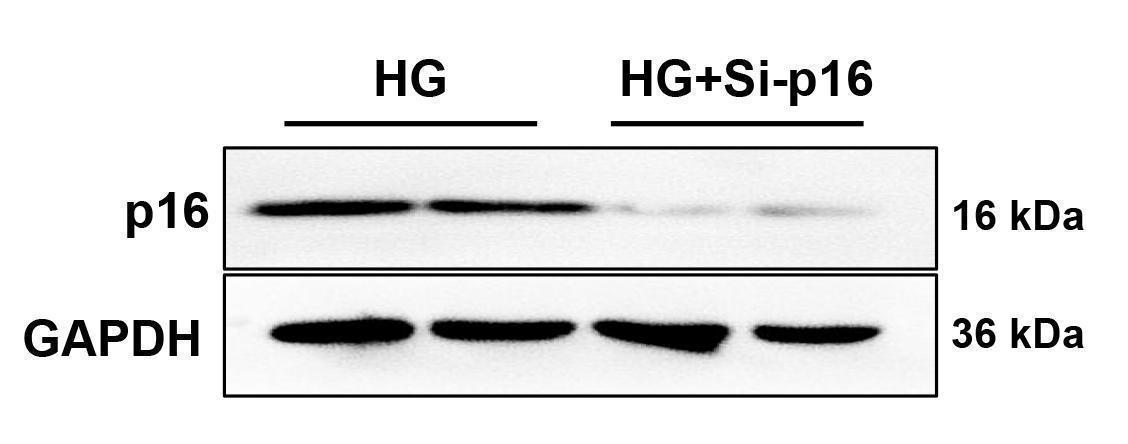
**
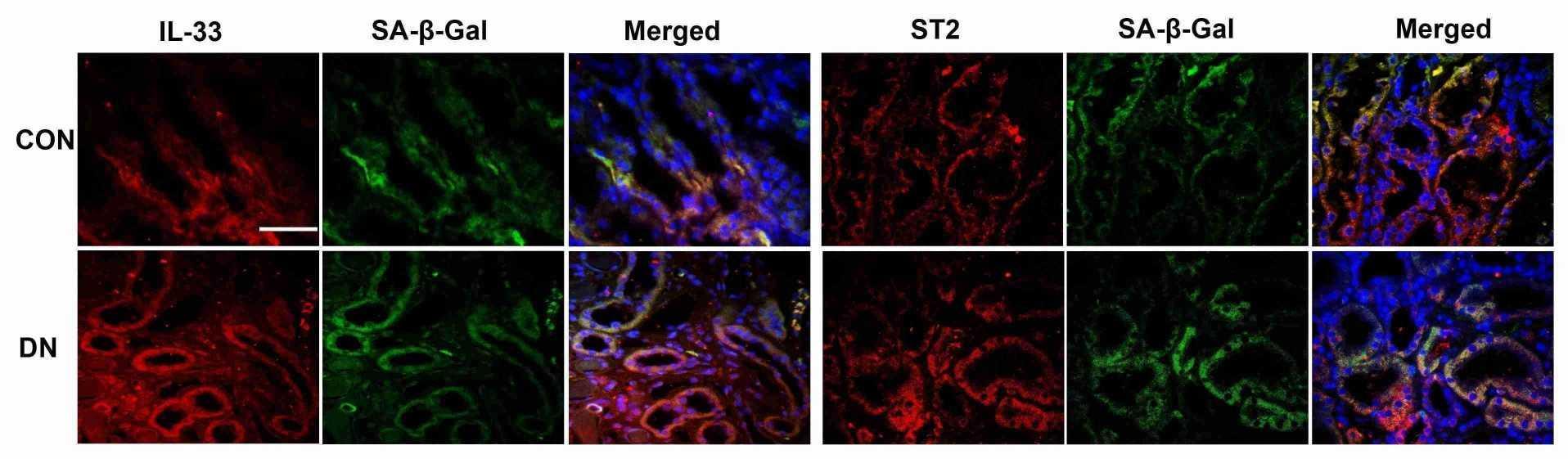


**
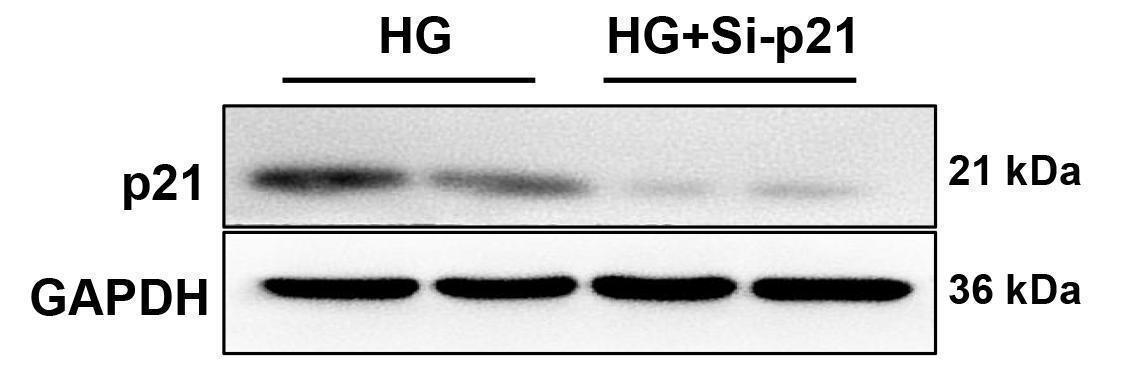
**

**F**

**
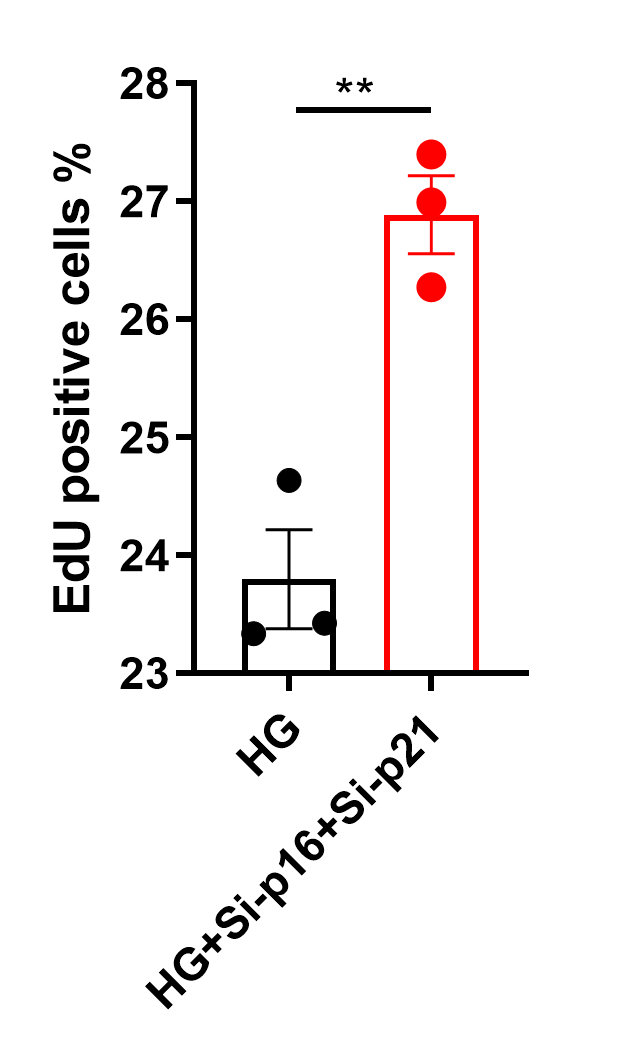

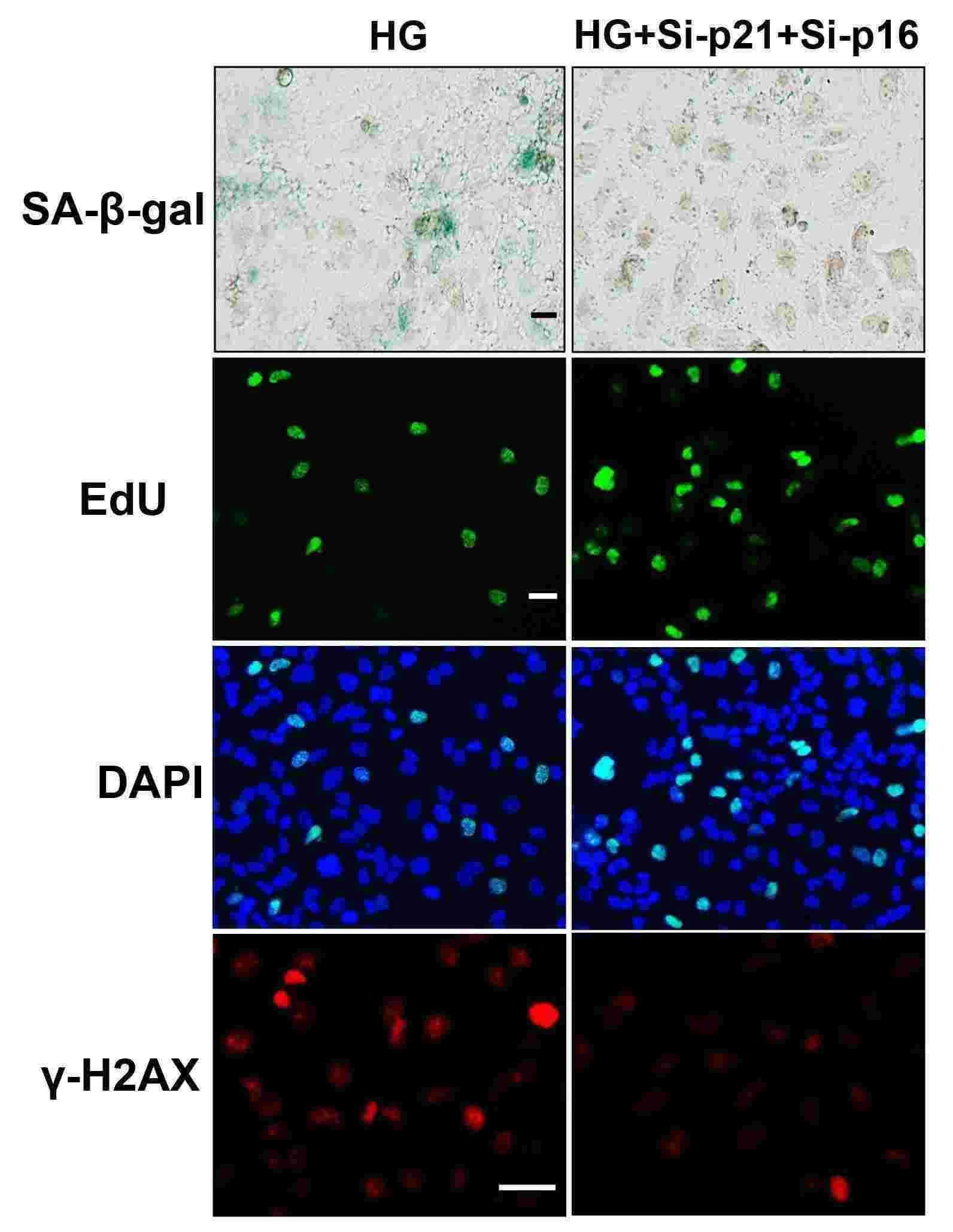
**

**G**

**
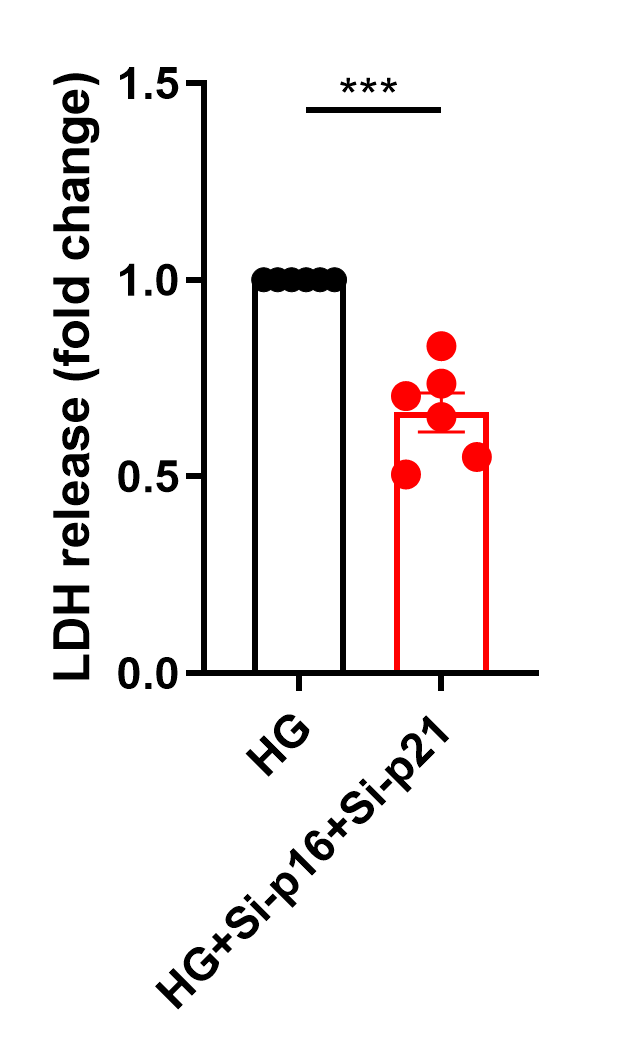

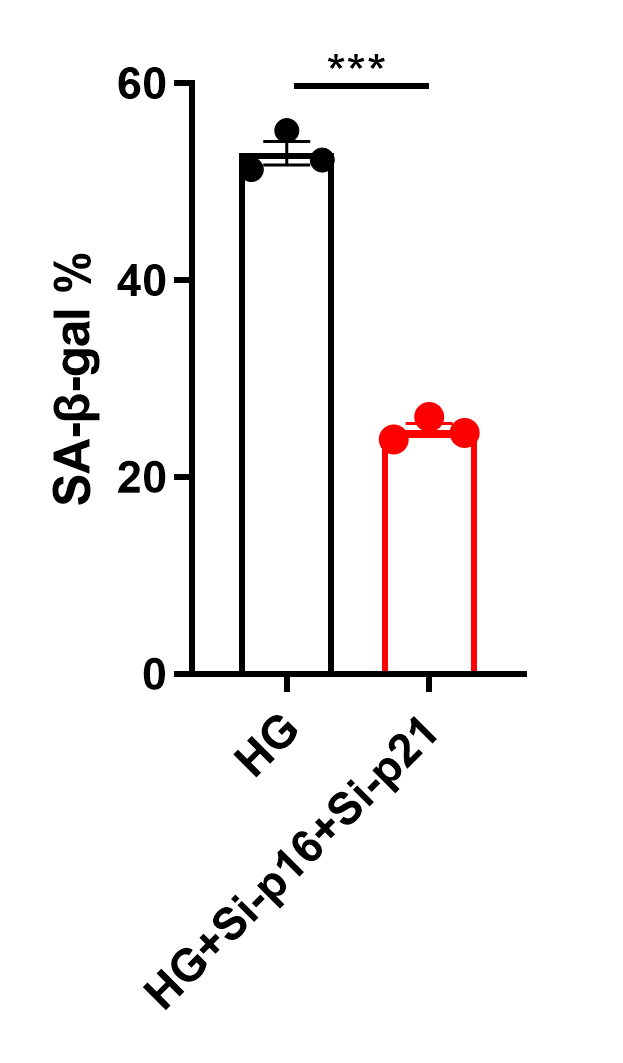

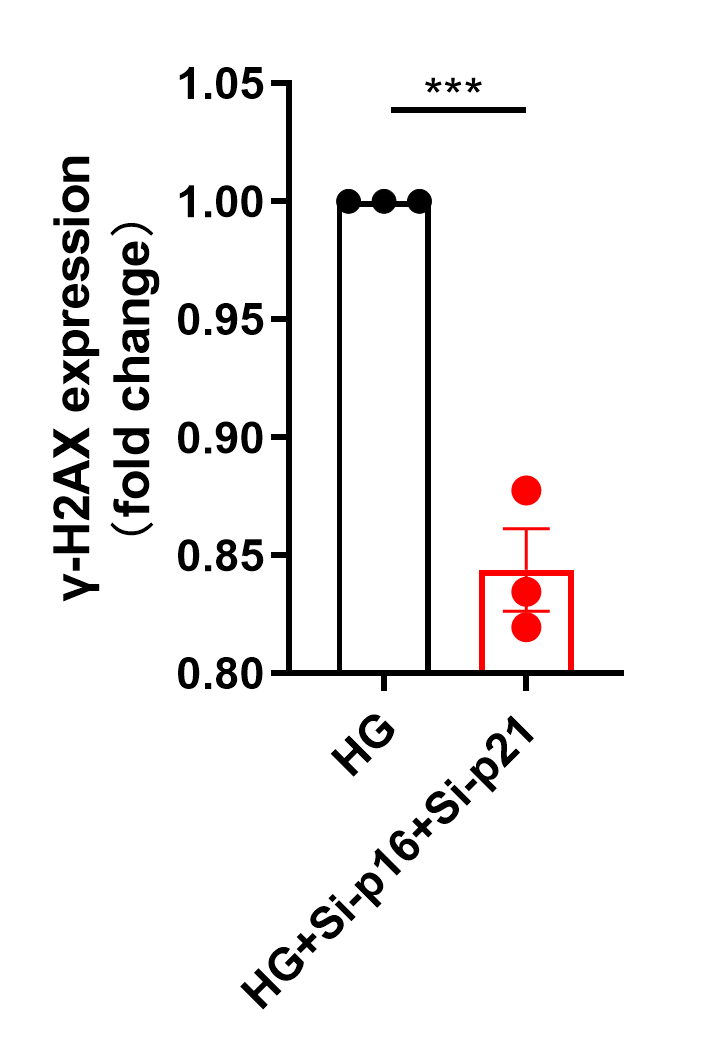

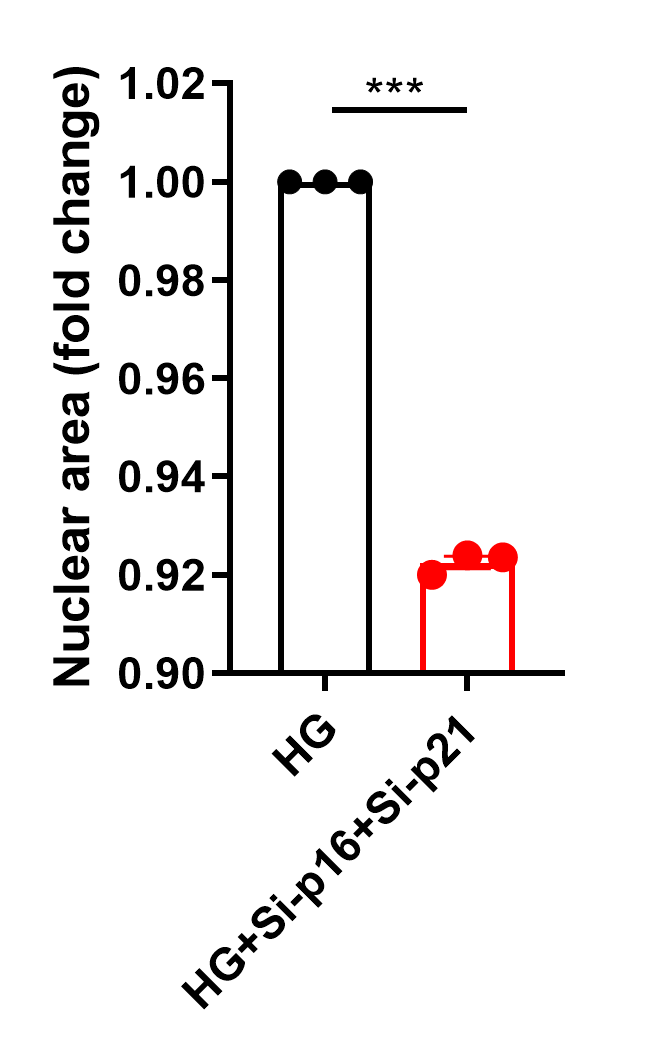
**

**H**

**
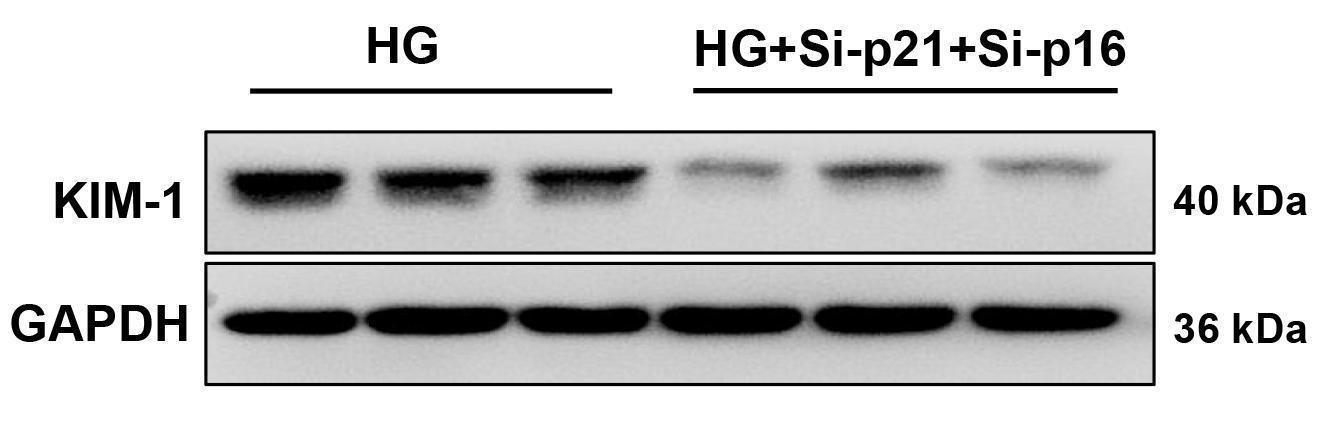
**

Figure S4. The relationship between cell senescence and DN. (A) SA-β-gal staining in frozen sections of liver, heart, pancreas, and brain in mice induced by high-fat diet (HFD) and STZ. (B) Correlation of glomerular filtration rate (eGFR), glomerular area, and KIM-1 expression with p16 expression. (C and D) Colocalization analysis of IL-33 (red) and ST2 (red) with SA-β-gal (green) in DN mice or patients. The positive colocalization area was mainly concentrated in the renal tubules. (E) Immunoblots of p16 and p21 in HK-2 cells with p16 or p21 silencing. (F) Silencing p16 and p21 simultaneously reduced cell senescence (SA-β-gal, EdU, nuclear area, and γH2AX). (G and H) KIM-1 expression and LDH release in HK-2 cells after silencing p16 and p21 simultaneously. Scale bar: 20 μm. **P* < 0.05, ***P* < 0.01, ****P* < 0.001.

**Supplementary Figure S5**

**C**

**B**


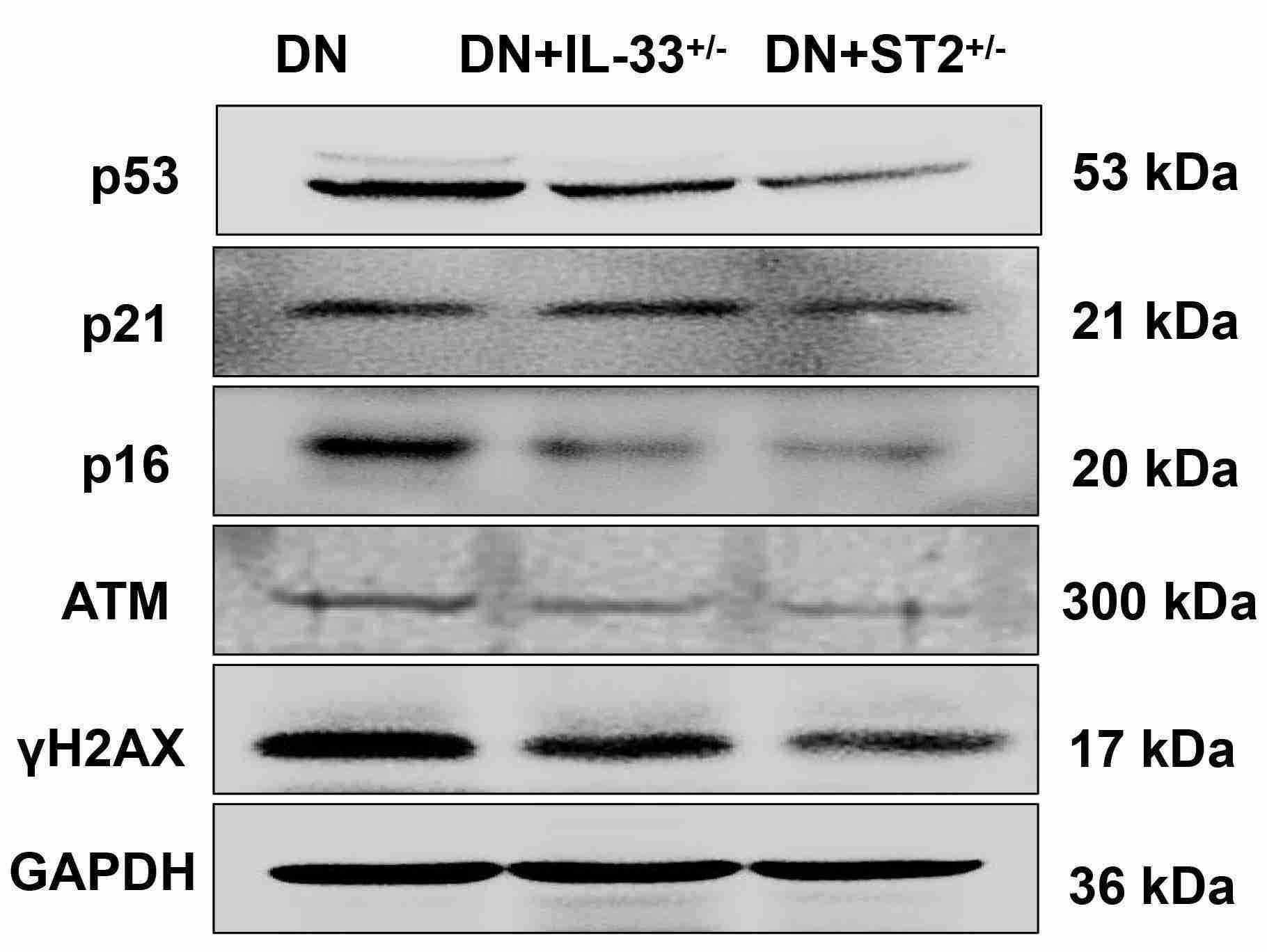

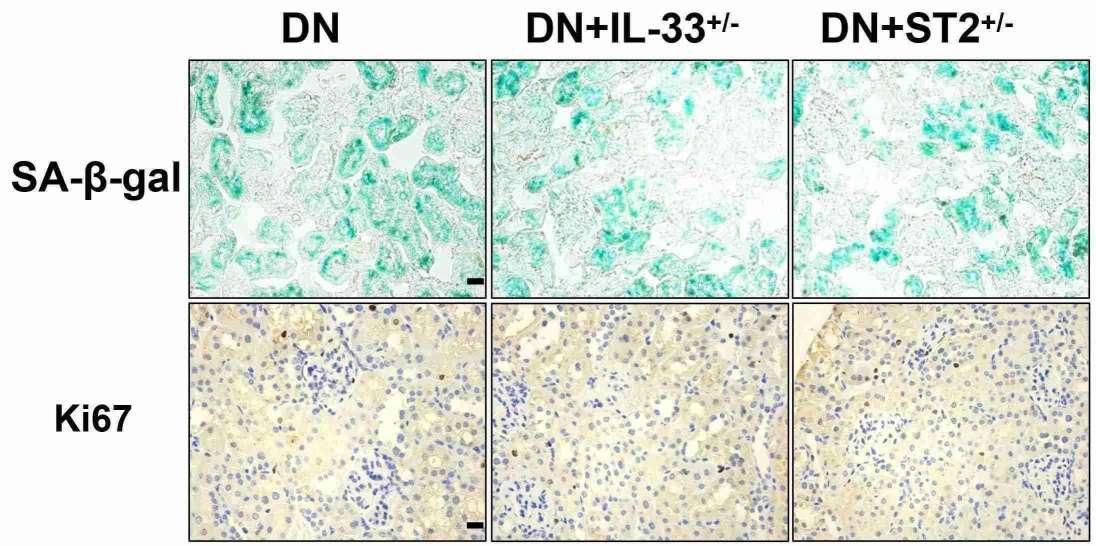


**A**

**H**

**F**

**D**

**Ee**


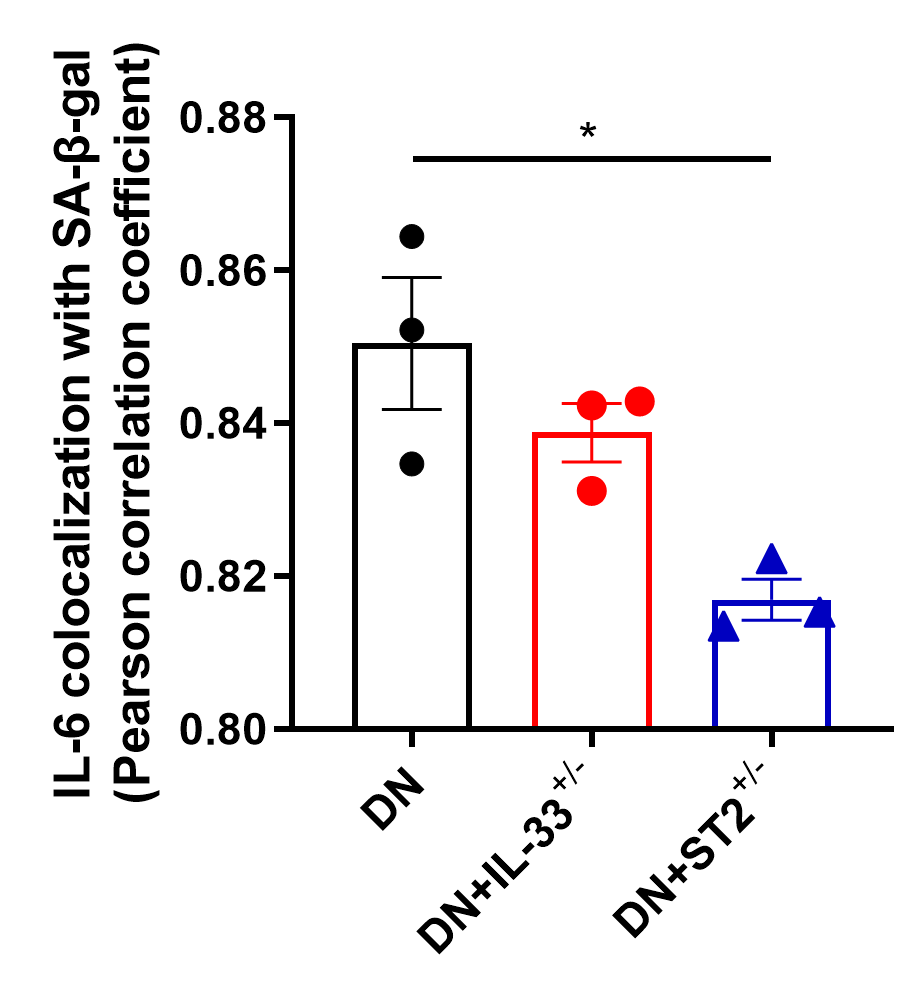

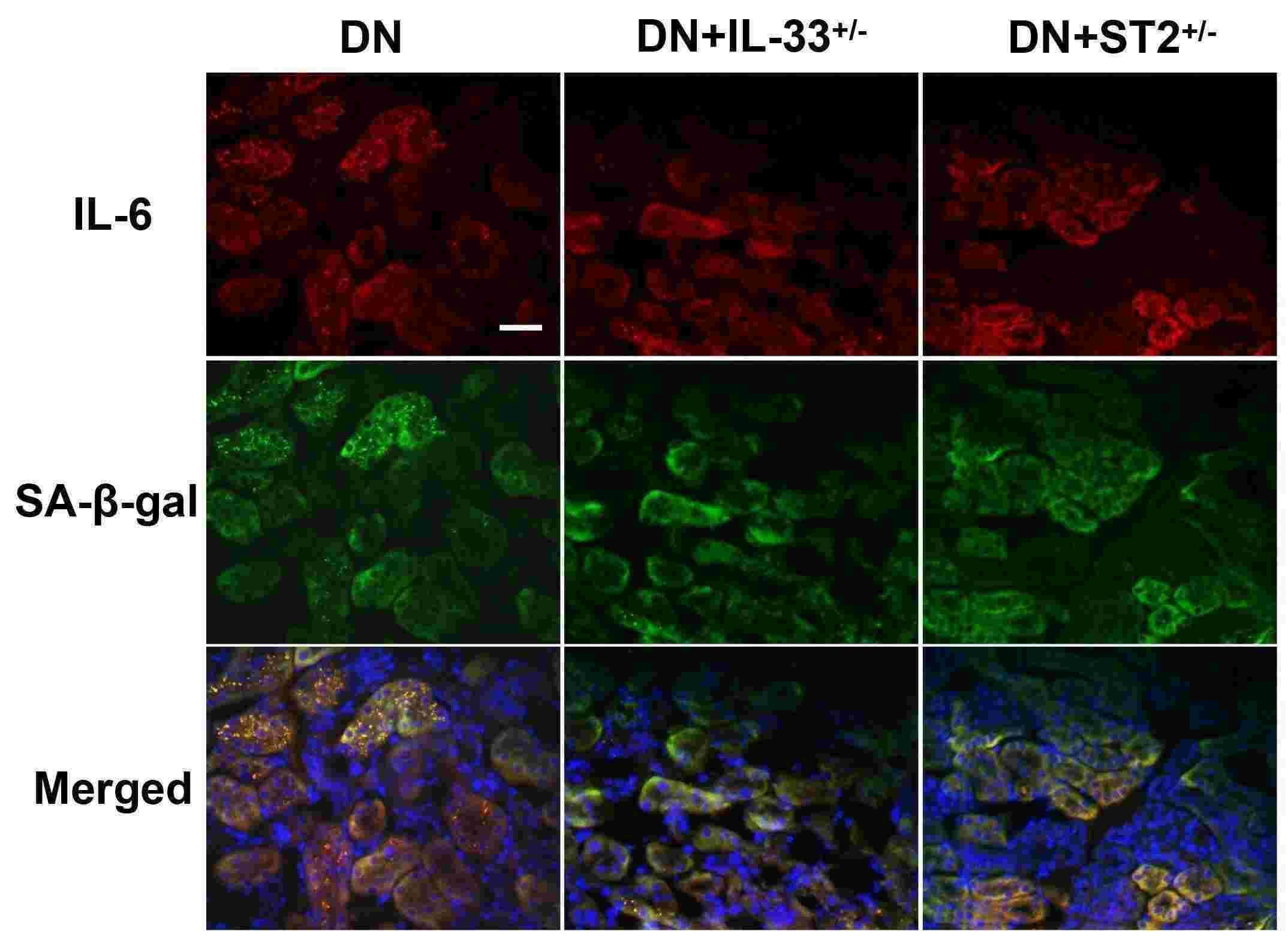

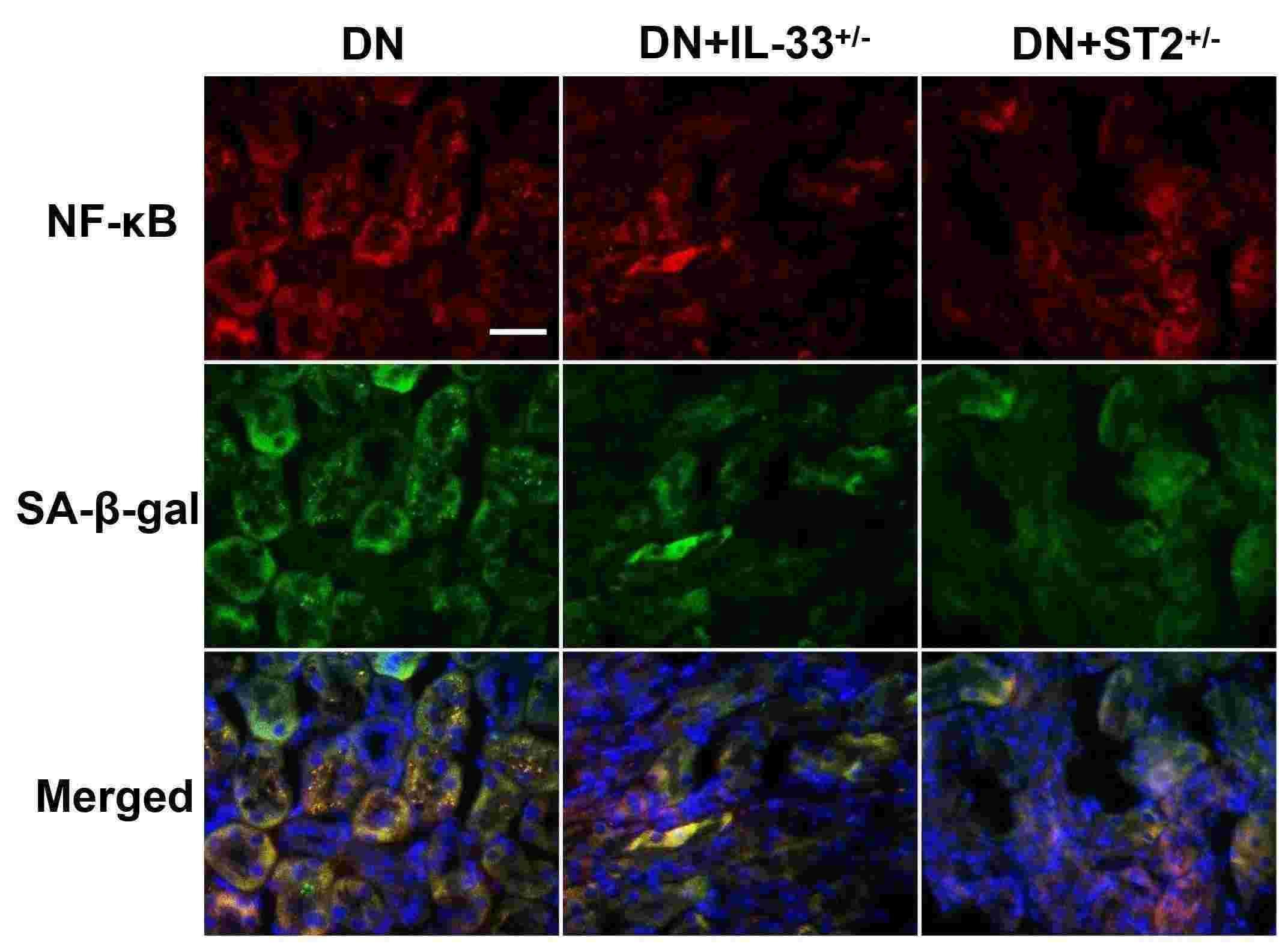


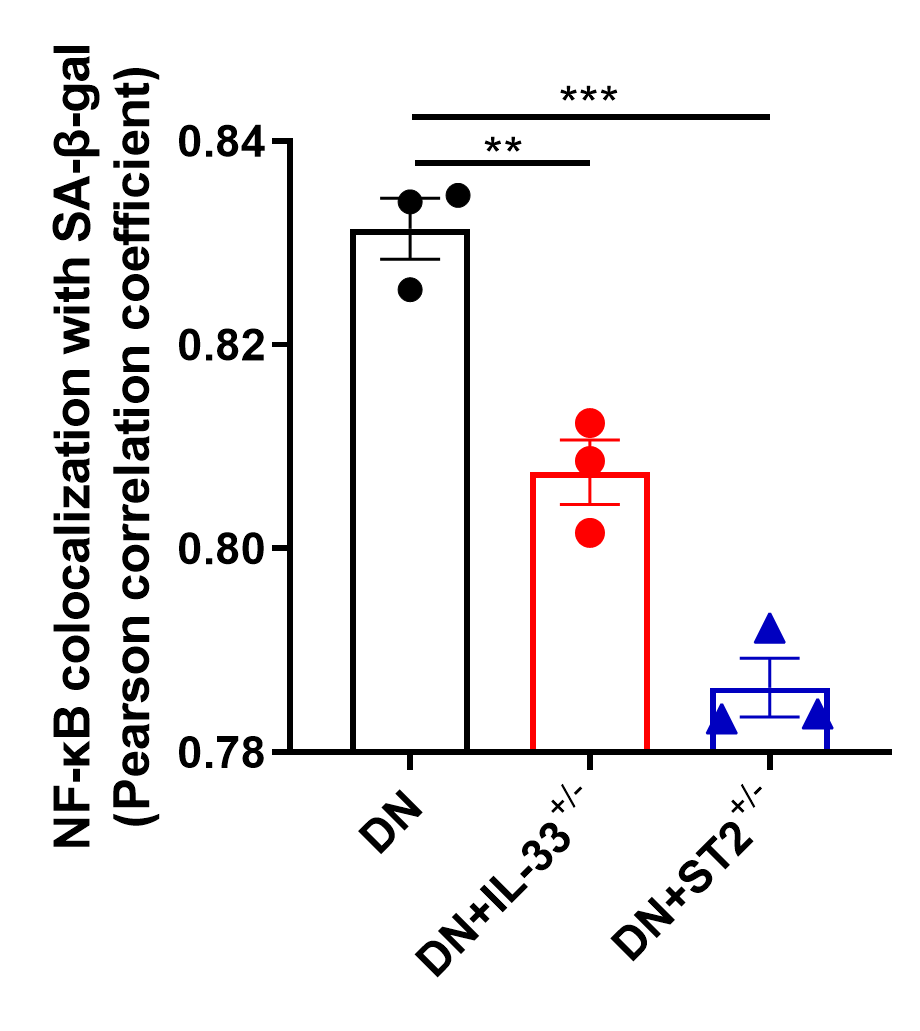


**G**


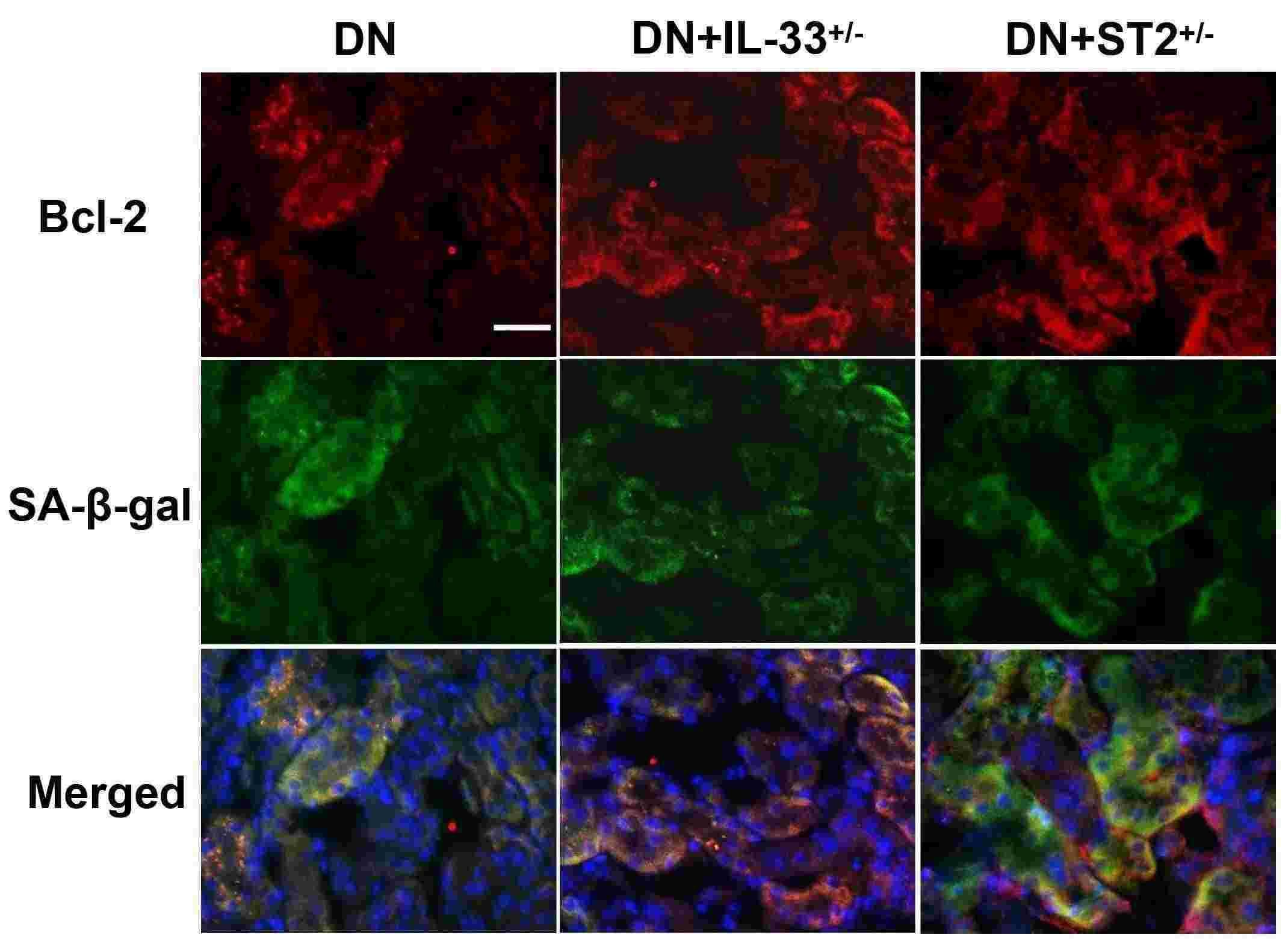


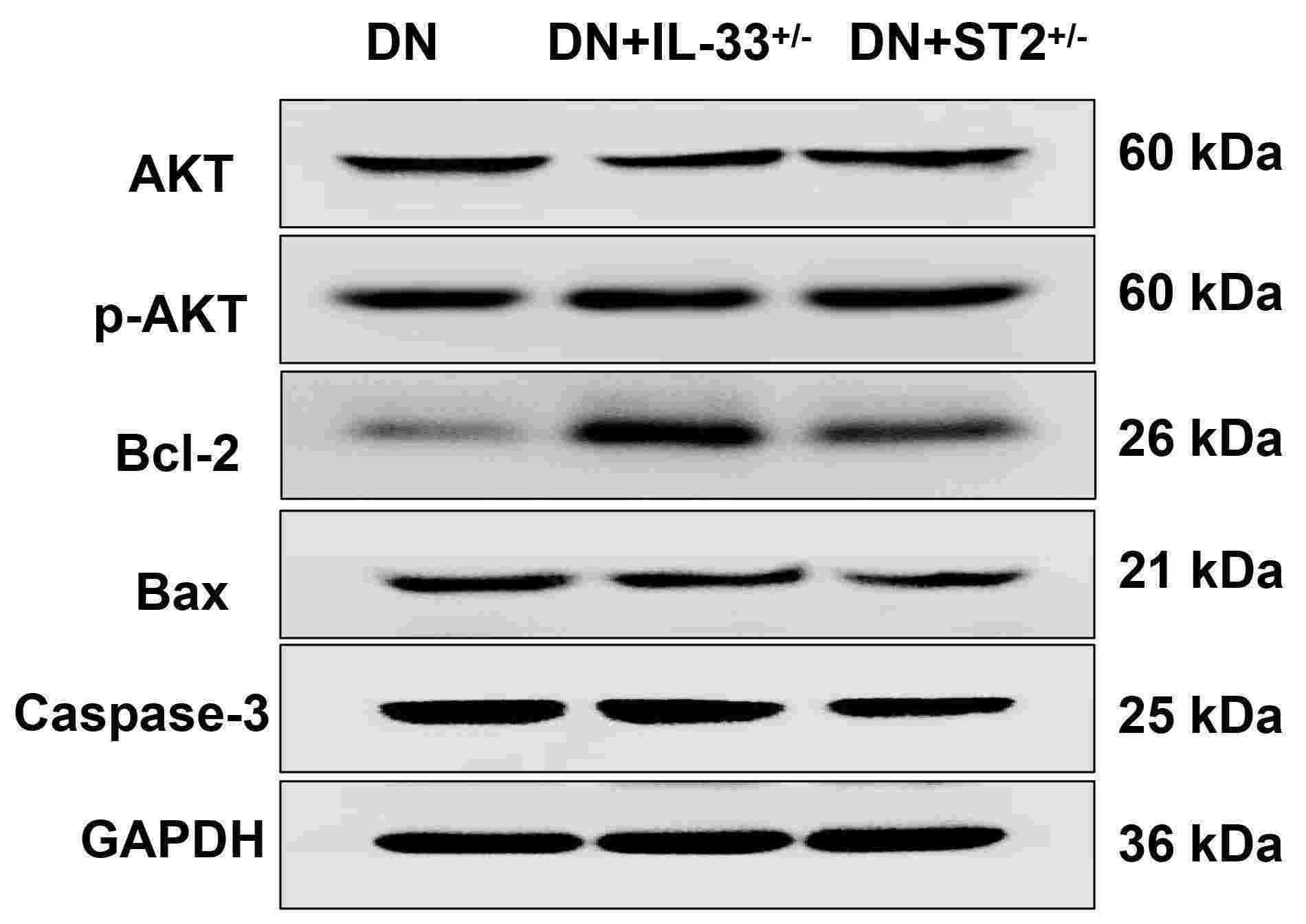


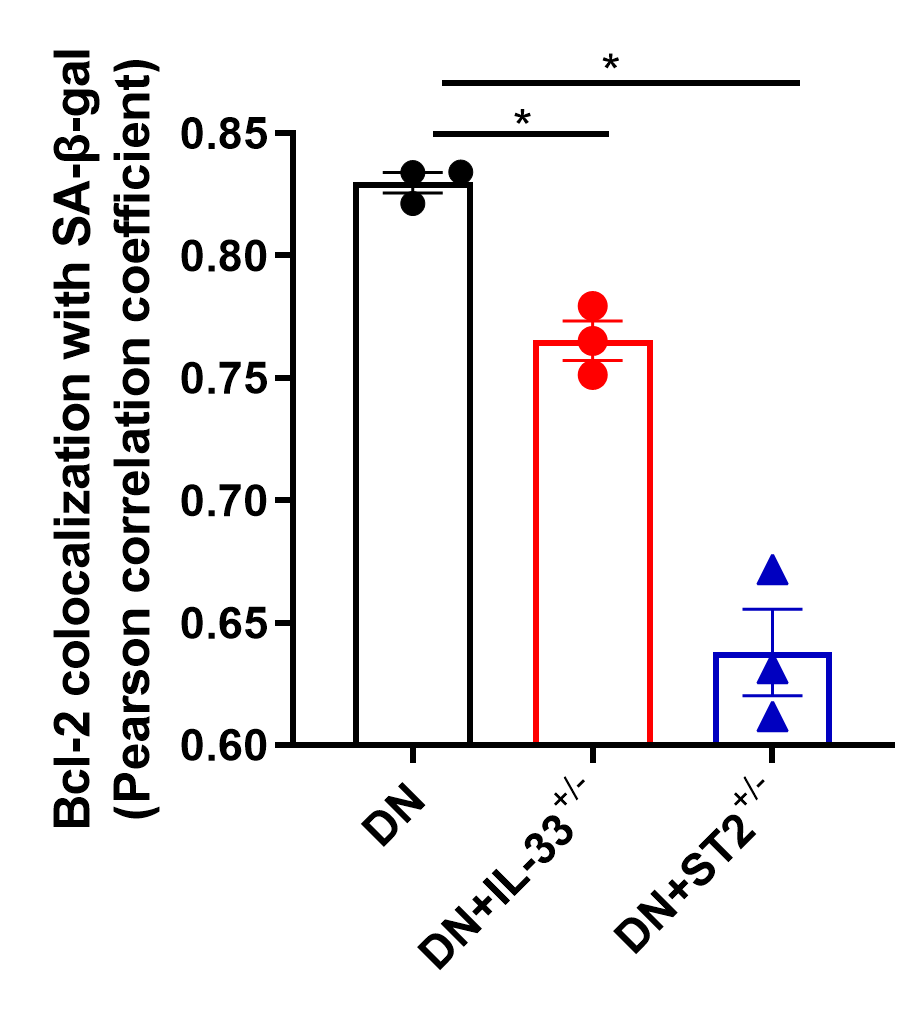


**I**


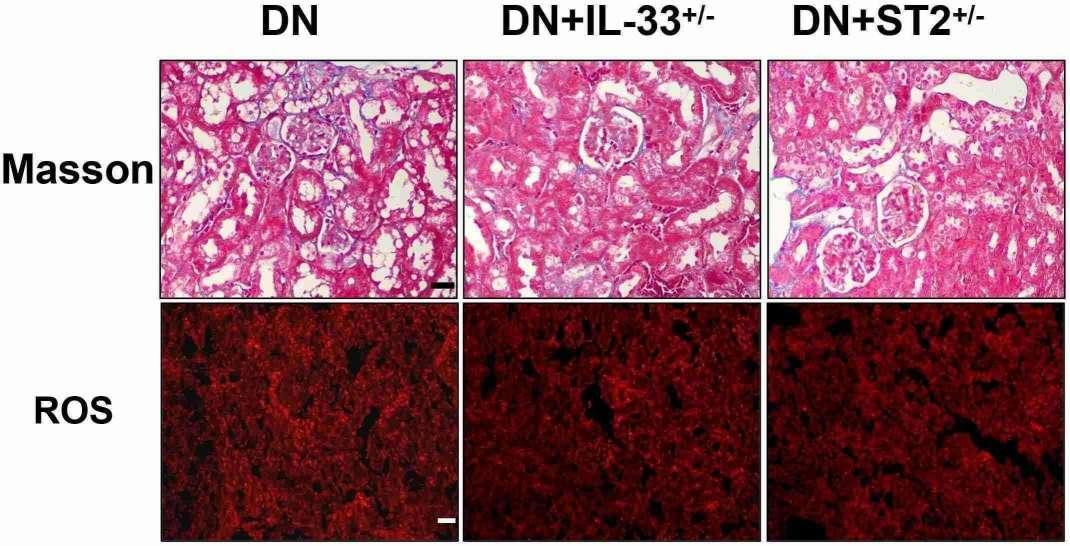


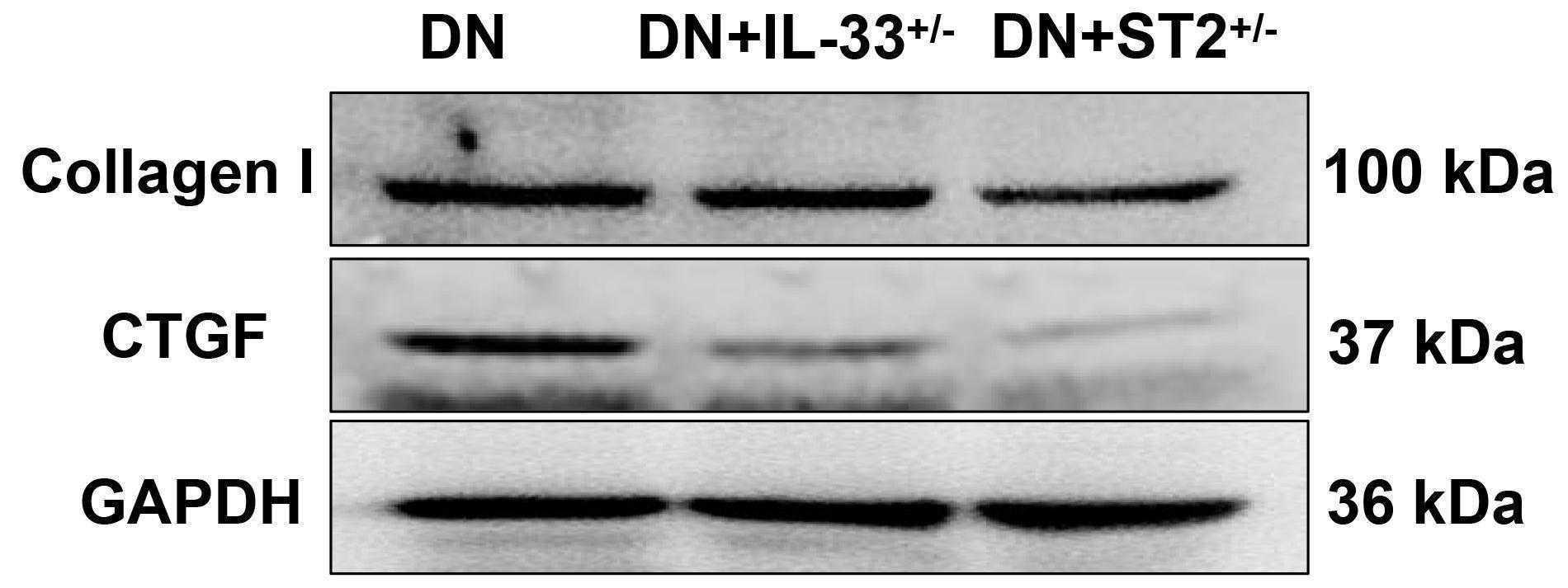


Figure S5. Renal senescence and aging are improved in IL-33^+/-^ and ST2^+/-^ DN mice. (A) SA-β-gal positive area and Ki67 positive cell number. (B and C) Immunoblots and densitometric analysis of p53, p21, p16, ATM, and γH2AX. (D and E) Colocalization of SASP-associated proteins (IL-6 and NF-κB, red) with senescent cells (SA-β-gal, green). (F) Immunoblots and densitometric analysis of AKT, p-AKT, Bcl-2, Bax, and Caspase-3. (G) Bcl-2 (red) was co-localized with SA-β-gal (green). (H) Immunoblots and densitometric analysis of Collagen I and CTGF. (I) Masson positive area and ROS level. Scale bar: 20 μm. **P* < 0.05, ***P* < 0.01, ****P* < 0.001.


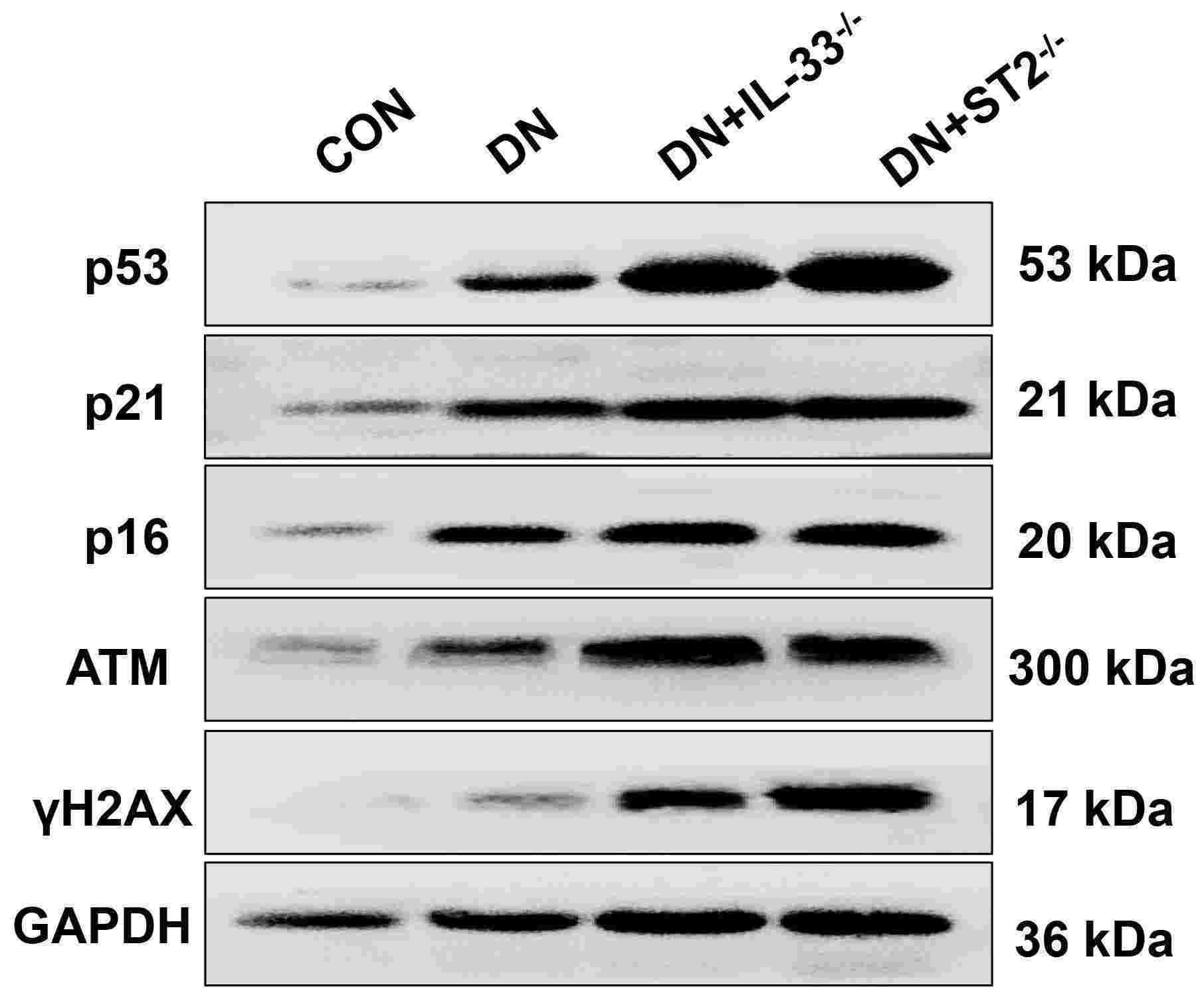
**Supplementary Figure S6**

**B**

**A**


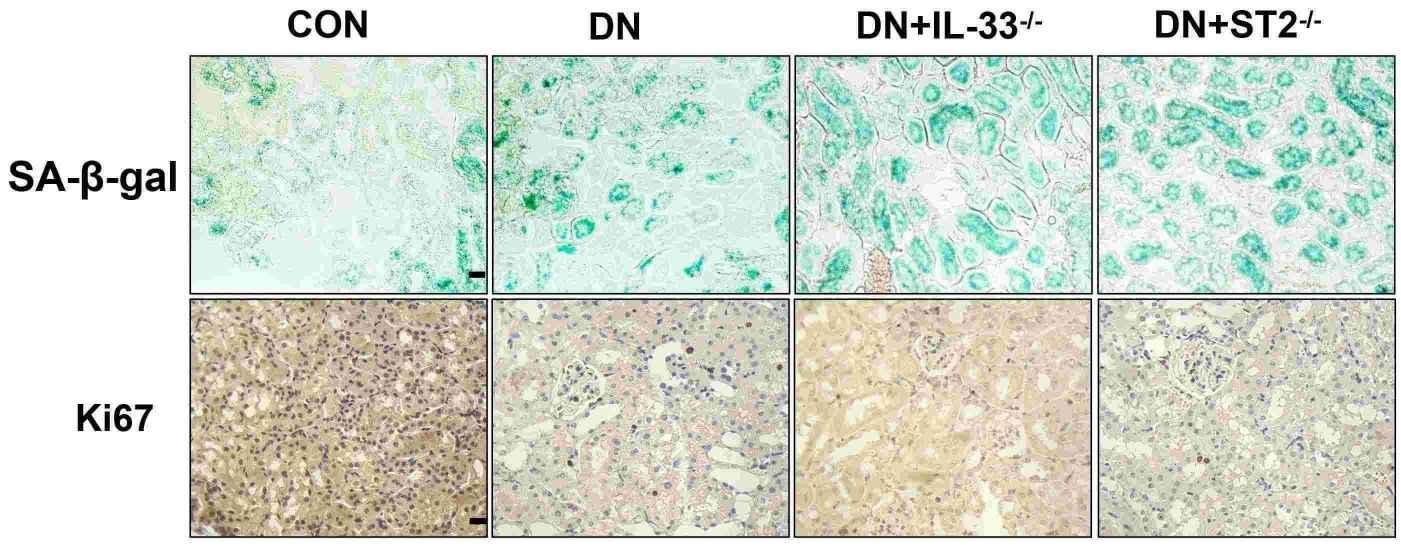


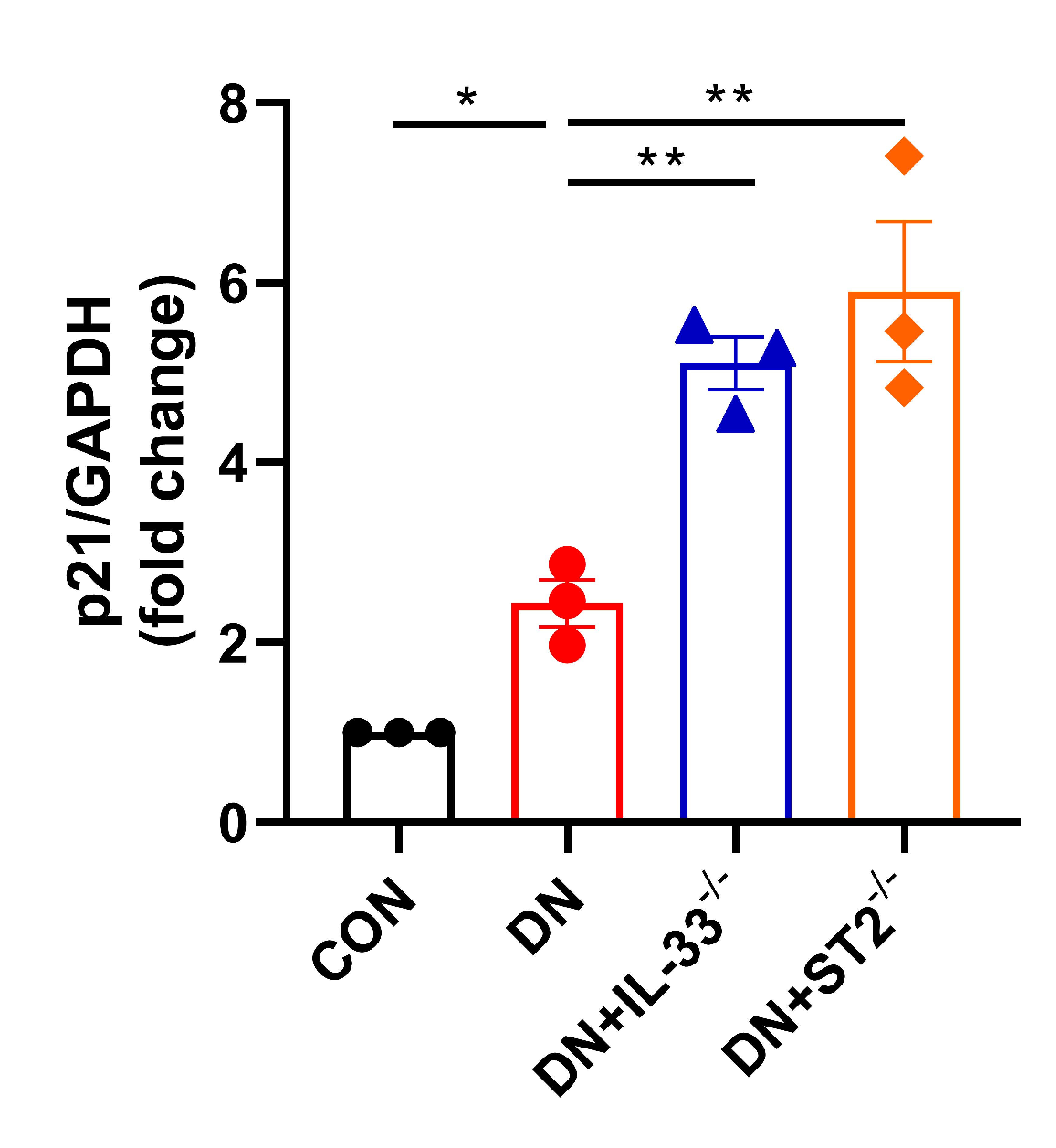

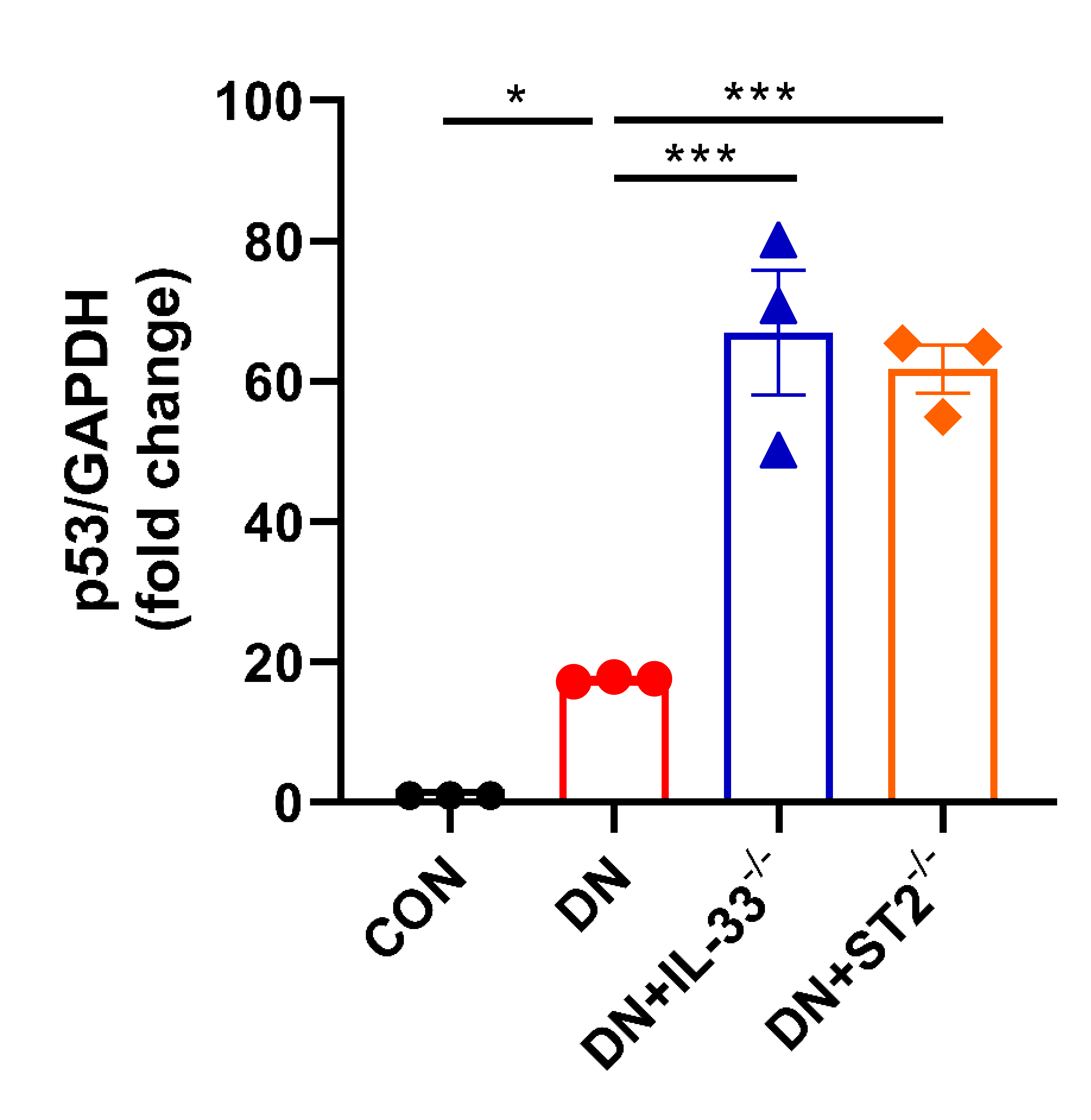


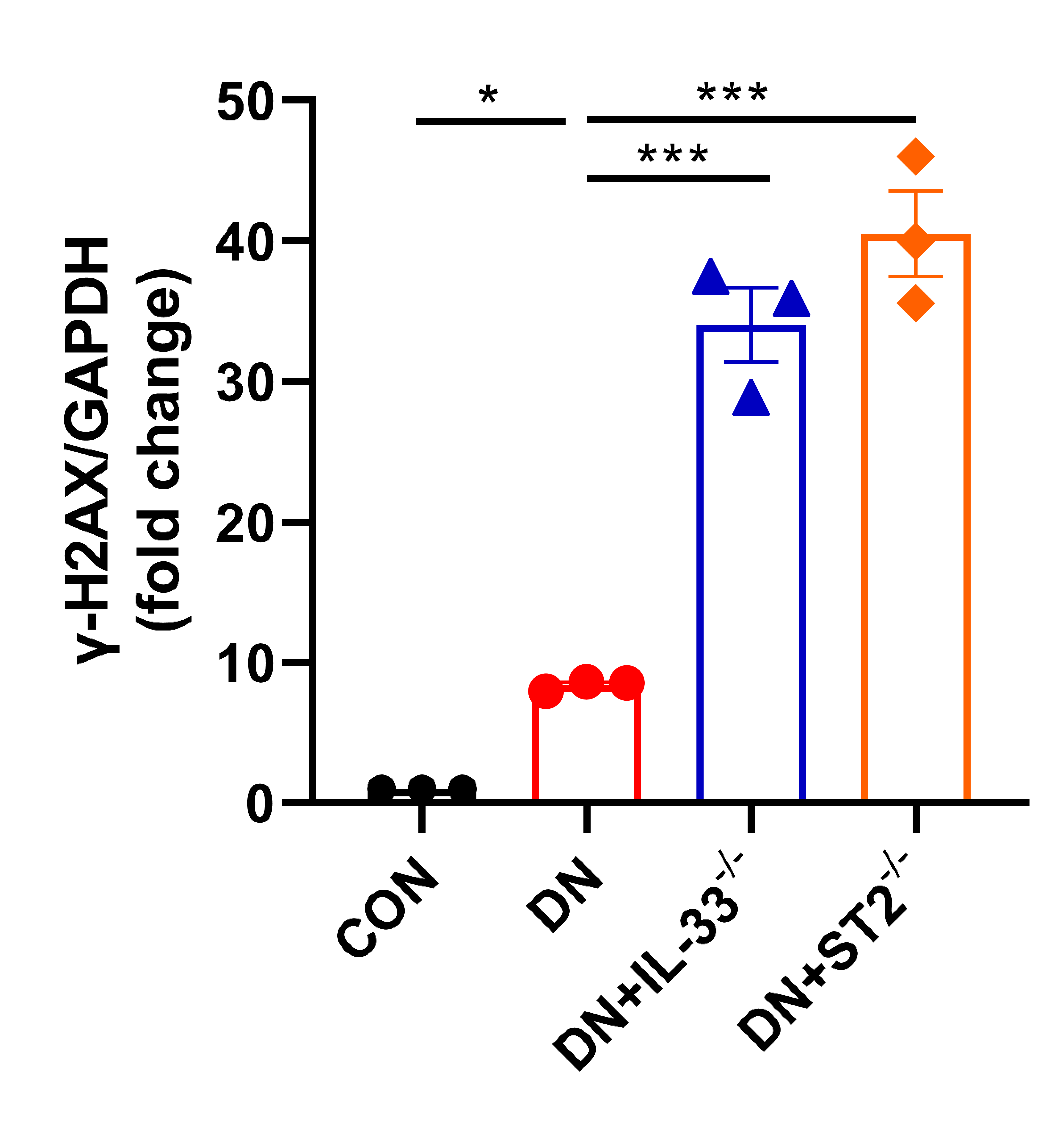

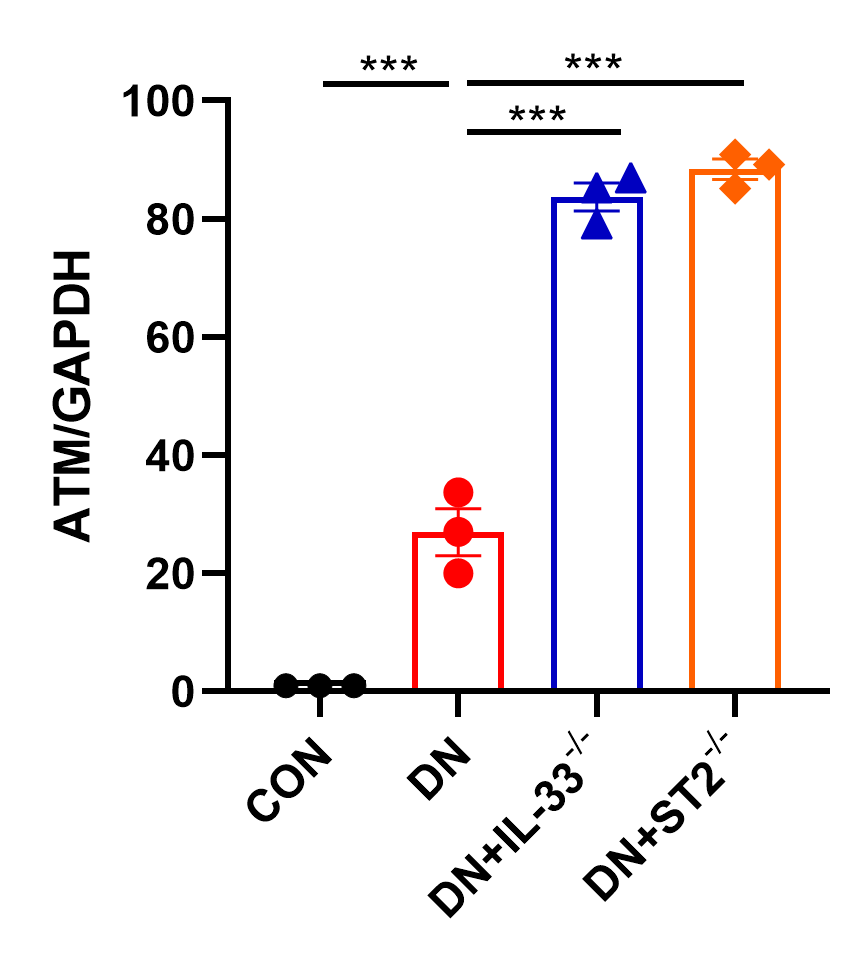

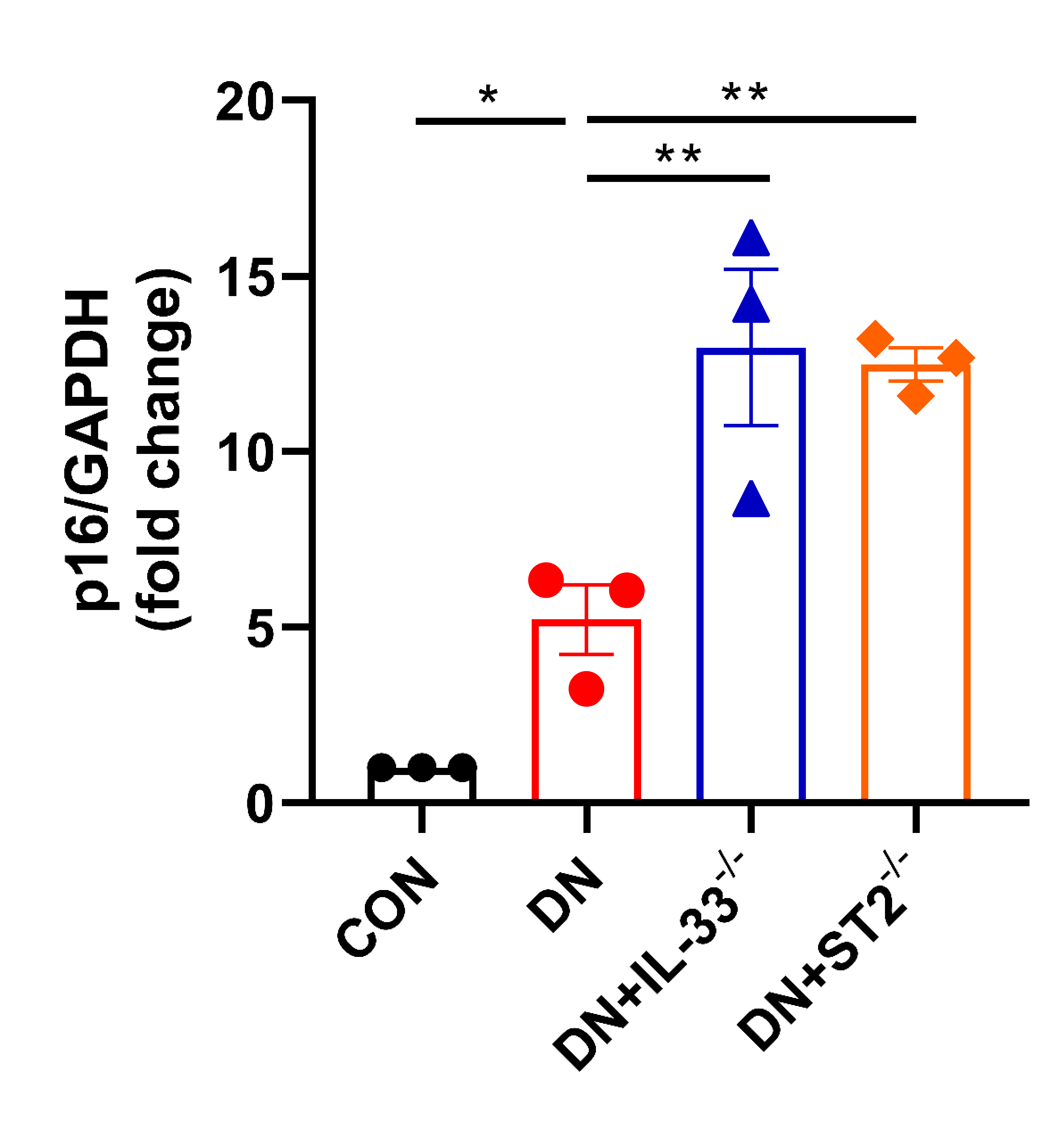

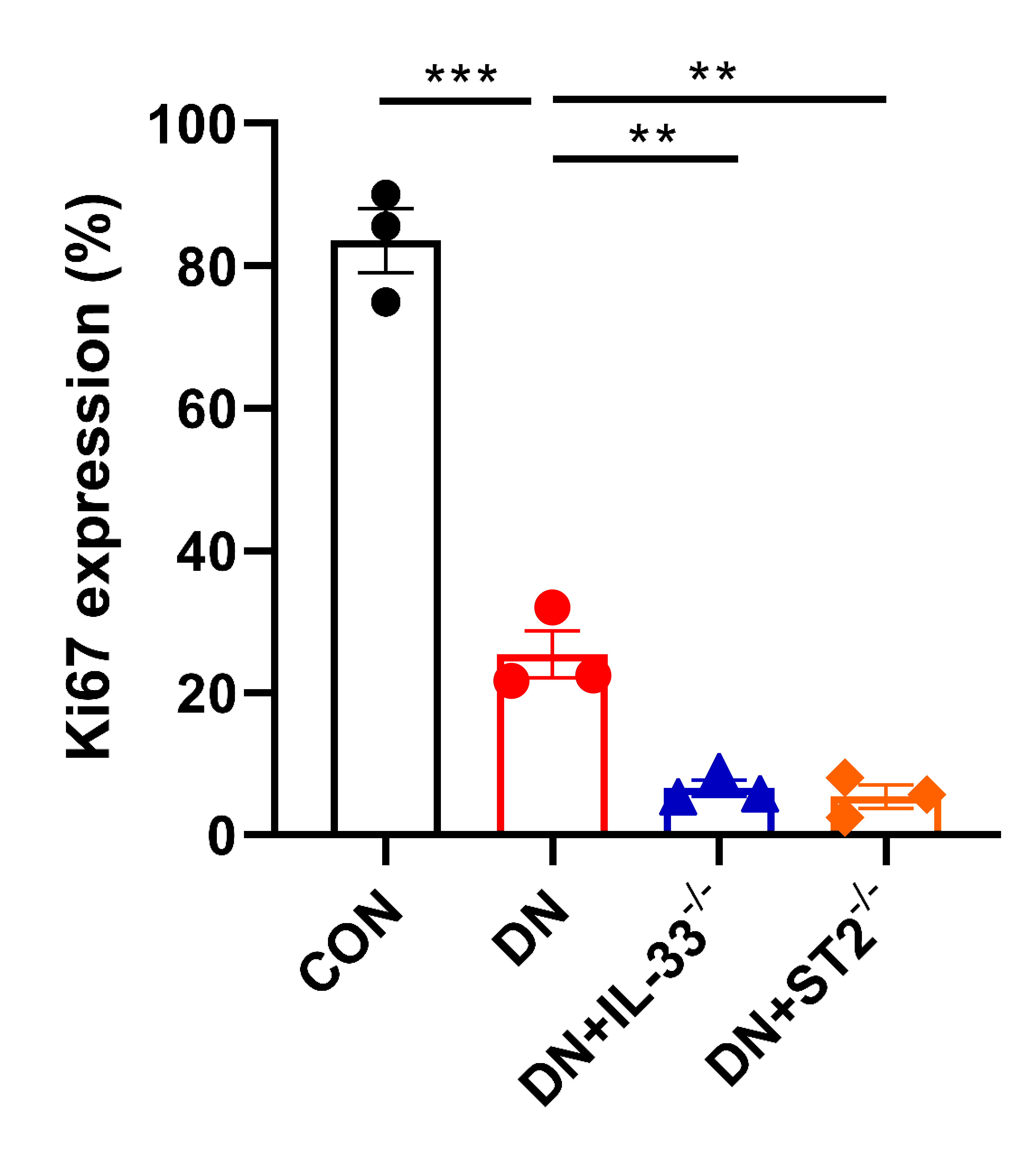


**D**

**C**


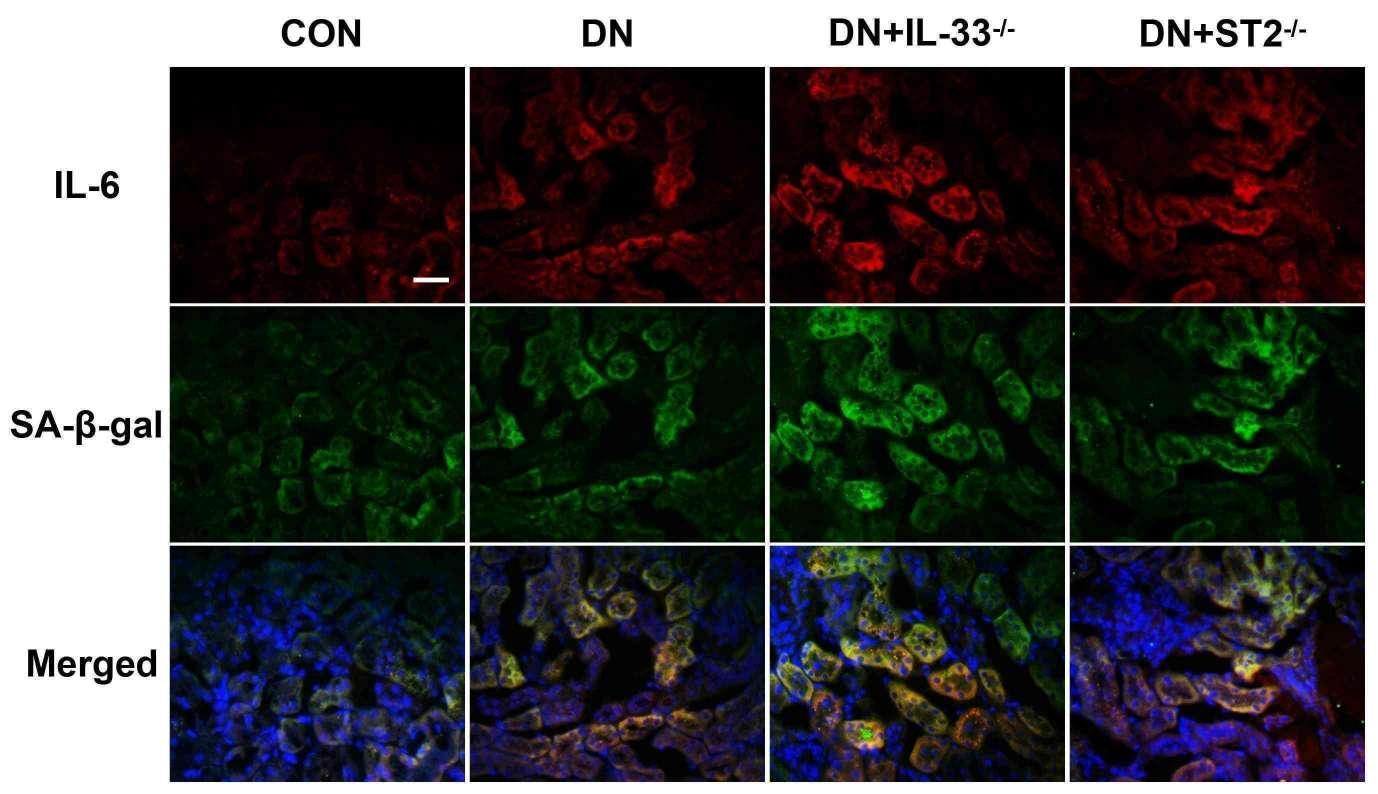


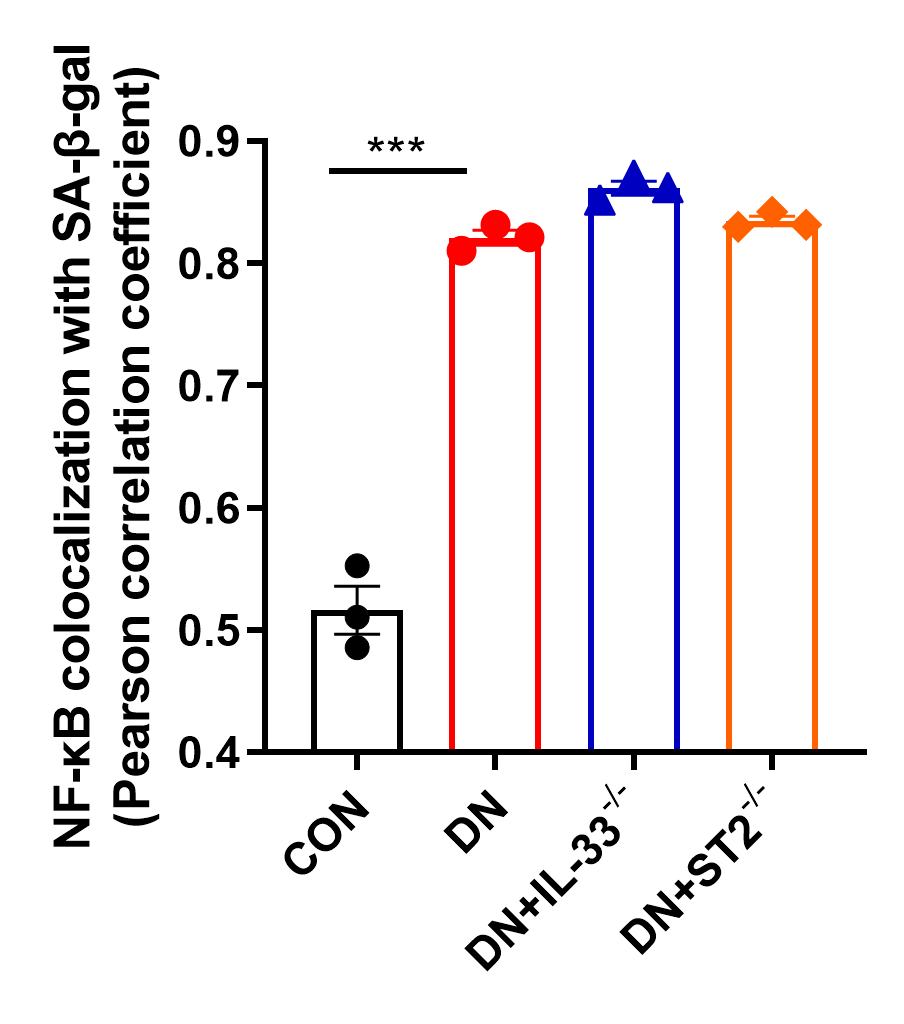

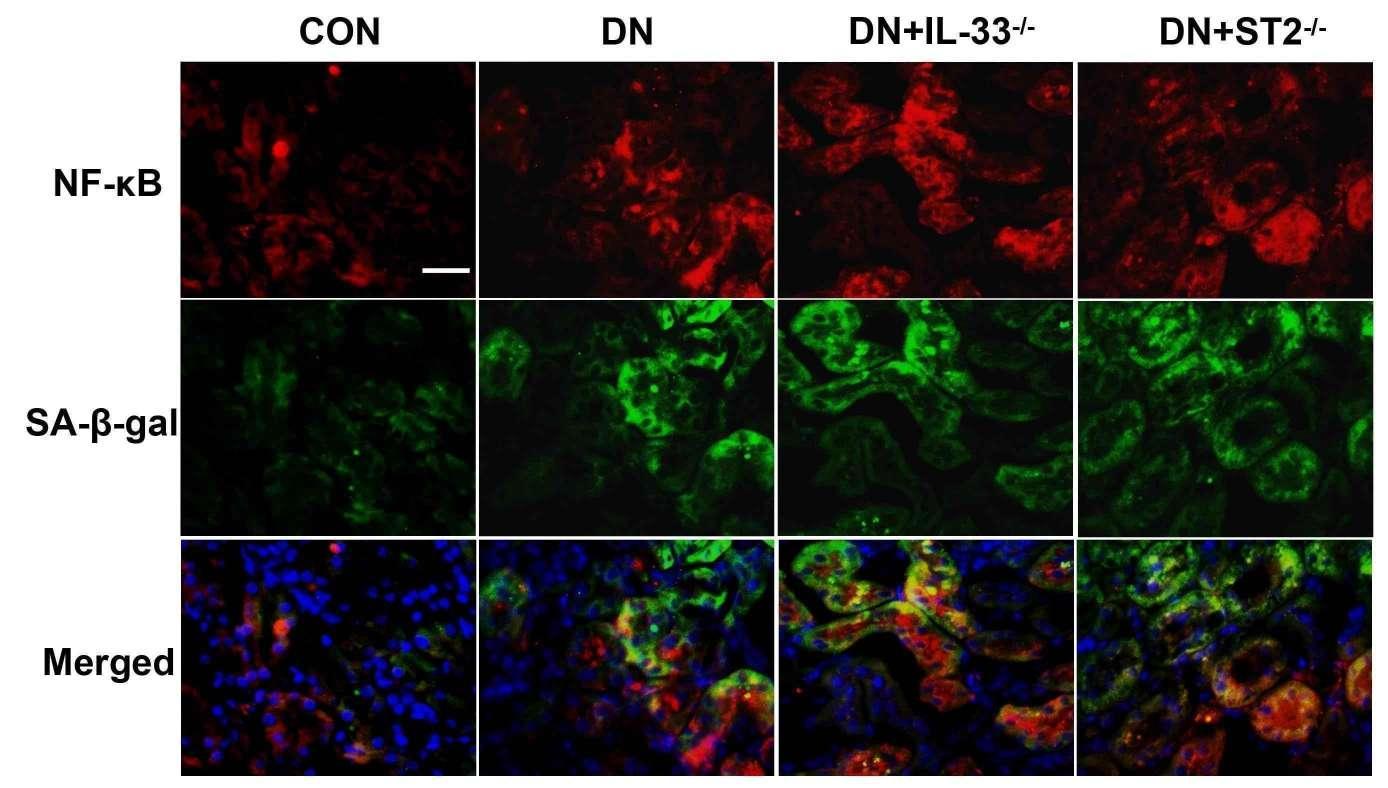


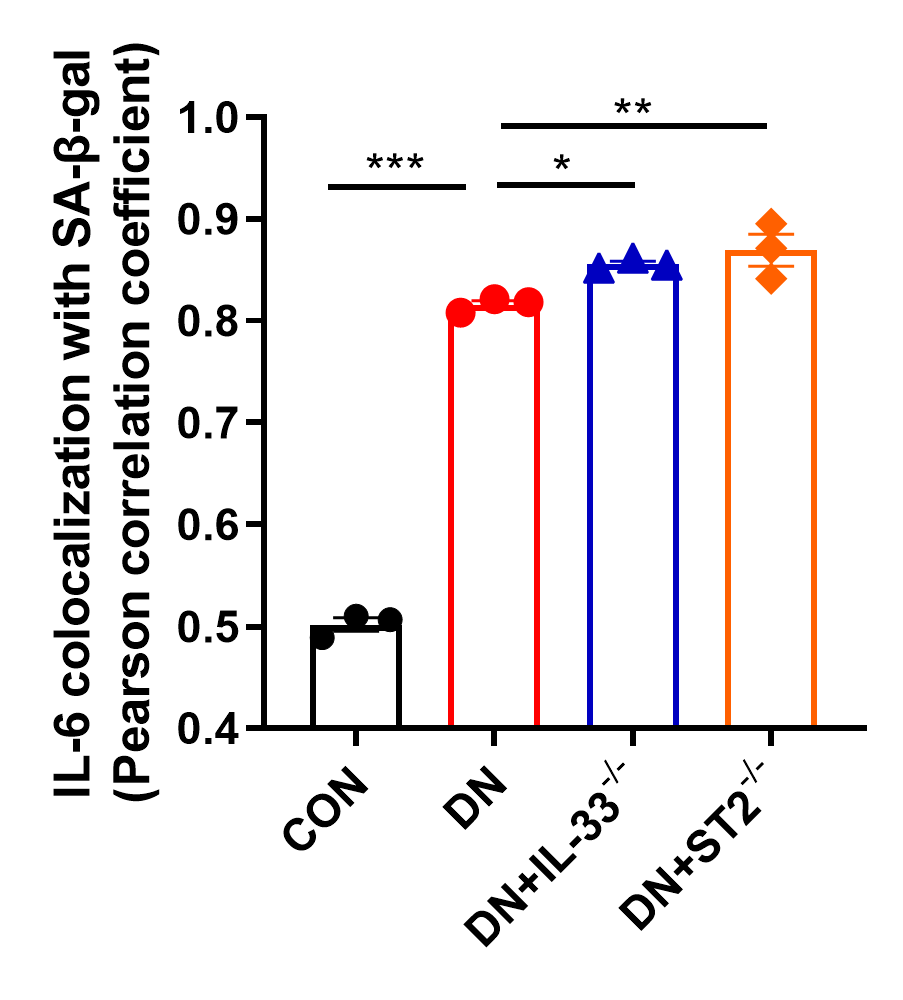


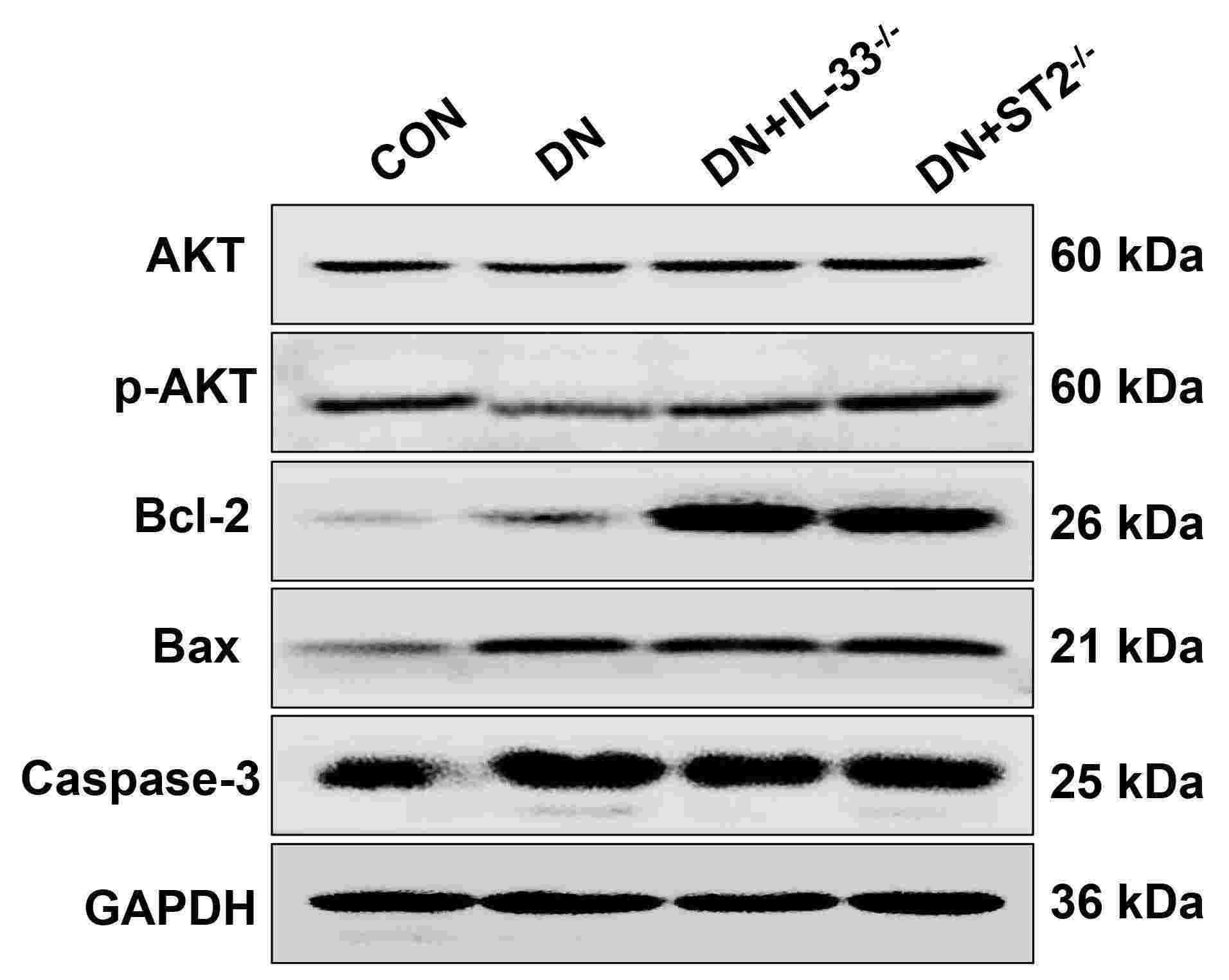


**F**

**E**


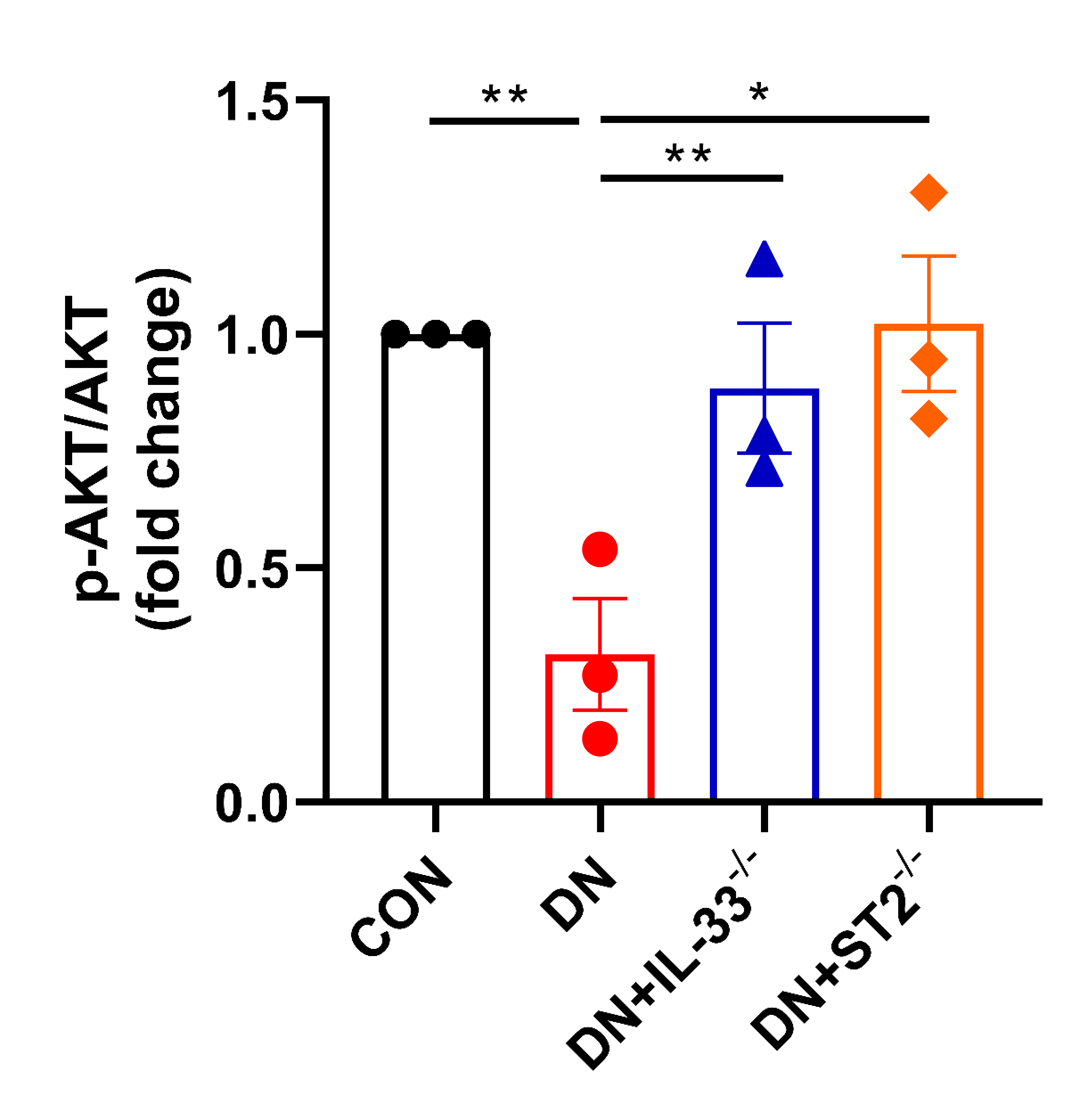

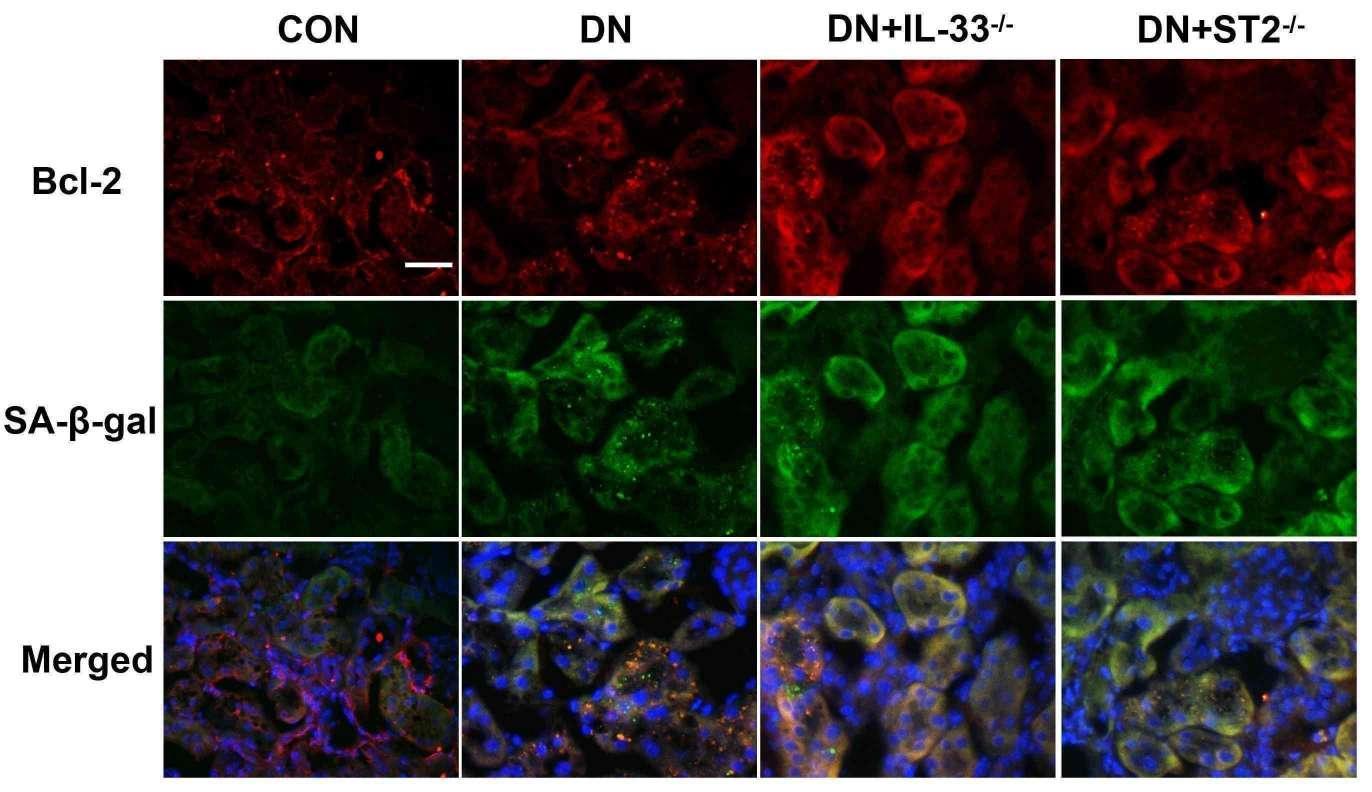

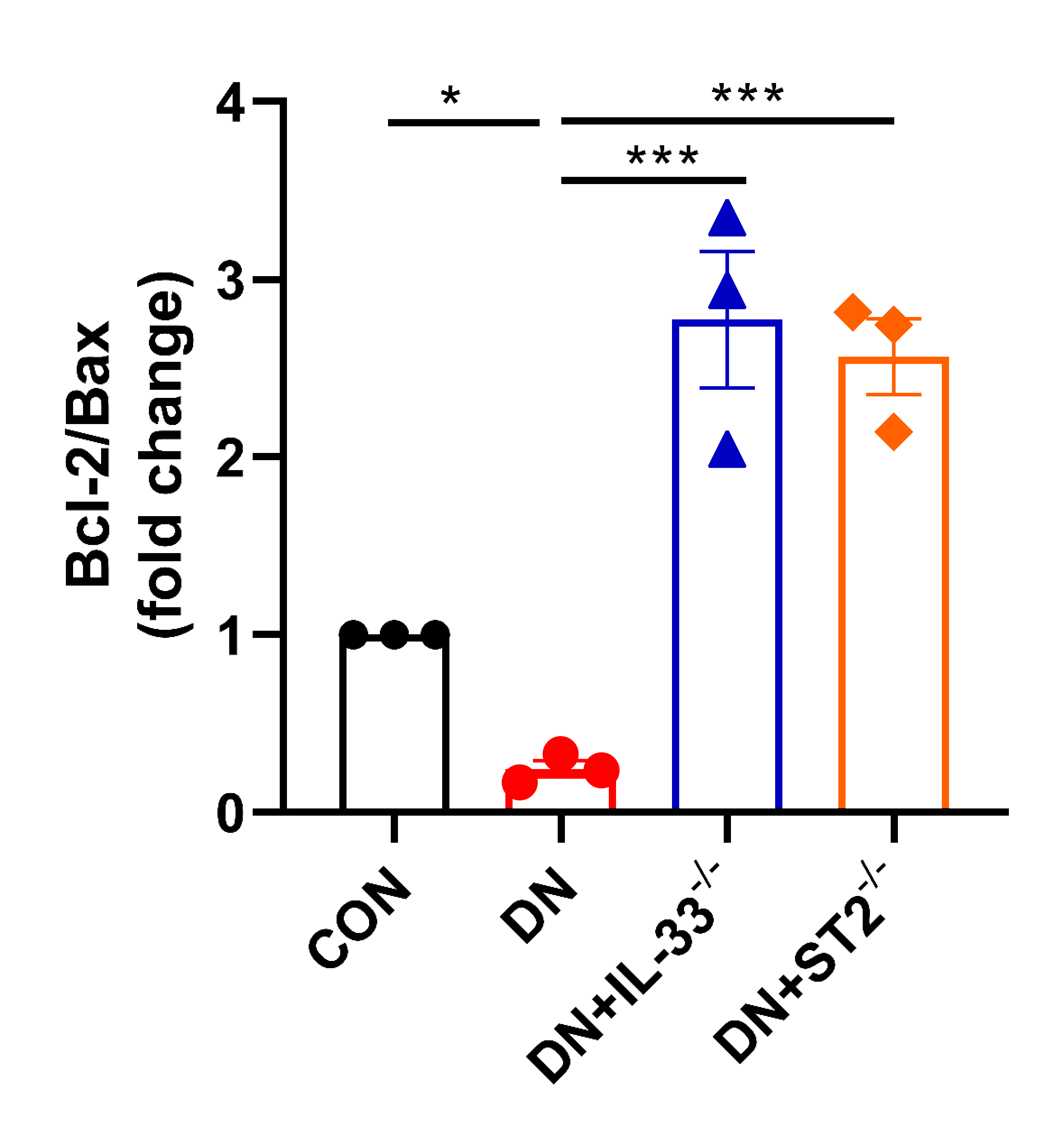


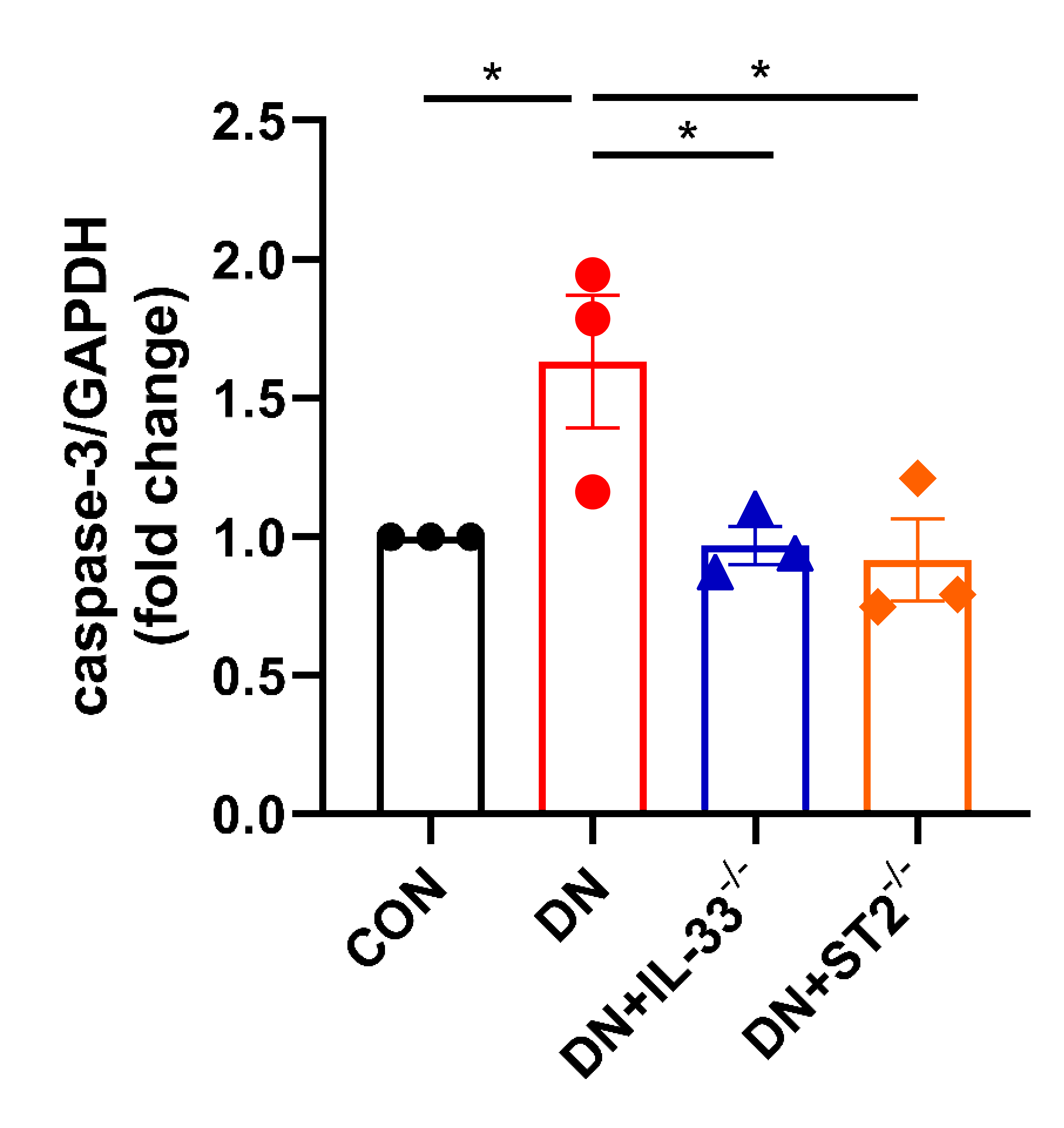

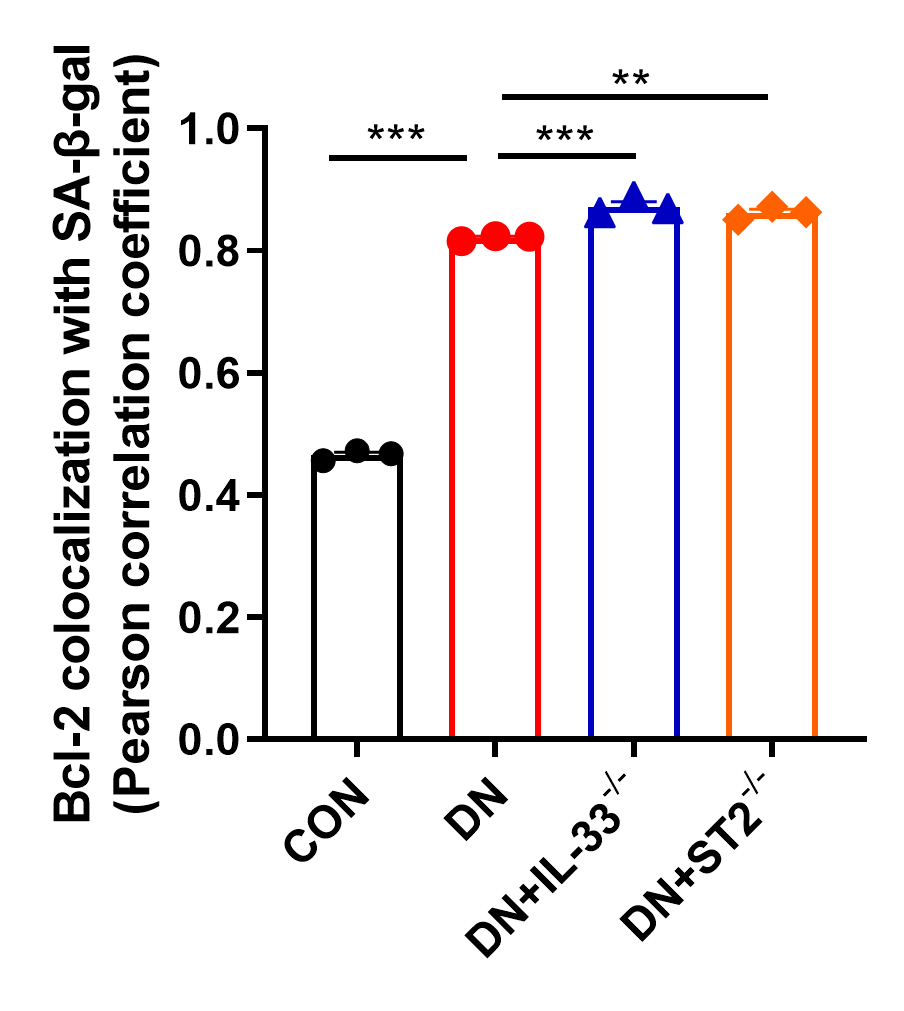


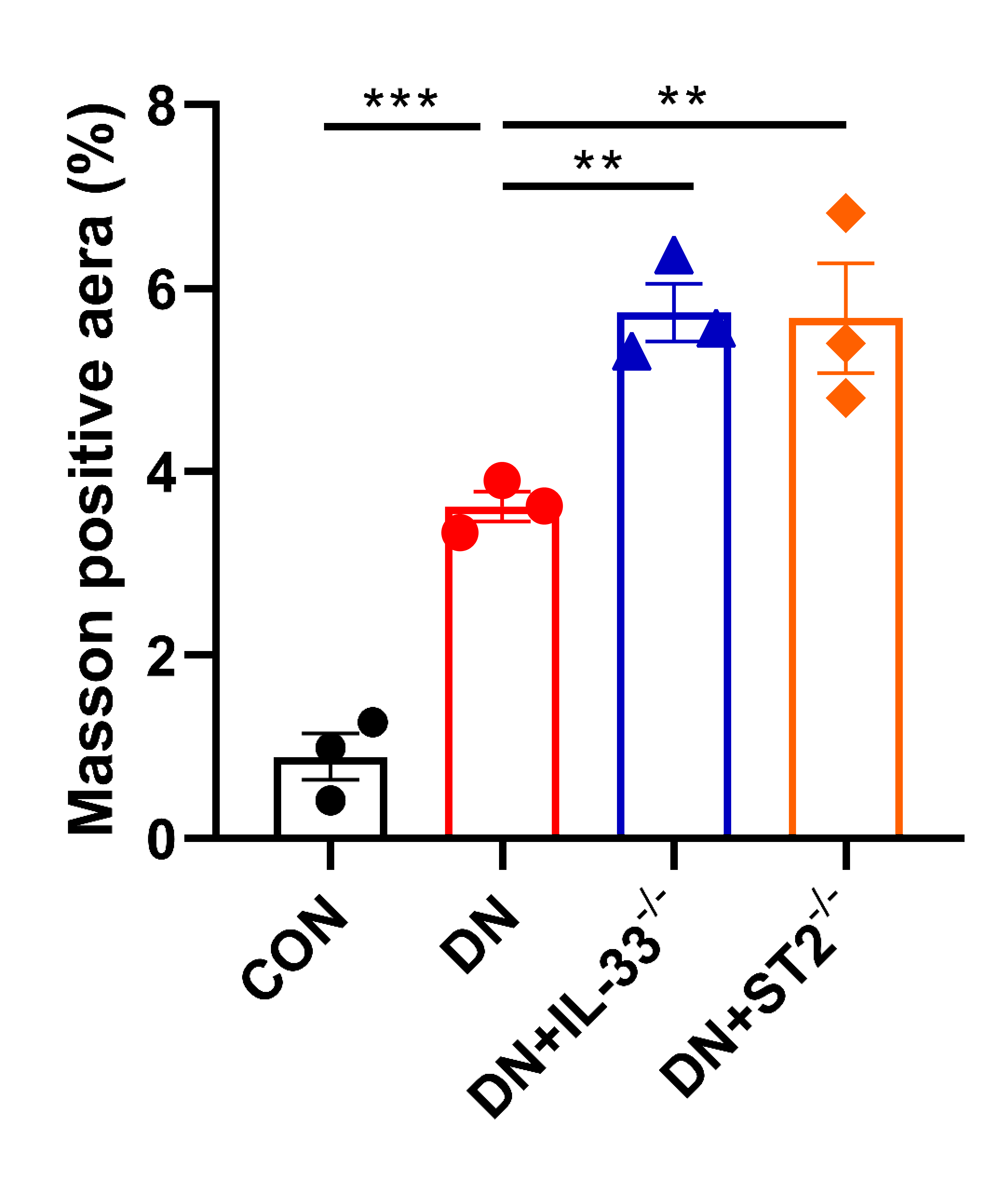


**H**

**G**


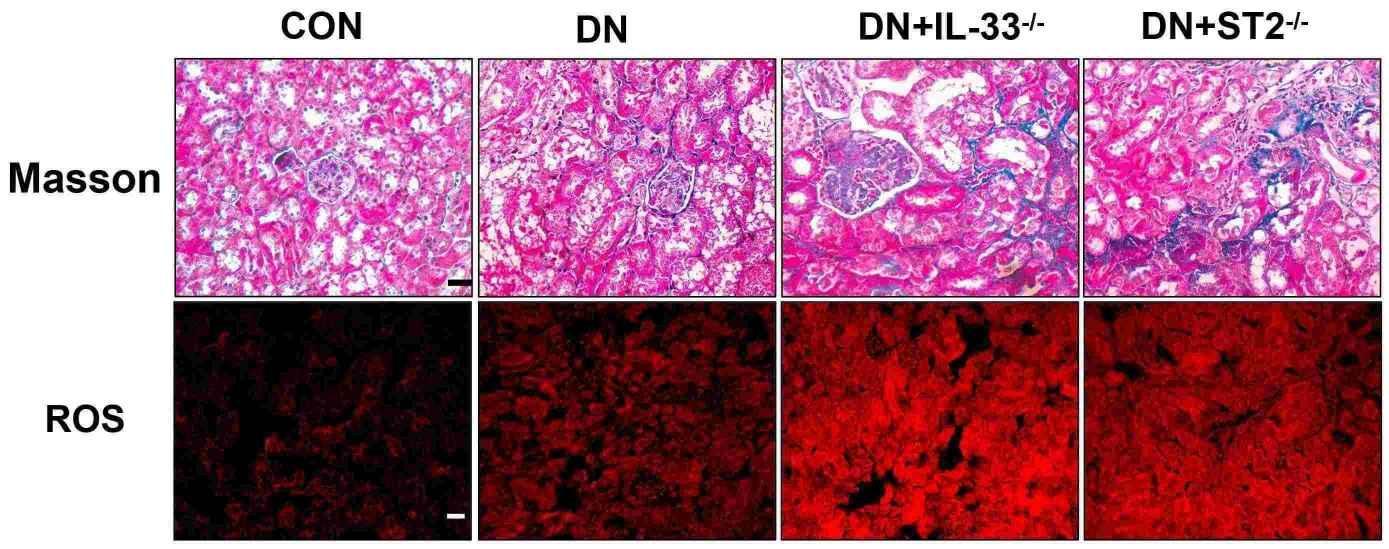

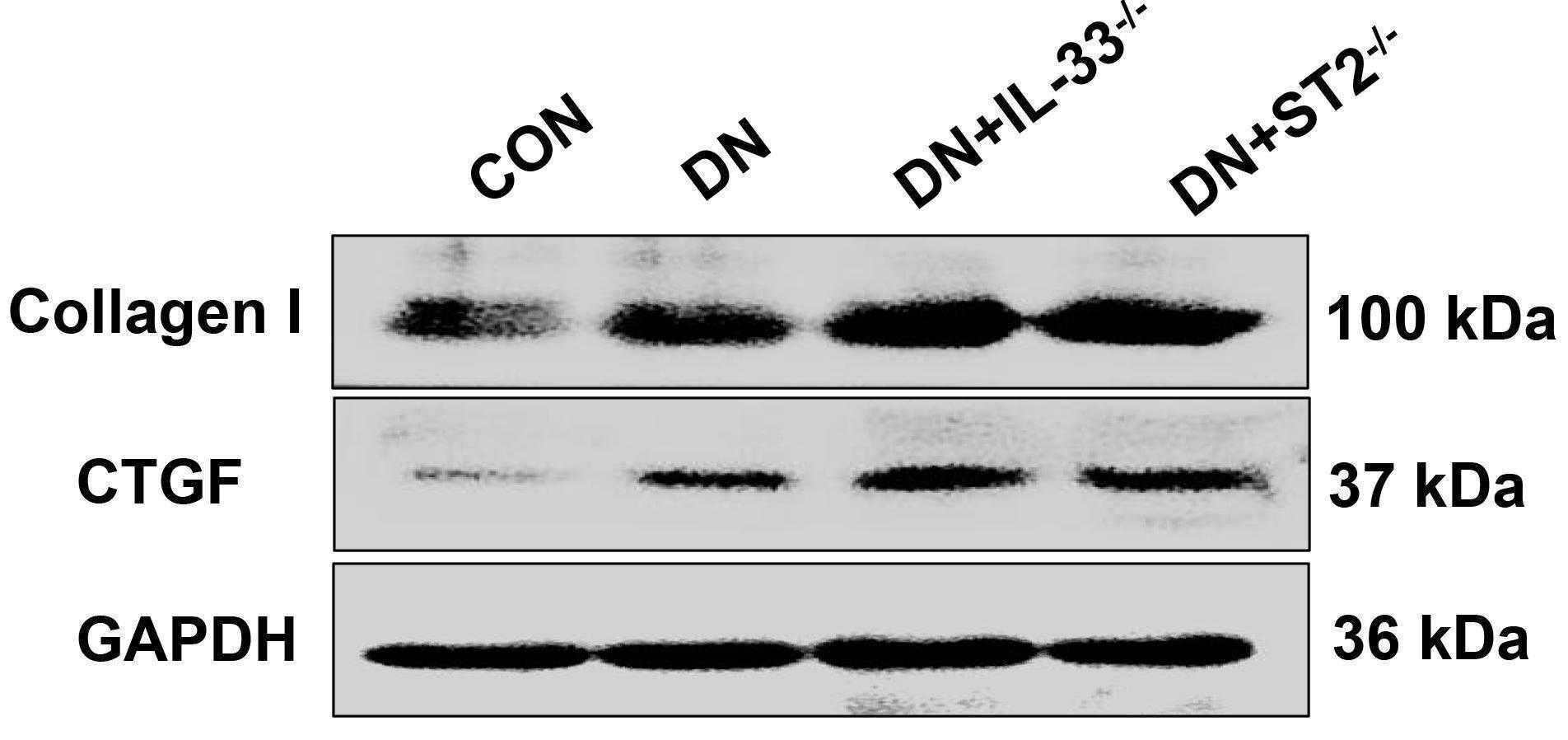


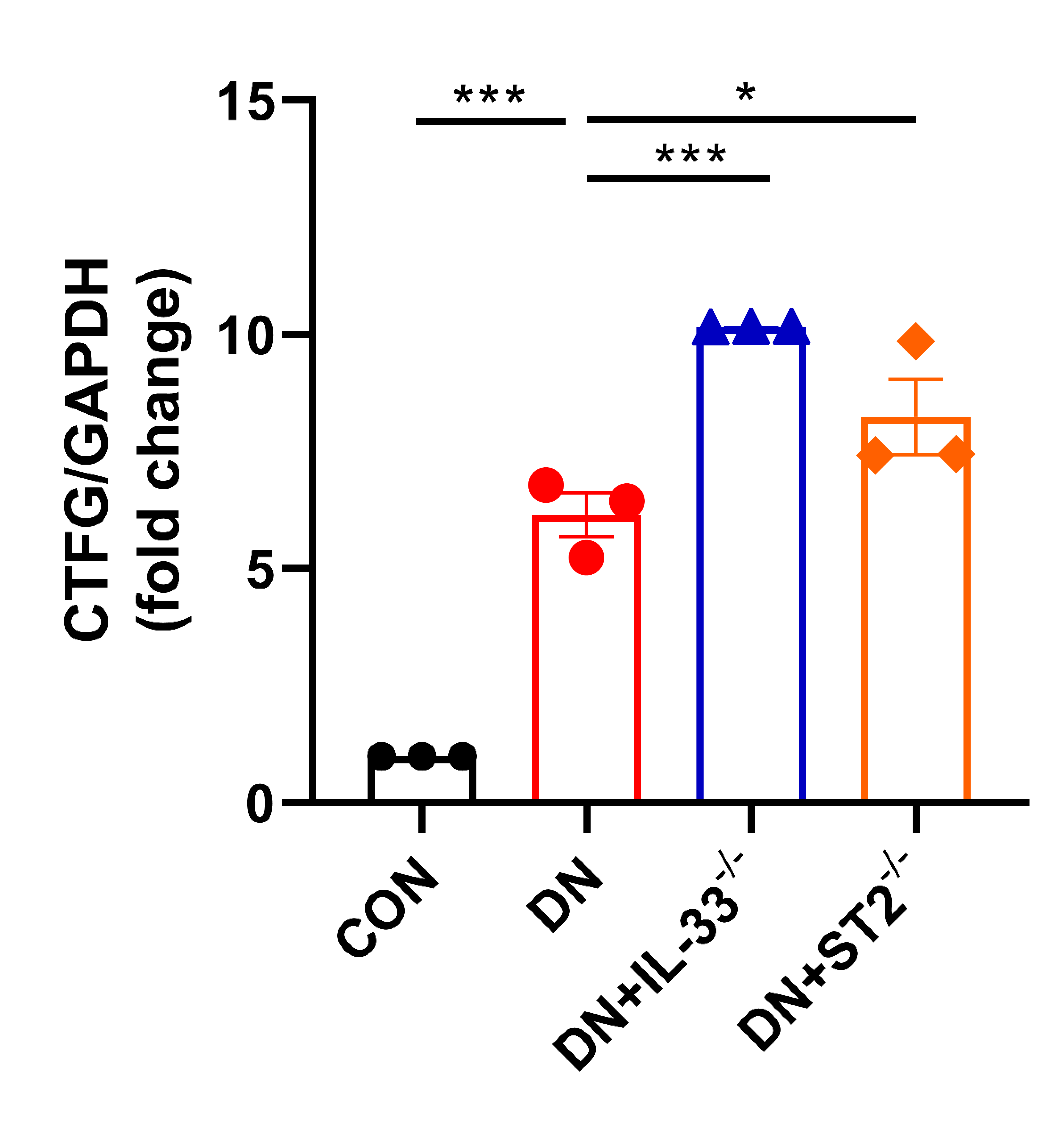

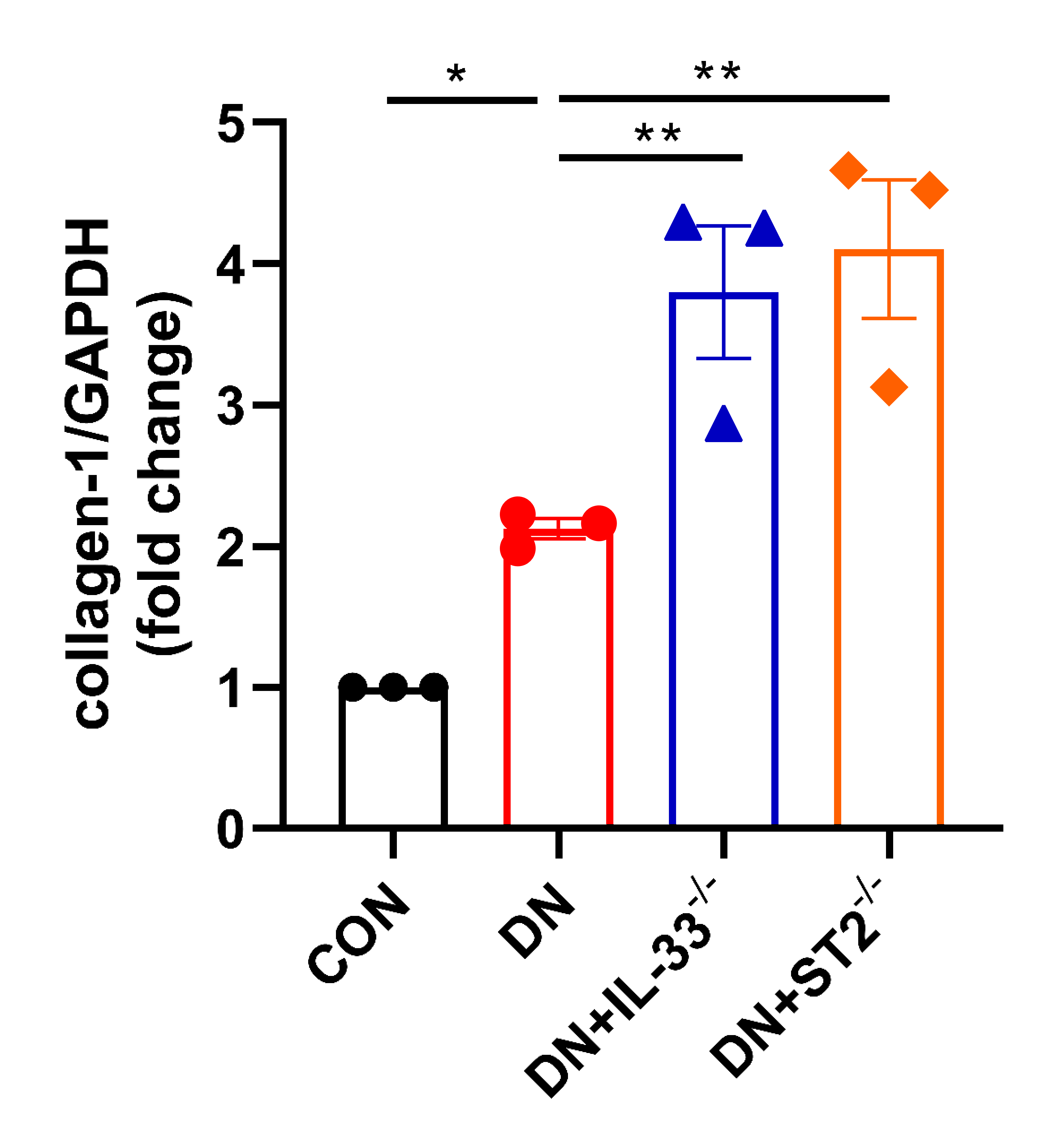


Figure S6. IL-33 or ST2 knockout aggravates renal senescence and aging in DN mice. (A) The staining of SA-β-gal and Ki67 in IL-33^-/-^ and ST2^-/-^ DN mice and its control groups. (B) The protein levels of p53, p21, p16, ATM, and γH2AX. (C and D) Colocalization analysis of NF-κB (red) and IL-6 (red) with SA-β-gal (green). (E) Immunoblots and densitometric analysis of AKT, p-AKT, Bcl-2, Bax, and Caspase-3. (F) Colocalization of Bcl-2 (red) with SA-β-gal (green). (G) Immunoblots of Collagen I and CTGF. (H) Masson and ROS fluorescent staining in the kidney. Scale bar: 20 μm. **P* < 0.05, ***P* < 0.01, ****P* < 0.001.

**Supplementary Figure S7**


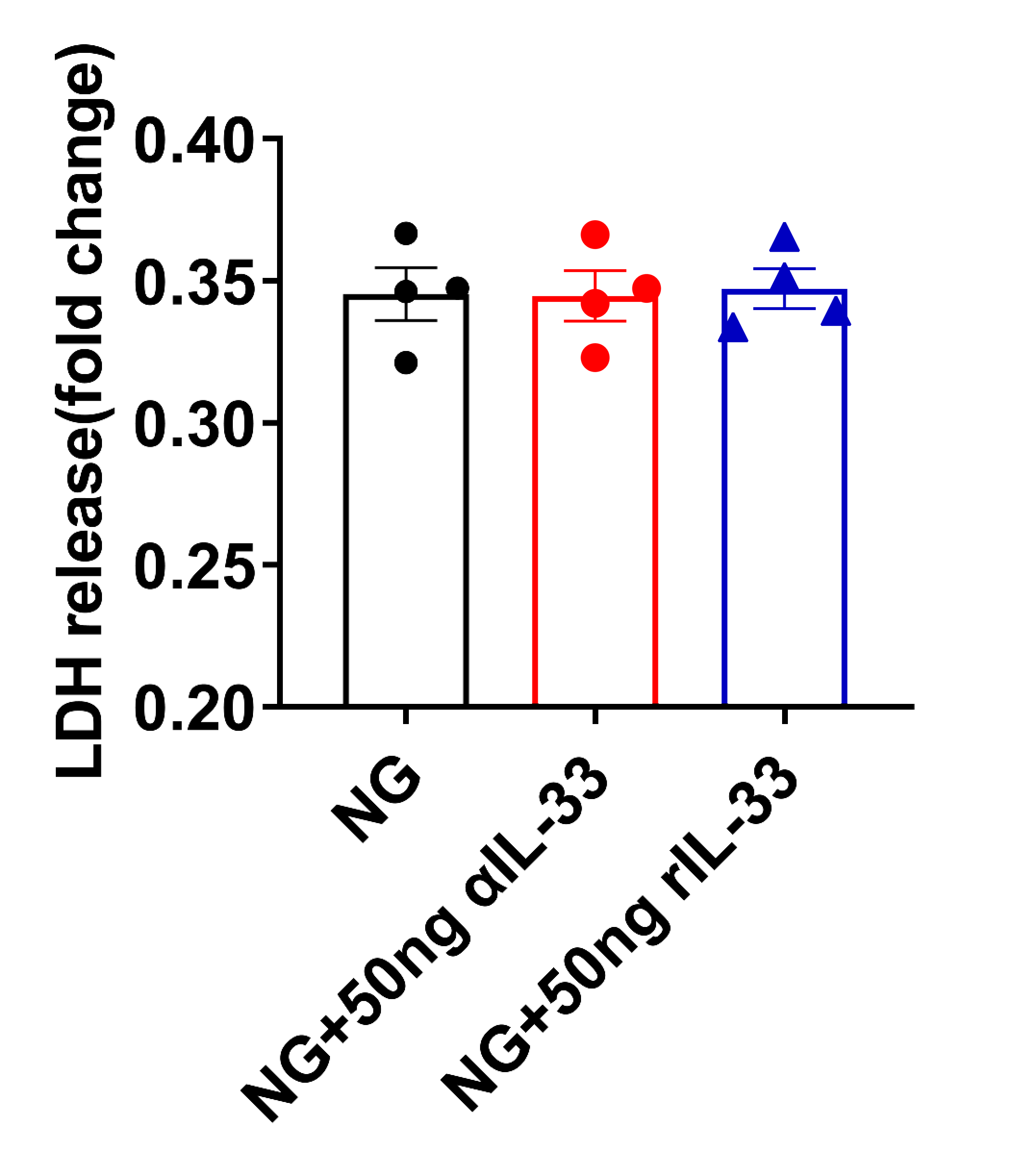


**A**


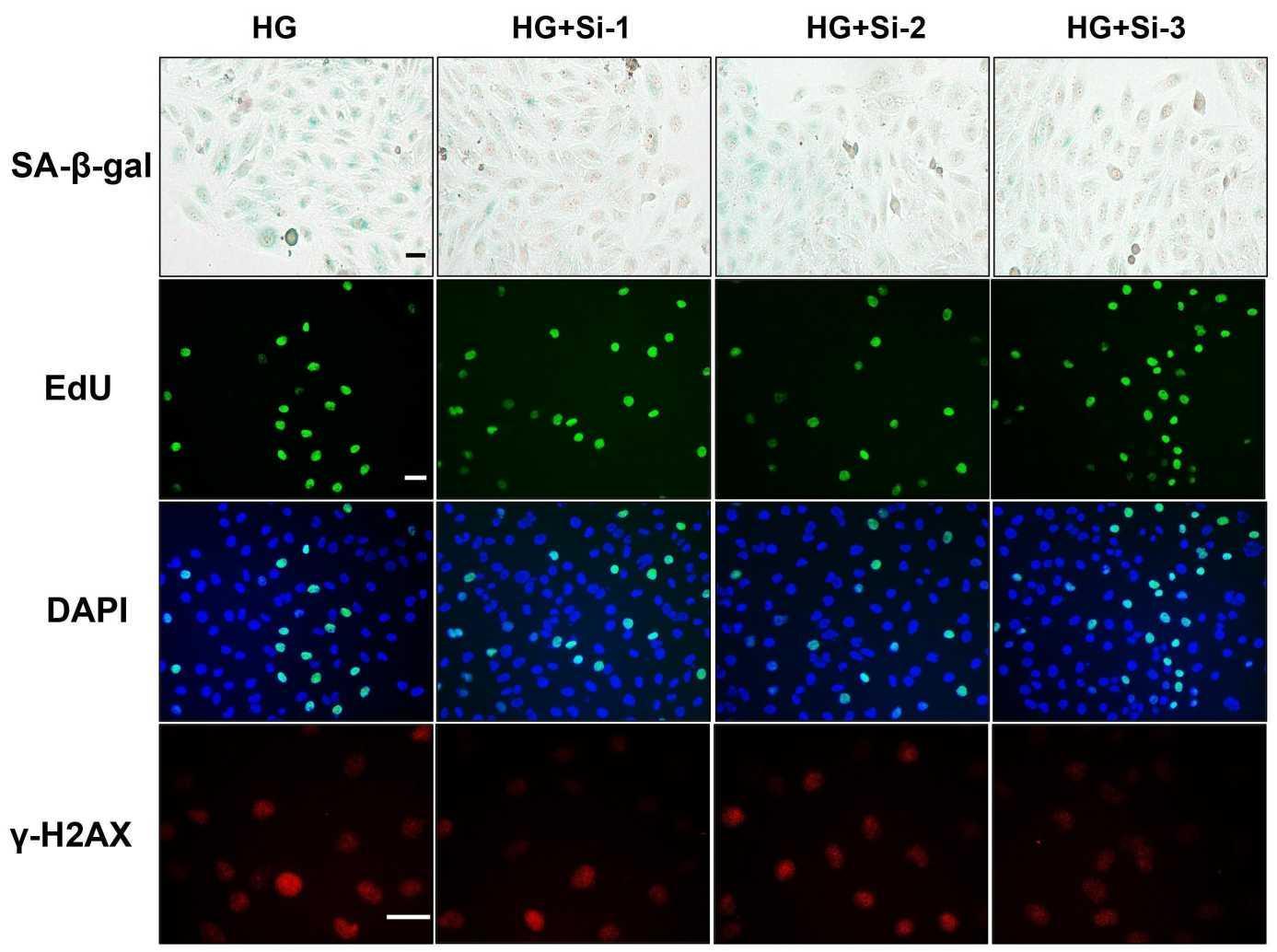

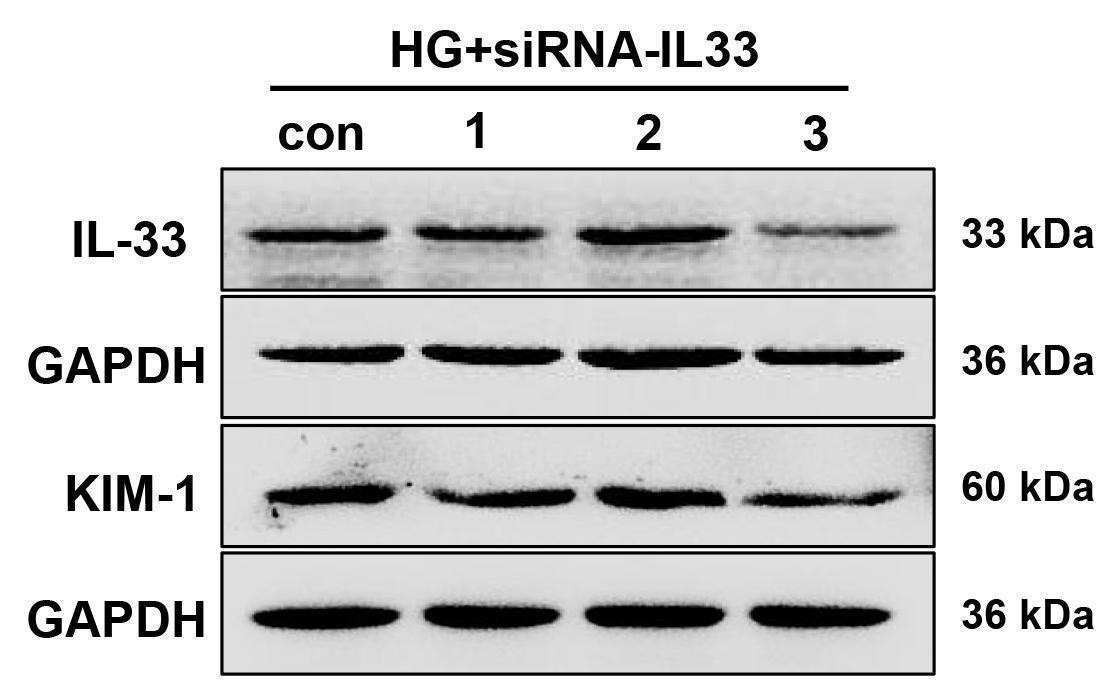

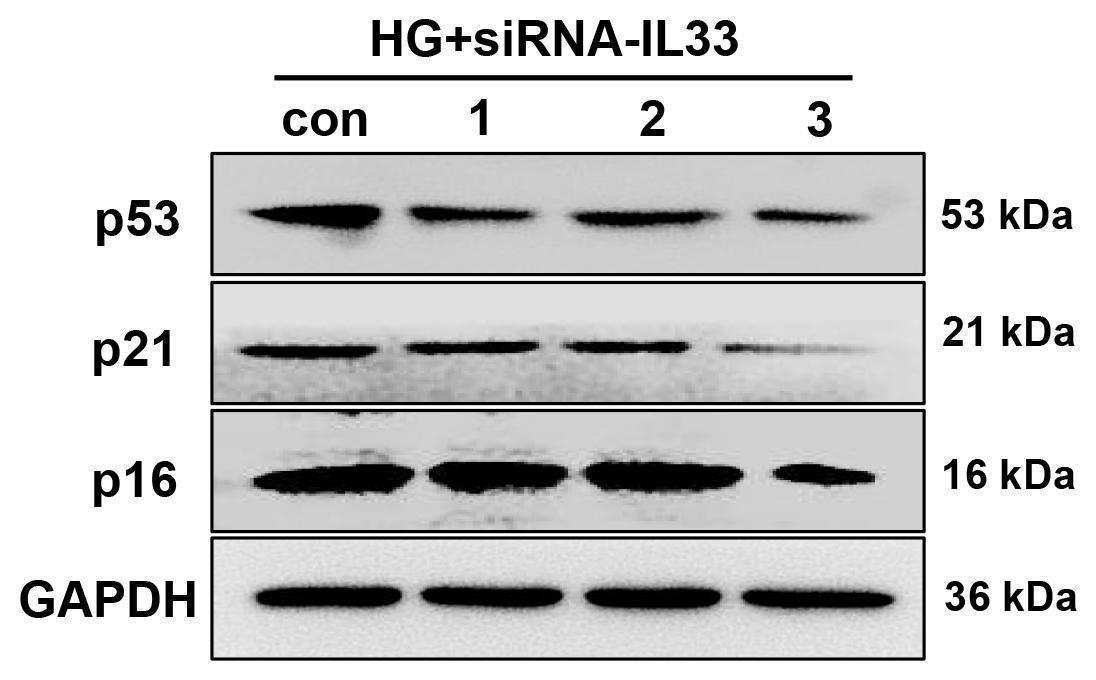

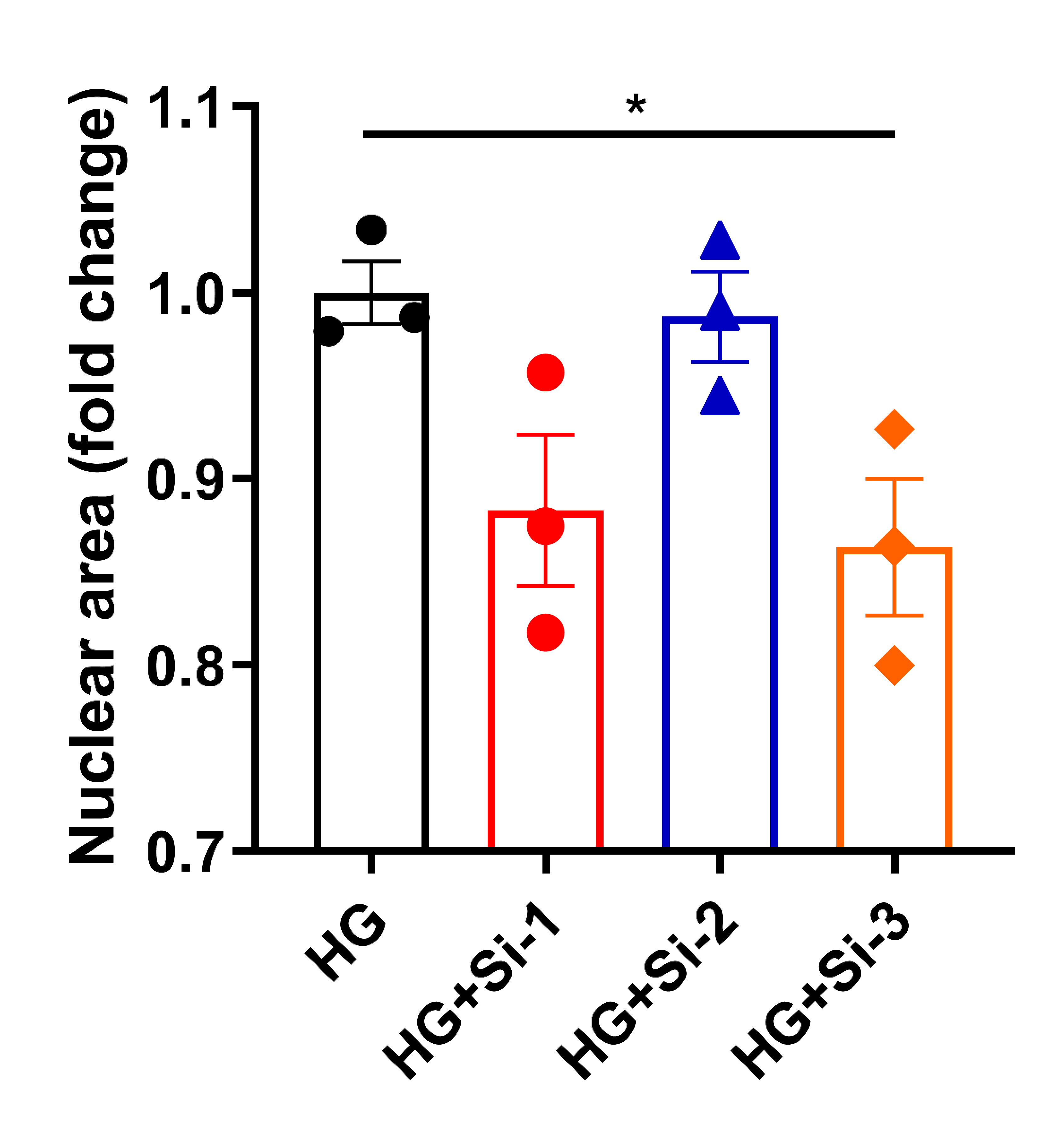

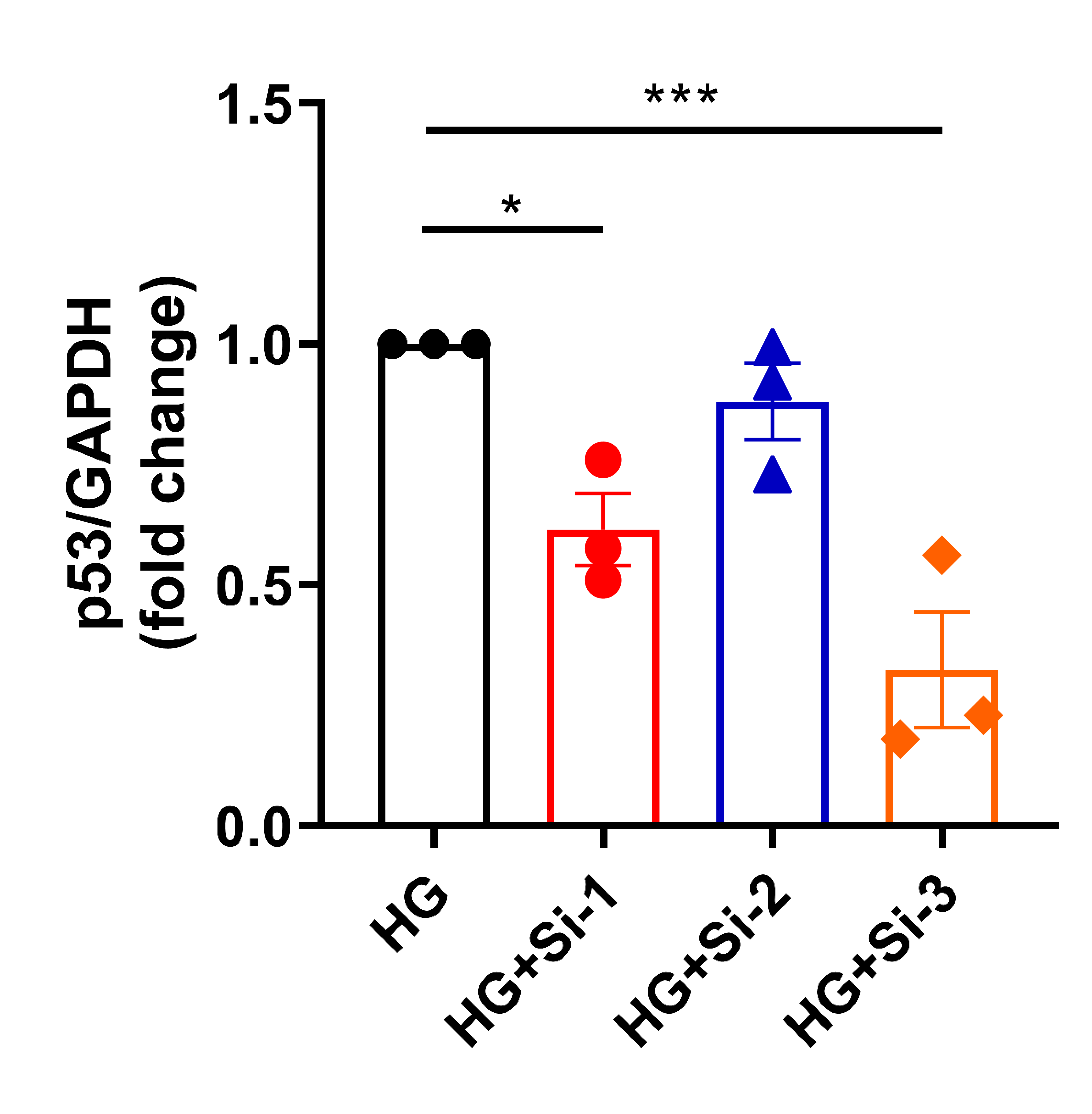

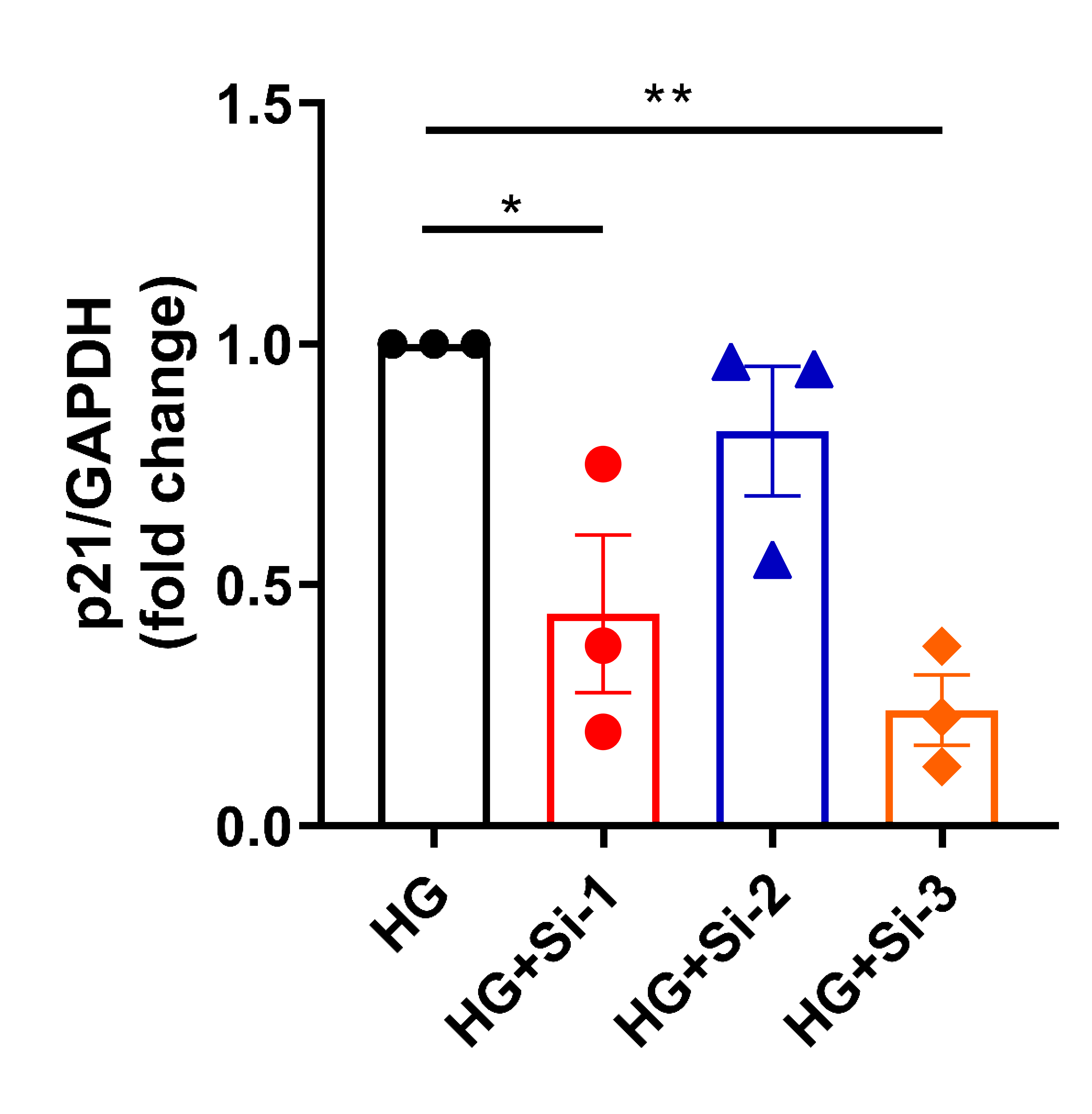

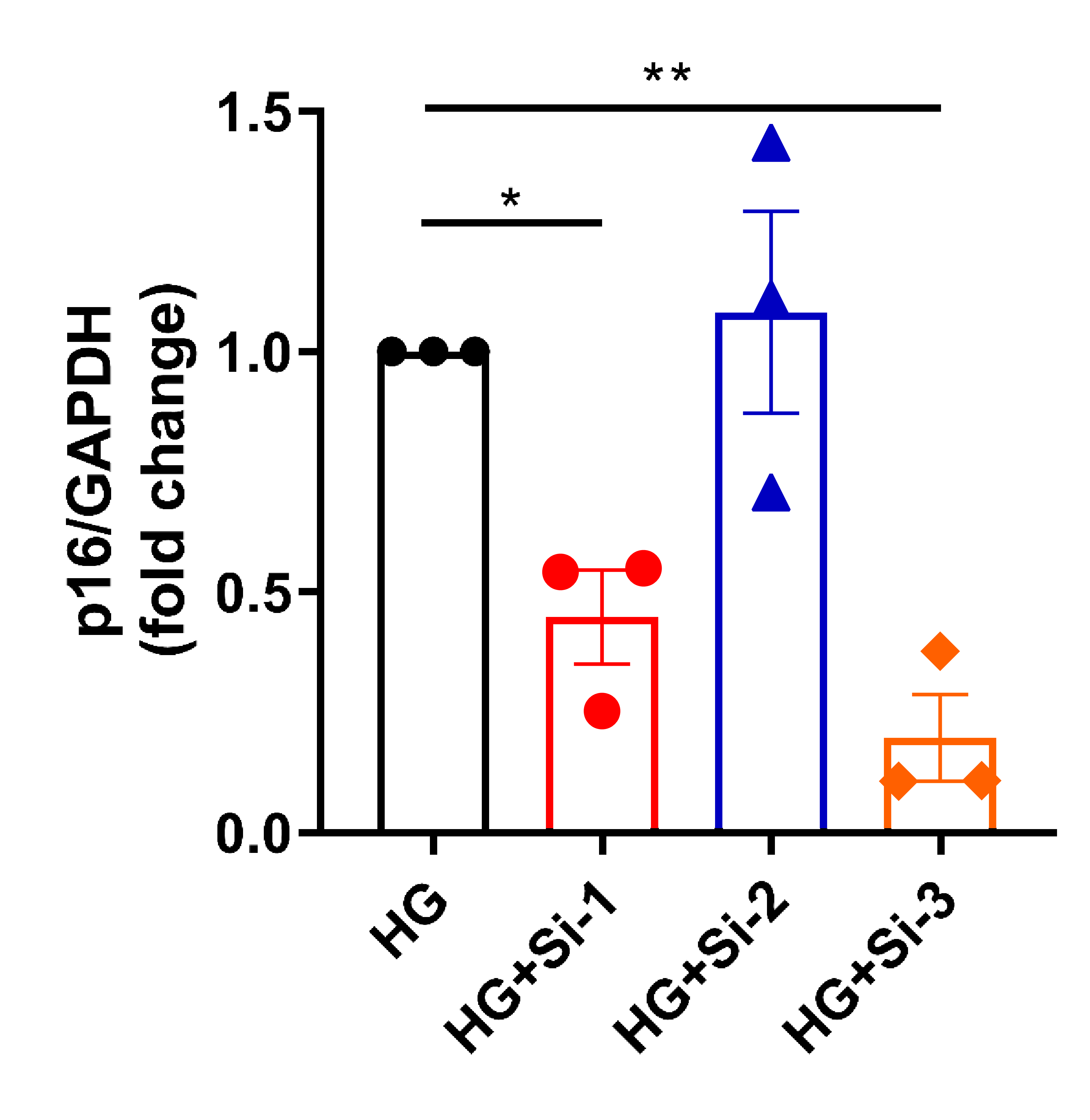

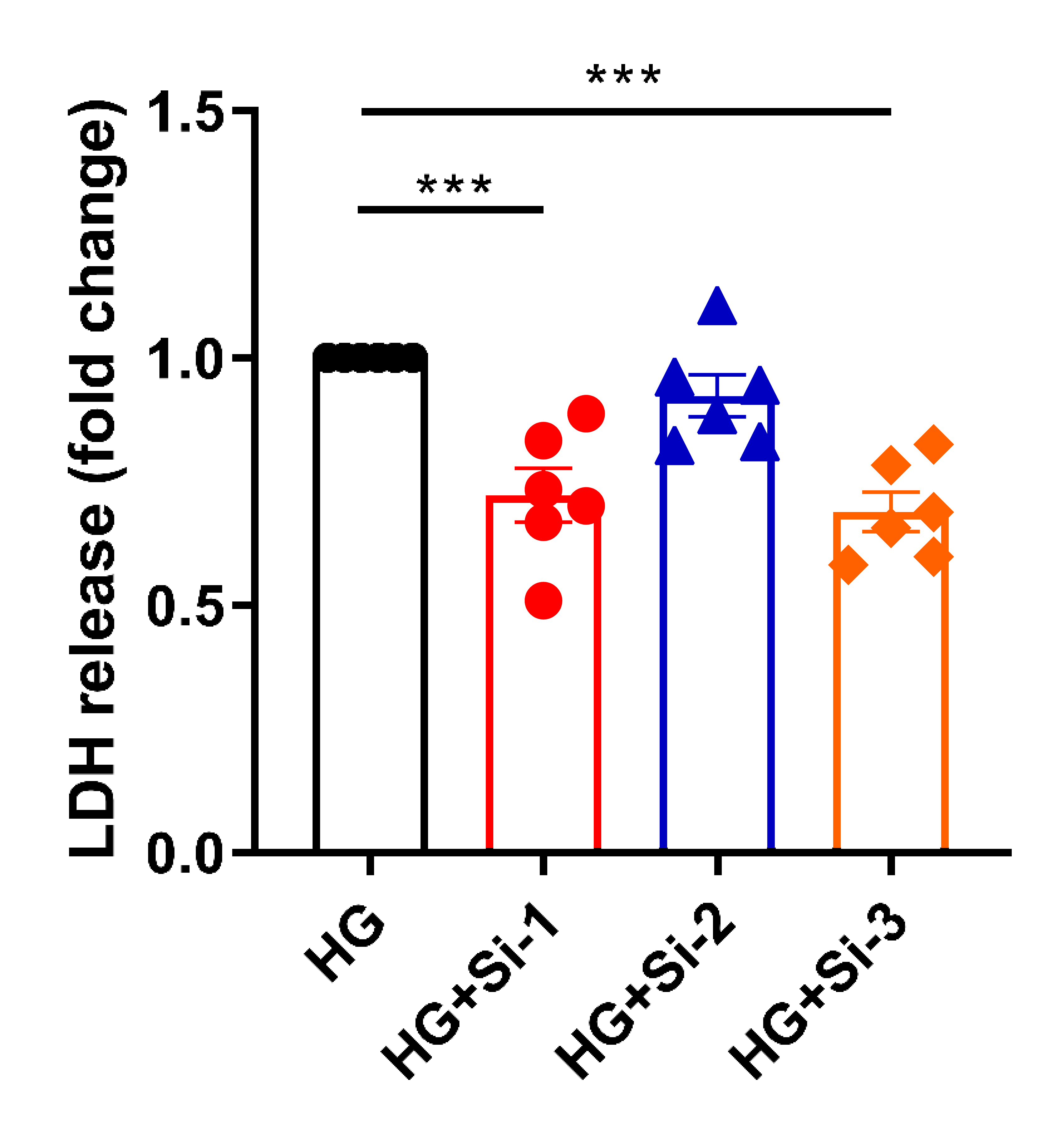

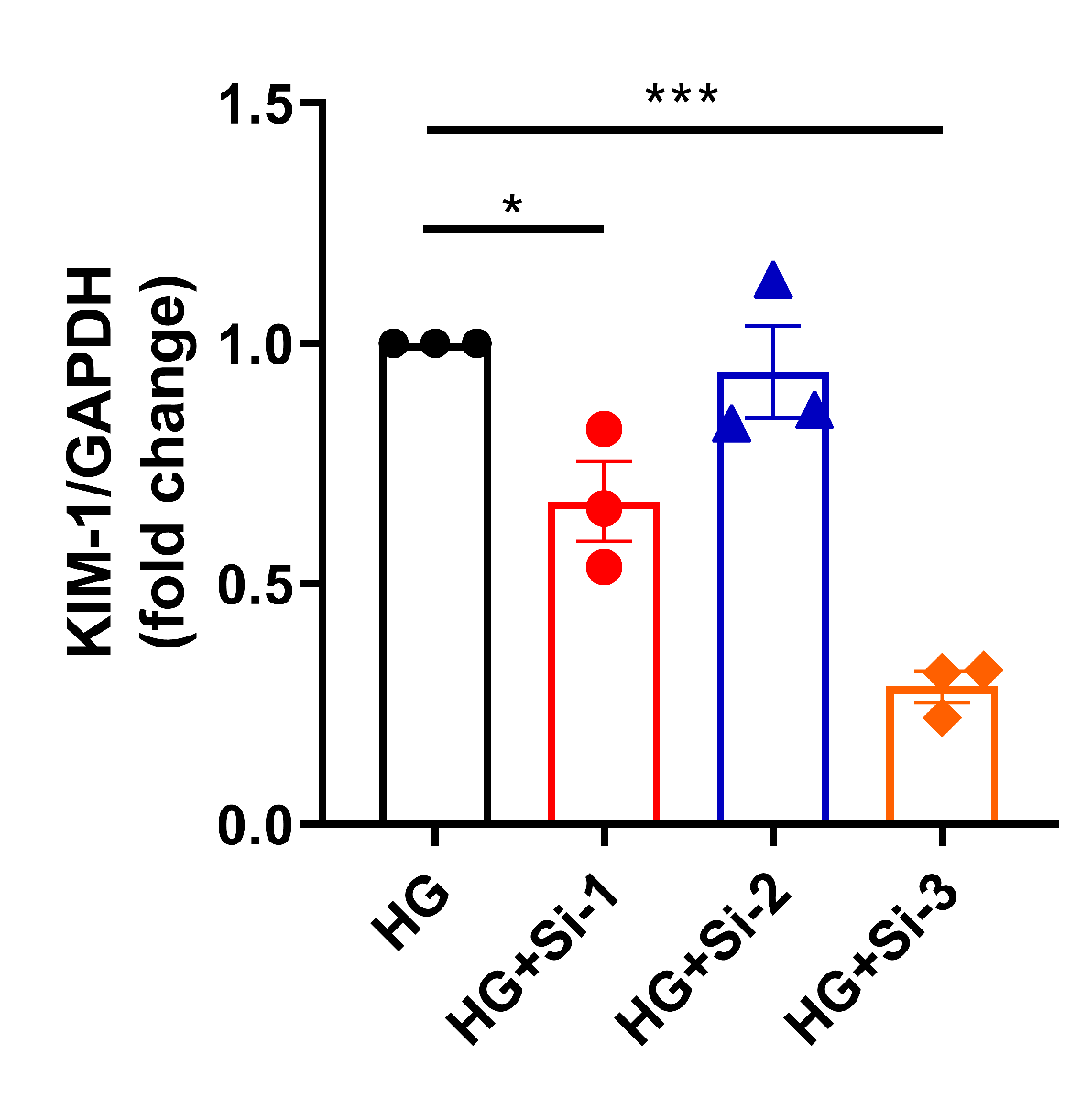

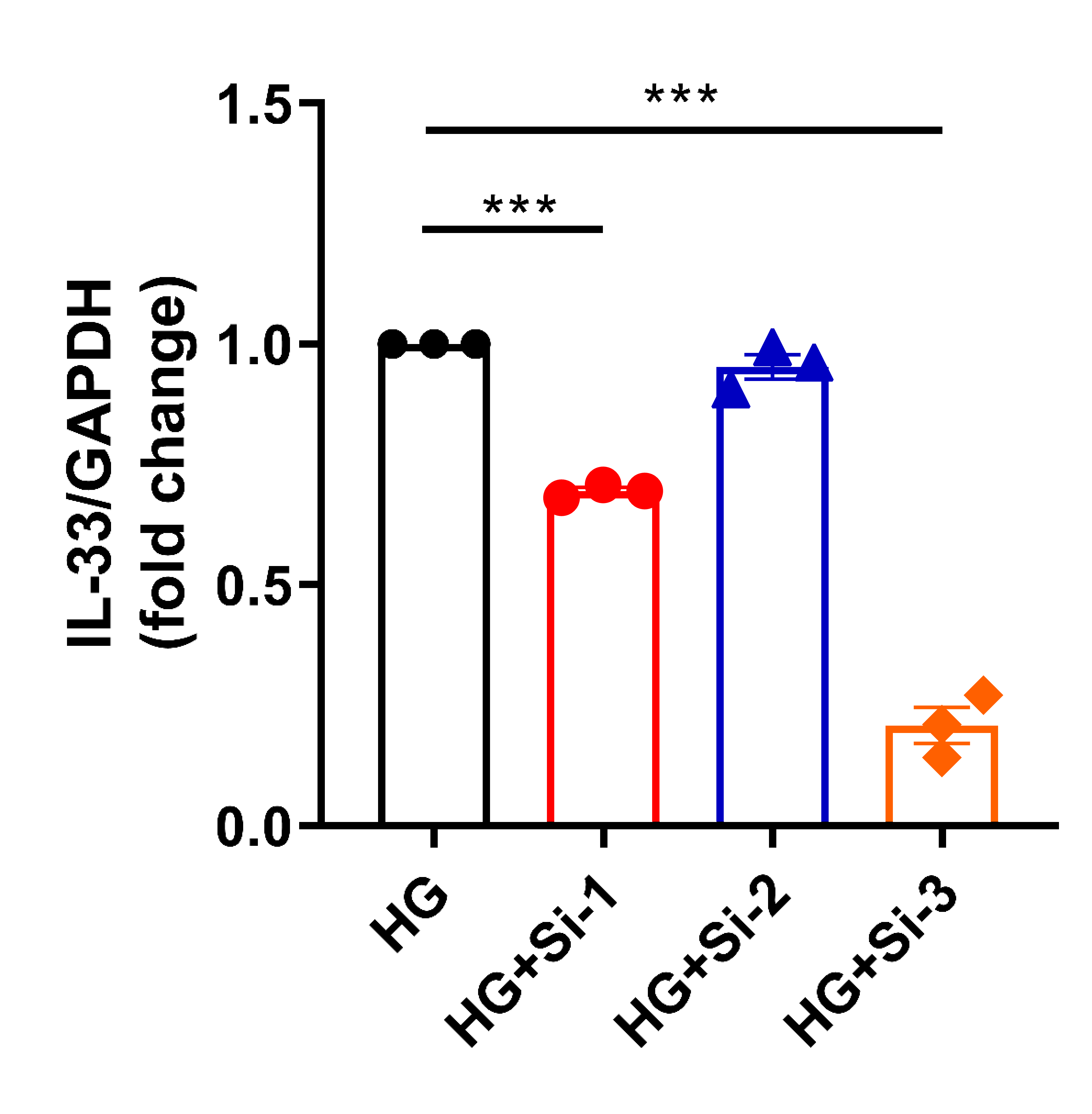

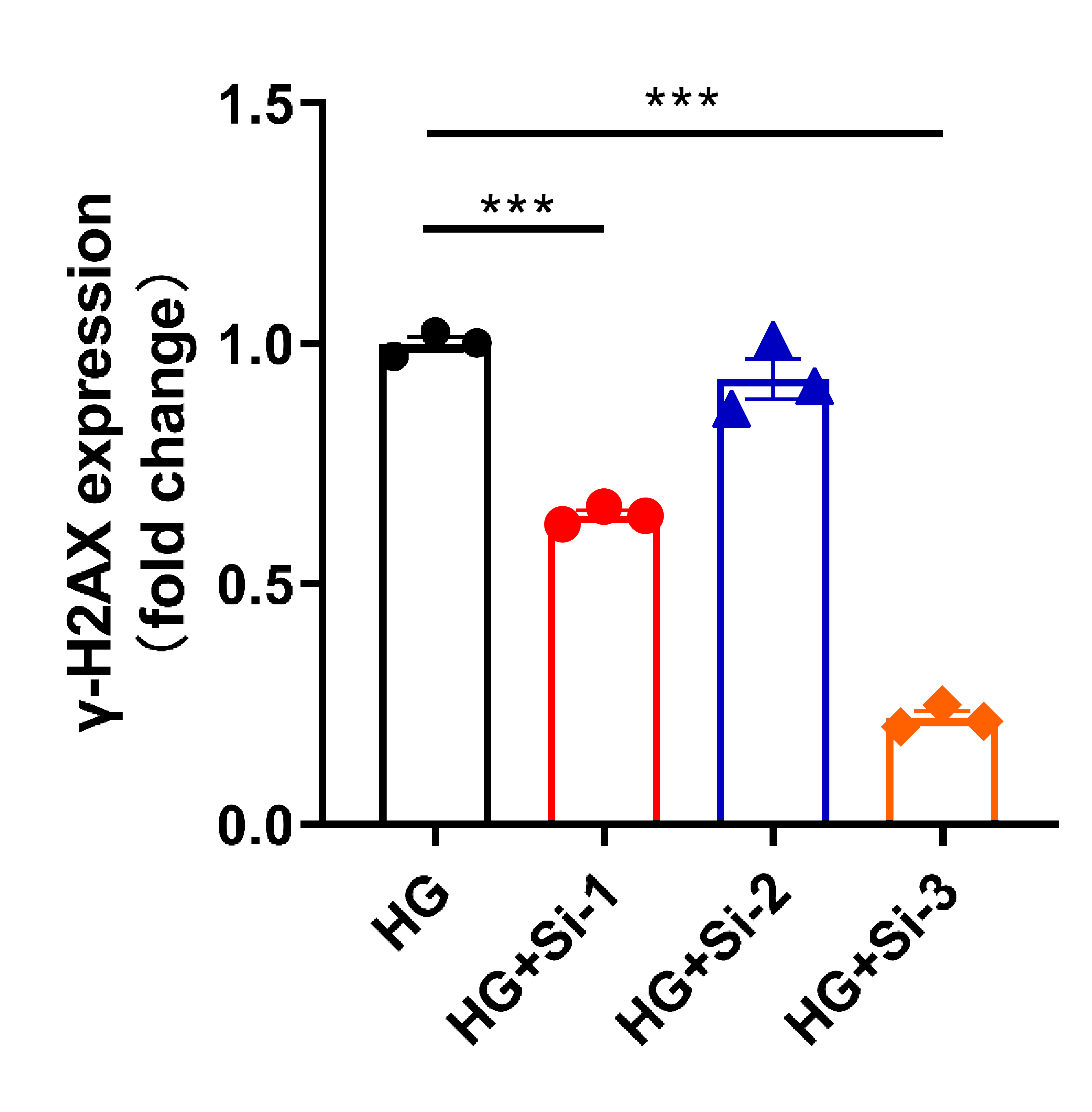

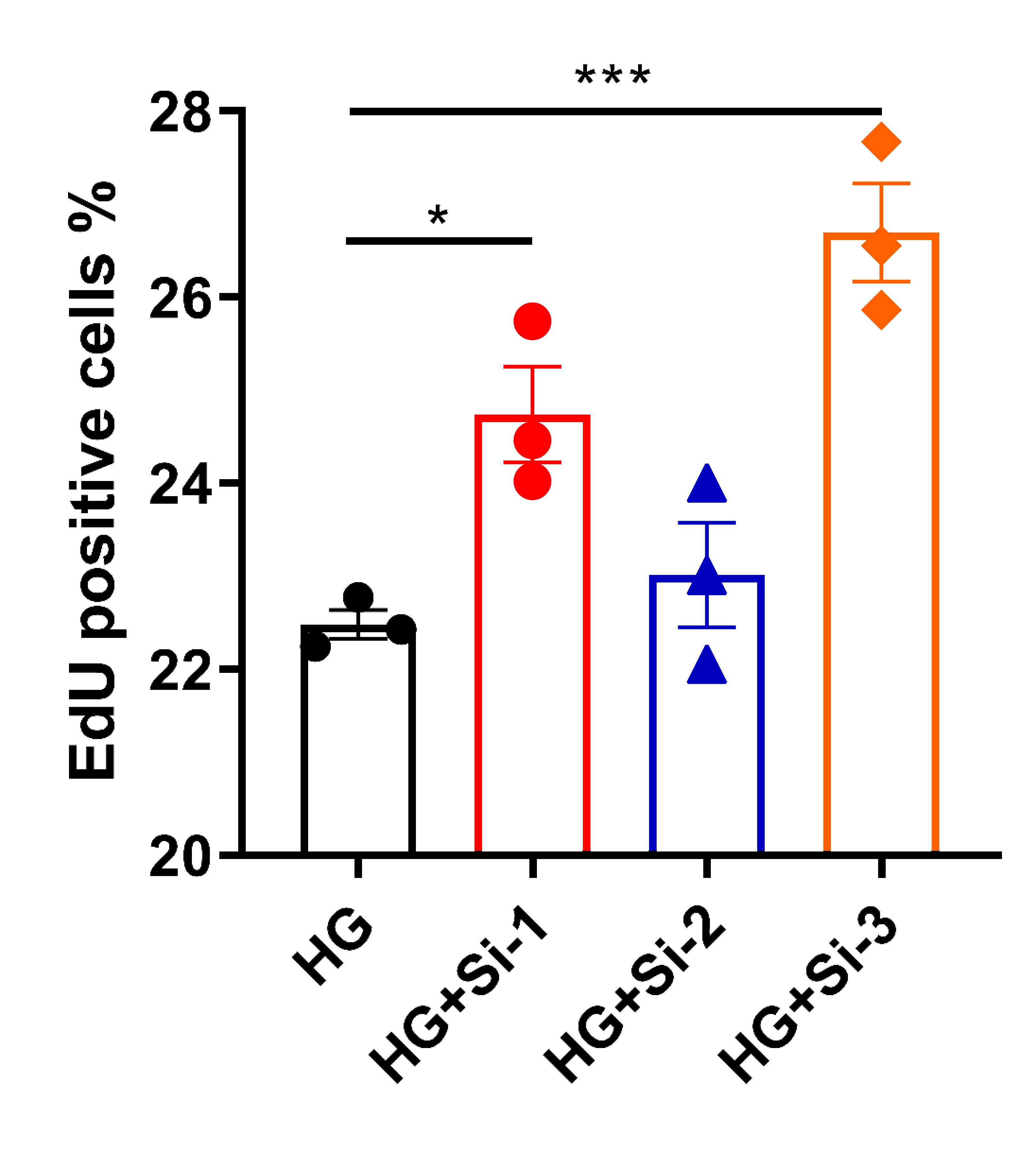

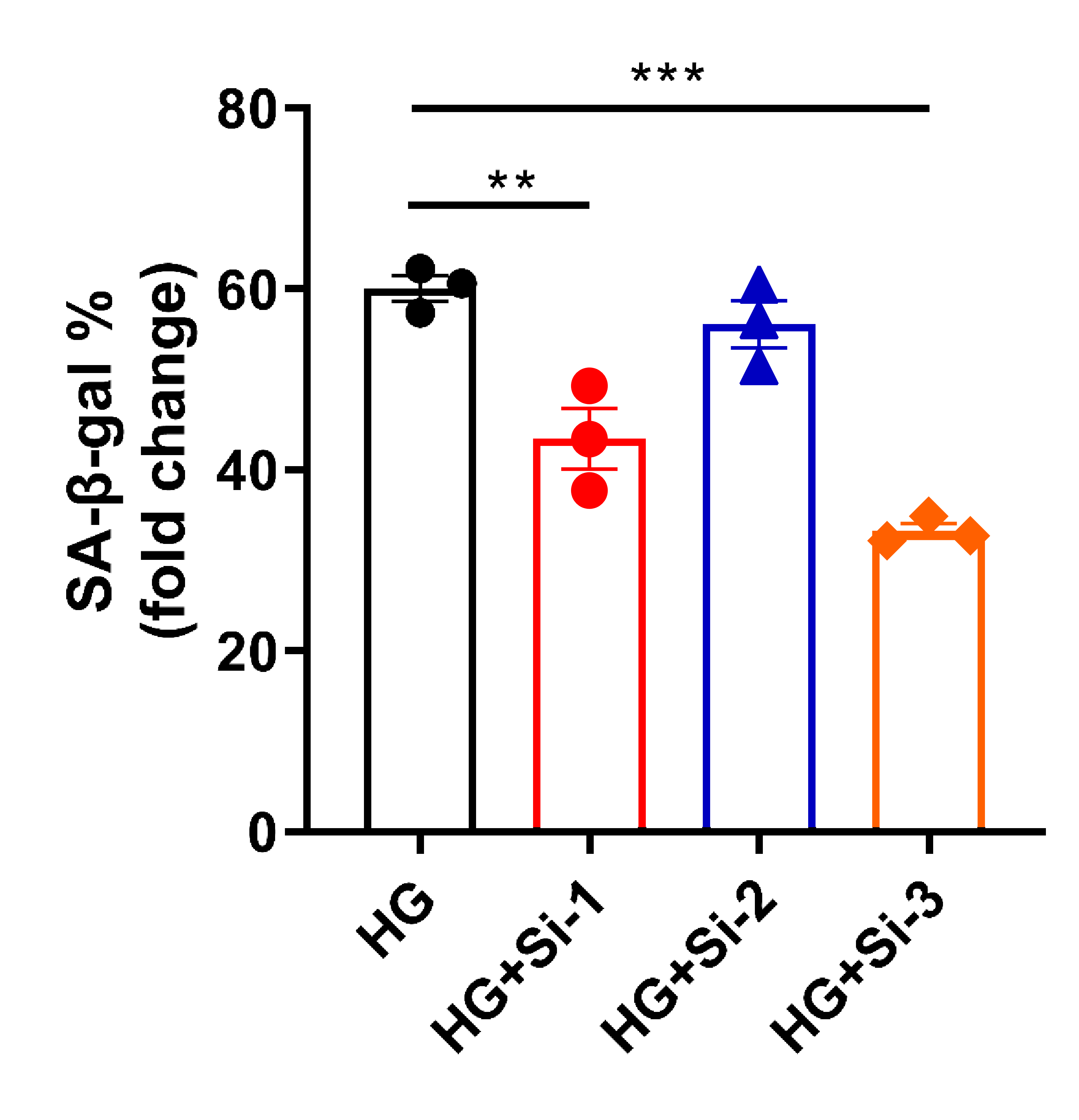


**B**

**D**

**E**

**C**

Figure S7. Partial reduction of IL-33 alleviates cell injury and senescence *in vitro.* (A) LDH release of NG-treated HK-2 cells exposed to rIL-33 (50 ng/mL) or αIL-33 (50 ng/mL). (B and C) KIM-1 expression and LDH release after different IL-33 sequence interference. (D) Cell senescence-related changes (SA-β-gal, EdU, nuclear area, and γH2AX) in HK-2 cells with IL-33 silencing. (E) Immunoblots and densitometric analysis of p53, p21, and p16. Scale bar: 20 μm. **P* < 0.05, ***P* < 0.01, ****P* < 0.001.

| **Table S1**. Basic characteristics between DN and non-DN participants in UK Biobank. | | | | |
| --- | --- | --- | --- | --- |
| Characteristics | Total (469777) | DN (6693) | Non-DN (463084) | *P* value |
| Age, (years) | 56.56 ± 8.08 | 62.18 ± 6.06 | 56.48 ± 8.08 | < 0.001 |
| Sex, n (%) |  |  |  | < 0.001 |
| Male | 214891 (45.74) | 3988 (59.58) | 210903 (45.54) |  |
| Female | 254886 (54.26) | 2705 (40.42) | 252181 (54.46) |  |
| Polygenic risk score | 0.287 ± 0.28 | 0.297 ± 0.28 | 0.287 ± 0.28 | 0.003 |
| Continuous variables were presented as mean ± SD; Categorical variables were presented as n (%). DN, diabetic nephropathy; n, numbers of subjects; %, weighted percentage. | | | | |

| **Table S2.** Basic characteristics of patients with DN and CON. | | | | | | |
| --- | --- | --- | --- | --- | --- | --- |
|  | Sex | Age  (yeas) | eGFR  (mL/min/1.73m^2^) | Creatinine  (μmol/L) | Blood urea nitrogen  (mmol/L) | Class |
| CON1 | Male | 63 | 47.8 | 135 | 12.20 | - |
| CON2 | Female | 66 | 55.3 | 93 | 4.11 | - |
| CON3 | Male | 69 | 51.8 | 122 | 8.80 | - |
| CON4 | Male | 62 | 72.7 | 96 | 3.60 | - |
| CON5 | Male | 55 | 97.6 | 76 | 5.00 | - |
| CON6 | Female | 57 | 102.8 | 51 | 5.90 | - |
| CON7 | Male | 60 | 64.7 | 107 | 4.80 | - |
| CON8 | Male | 79 | 58.6 | 104 | 2.30 | - |
| DN1 | Male | 55 | 5.1 | 893 | 47.48 | III |
| DN2 | Male | 69 | 41.1 | 147 | 7.70 | III |
| DN3 | Male | 24 | 123.3 | 77 | 6.24 | IIb |
| DN4 | Male | 36 | 123.2 | 59 | 9.10 | III |
| DN5 | Male | 57 | 63.9 | 110 | 9.50 | III |
| DN6 | Male | 60 | 9.6 | 519 | 13.82 | IV |
| DN7 | Female | 64 | 49.6 | 103 | 6.02 | IIa |
| DN8 | Male | 50 | 60.01 | 121 | 6.37 | IIb |
| DN9 | Male | 63 | 101.7 | 60 | 5.60 | I |
| DN10 | Male | 60 | 51.6 | 129 | 8.18 | III |
| DN11 | Male | 62 | 30.0 | 199 | 10.95 | III |
| DN12 | Female | 48 | 40.4 | 134 | 10.12 | III |
| DN13 | Female | 47 | 70.0 | 86 | 6.29 | III |
| DN14 | Male | 54 | 36.5 | 178 | 15.21 | III |
| DN, diabetic nephropathy; CON, kidney cancer patients; Class, classification of pathological diagnosis. | | | | | | |

| **Table S3.** Oxylipin profiles in DN mice and rIL-33-treated mice. | | | | | | | | | | |
| --- | --- | --- | --- | --- | --- | --- | --- | --- | --- | --- |
| **Metabolite Name** | **DN** | | | | | **DN+rIL-33** | | | | |
|  | **S-1** | **S-2** | **S-3** | **S-4** | **S-5** | **S-1** | **S-2** | **S-3** | **S-4** | **S-5** |
| 5,15-DIHETE | 0.032 | 0.042 | 0.079 | 0.046 | 0.045 | 0.096 | 0.178 | 0.054 | 0.040 | 0.074 |
| 5-HEPE | 0.062 | 0.210 | 0.178 | 0.043 | 0.036 | 0.015 | 0.231 | 0.027 | 0.050 | 0.107 |
| 12-HEPE | 0.205 | 0.219 | 0.266 | 0.423 | 0.295 | 0.271 | 1.946 | 0.163 | 0.212 | 0.631 |
| 15-HEPE | 0.011 | 0.053 | 0.013 | 0.043 | 0.017 | 0.031 | 0.131 | 0.038 | 0.076 | 0.354 |
| 18-HEPE | 0.018 | 0.075 | 0.055 | 0.003 | 0.008 | 0.008 | 0.107 | 0.004 | 0.002 | 0.043 |
| DHA | 326.8 | 331.6 | 169.0 | 232.8 | 196.1 | 217.5 | 198.6 | 209.5 | 191.6 | 174.6 |
| 4-HDHA | 1.287 | 5.290 | 6.411 | 0.210 | 0.459 | 0.388 | 5.074 | 0.305 | 0.152 | 8.212 |
| 7-HDHA | 0.119 | 0.357 | 0.391 | 0.038 | 0.050 | 0.058 | 0.616 | 0.043 | 0.045 | 0.421 |
| 14-HDHA | 0.368 | 0.760 | 0.876 | 0.624 | 0.387 | 0.563 | 4.076 | 0.542 | 0.784 | 1.125 |
| 17-HDHA | 0.268 | 0.706 | 0.684 | 0.090 | 0.100 | 0.161 | 1.015 | 0.118 | 0.152 | 0.949 |
| 9-HETE | 0.081 | 0.165 | 0.672 | 0.093 | 0.075 | 0.159 | 0.753 | 0.092 | 0.091 | 0.113 |
| 18-HETE | 0.006 | 0.012 | 0.023 | 0.008 | 0.018 | 0.010 | 0.027 | 0.007 | 0.005 | 0.021 |
| 16-HETE | 0.015 | 0.031 | 0.158 | 0.006 | 0.024 | 0.003 | 0.116 | 0.002 | 0.006 | 0.095 |
| PGJ2 | 0.026 | 0.034 | 0.047 | 0.072 | 0.053 | 0.065 | 0.085 | 0.028 | 0.041 | 0.030 |
| PGD2 | 1.610 | 2.948 | 3.480 | 1.705 | 3.067 | 5.216 | 8.512 | 2.880 | 4.976 | 3.948 |
| PGF2a | 0.307 | 0.449 | 0.426 | 0.685 | 0.441 | 0.667 | 0.899 | 0.523 | 0.459 | 0.458 |
| 11,12-EET | 0.240 | 0.611 | 1.453 | 0.102 | 0.117 | 0.153 | 0.553 | 0.102 | 0.060 | 1.031 |
| 5-HETE | 0.905 | 2.803 | 8.579 | 0.342 | 0.746 | 0.480 | 8.339 | 0.372 | 0.422 | 6.117 |
| 15-HETE | 1.061 | 2.417 | 6.442 | 1.267 | 1.531 | 2.468 | 9.607 | 1.311 | 1.581 | 6.326 |
| PGE2 | 1.906 | 2.948 | 3.579 | 1.705 | 3.213 | 5.065 | 8.647 | 2.936 | 4.895 | 4.057 |
| PGF1a | 0.023 | 0.025 | 0.020 | 0.027 | 0.012 | 0.031 | 0.047 | 0.020 | 0.024 | 0.017 |
| 6-Keto-PGF1a | 0.802 | 1.509 | 1.053 | 1.248 | 1.646 | 1.916 | 1.850 | 1.457 | 1.707 | 1.529 |
| 8-HETE | 0.191 | 0.548 | 1.636 | 0.119 | 0.205 | 0.196 | 1.855 | 0.160 | 0.155 | 1.261 |
| 11-HETE | 7.820 | 11.61 | 15.54 | 12.18 | 11.15 | 21.37 | 33.06 | 13.47 | 14.70 | 20.94 |
| 12-HETE | 1.786 | 1.712 | 5.923 | 4.731 | 3.778 | 3.841 | 16.26 | 3.036 | 3.083 | 8.400 |
| 5,6-EET | 0.166 | 0.389 | 0.821 | 0.044 | 0.071 | 0.074 | 0.362 | 0.055 | 0.028 | 0.628 |
| 8,9-EET | 0.130 | 0.314 | 0.794 | 0.050 | 0.065 | 0.082 | 0.272 | 0.040 | 0.028 | 0.578 |
| 14,15-EET | 0.073 | 0.249 | 0.919 | 0.022 | 0.044 | 0.025 | 0.222 | 0.015 | 0.003 | 0.547 |
| PGD3 | 0.008 | 0.047 | 0.015 | 0.006 | 0.018 | 0.029 | 0.116 | 0.013 | 0.029 | 0.089 |
| TXB2 | 2.084 | 2.614 | 2.320 | 2.809 | 3.191 | 4.188 | 4.614 | 2.723 | 4.010 | 3.221 |
| LXA4 | 0.038 | 0.404 | 1.525 | 0.003 | 0.004 | 0.001 | 0.588 | 0.005 | 0.002 | 0.652 |
| RVE1 | 0.001 | 0.004 | 0.006 | 0.001 | 0.001 | 0.000 | 0.027 | 0.000 | 0.001 | 0.021 |
| RVD1 | 0.000 | 0.011 | 0.013 | 0.000 | 0.001 | 0.001 | 0.022 | 0.001 | 0.001 | 0.001 |
| 5S,6r-DIHETE | 0.058 | 0.284 | 1.194 | 0.003 | 0.031 | 0.017 | 0.295 | 0.007 | 0.006 | 1.475 |
| 13-oxoDDE | 11.41 | 27.64 | 39.38 | 3.068 | 7.592 | 4.131 | 9.842 | 9.320 | 4.470 | 39.85 |
| 9-oxoDDE | 4.620 | 10.41 | 15.72 | 1.015 | 2.498 | 1.075 | 3.412 | 1.749 | 1.469 | 13.37 |
| PGD1 | 0.020 | 0.045 | 0.069 | 0.027 | 0.052 | 0.070 | 0.148 | 0.047 | 0.089 | 0.060 |
| PGE1 | 0.045 | 0.071 | 0.068 | 0.070 | 0.058 | 0.102 | 0.150 | 0.079 | 0.085 | 0.082 |
| 9(s)-HODE | 3.023 | 5.718 | 4.028 | 4.831 | 3.283 | 4.791 | 26.00 | 3.065 | 9.969 | 3.874 |
| 13-HODE | 2.743 | 6.765 | 5.737 | 5.988 | 2.970 | 3.833 | 30.45 | 3.314 | 8.573 | 4.814 |
| 9(10)-EPOME | 0.548 | 1.171 | 0.873 | 0.387 | 0.199 | 0.249 | 1.446 | 0.172 | 0.448 | 0.995 |
| 12(13)-EPOME | 0.289 | 0.615 | 0.494 | 0.228 | 0.122 | 0.126 | 0.687 | 0.093 | 0.244 | 0.502 |
| 9-HpODE | 0.006 | 0.592 | 0.369 | 0.010 | 0.002 | 0.001 | 0.370 | 0.004 | 0.004 | 1.326 |
| 13-HpODE | 0.005 | 1.204 | 0.651 | 0.001 | 0.001 | 0.000 | 0.636 | 0.002 | 0.004 | 1.918 |
| 9(s)-HOTrE | 0.036 | 0.065 | 0.066 | 0.032 | 0.018 | 0.016 | 0.510 | 0.015 | 0.044 | 0.048 |
| 13(s)-HOTrE | 0.024 | 0.025 | 0.030 | 0.147 | 0.024 | 0.035 | 0.191 | 0.071 | 0.115 | 0.011 |
| 9-oxoOTrE | 0.214 | 0.374 | 0.610 | 0.048 | 0.069 | 0.003 | 0.142 | 0.008 | 0.008 | 0.426 |
| 9(s)-HpOTrE | 0.006 | 0.038 | 0.043 | 0.001 | 0.001 | 0.001 | 0.029 | 0.000 | 0.001 | 0.038 |
| 9(S),10(S).13(S)-TriHOME | 0.293 | 0.510 | 0.334 | 0.426 | 0.427 | 0.324 | 8.233 | 0.199 | 1.032 | 0.463 |
| 9,10-EpODE | 0.008 | 0.052 | 0.062 | 0.008 | 0.004 | 0.000 | 0.066 | 0.000 | 0.004 | 0.037 |
| 12,13-EpODE | 0.011 | 0.023 | 0.023 | 0.011 | 0.002 | 0.003 | 0.014 | 0.000 | 0.011 | 0.024 |
| 15,16-EpODE | 0.019 | 0.038 | 0.030 | 0.047 | 0.005 | 0.006 | 0.026 | 0.001 | 0.017 | 0.017 |
| 9,10-DiHODE | 0.007 | 0.001 | 0.007 | 0.038 | 0.006 | 0.005 | 0.015 | 0.006 | 0.024 | 0.002 |
| 12,13-DiHODE | 0.001 | 0.001 | 0.000 | 0.006 | 0.002 | 0.000 | 0.003 | 0.001 | 0.000 | 0.001 |
| 15,16-DiHODE | 0.061 | 0.021 | 0.056 | 0.555 | 0.057 | 0.017 | 0.060 | 0.055 | 0.360 | 0.018 |
| 12-HOME | 0.068 | 0.029 | 0.050 | 0.256 | 0.076 | 0.066 | 0.120 | 0.125 | 0.138 | 0.050 |
